# Tandem repeats structure of gel-forming mucin domains could be revealed by SMRT sequencing data

**DOI:** 10.1038/s41598-022-25262-7

**Published:** 2022-11-30

**Authors:** Tiange Lang

**Affiliations:** Big Data Decision Institution, Jinan University, No 601 Huangpu Avenue West, Tianhe District, Guangzhou, 510632 China

**Keywords:** Biotechnology, Genetics

## Abstract

Mucins are large glycoproteins that cover and protect epithelial surface of the body. Mucin domains of gel-forming mucins are rich in proline, threonine, and serine that are heavily glycosylated. These domains show great complexity with tandem repeats, thus make it difficult to study the sequences. With the coming of single molecule real-time (SMRT) sequencing technologies, we manage to present sequence structure of mucin domains via SMRT long reads for gel-forming mucins MUC2, MUC5AC, MUC5B and MUC6. Our study shows that for different individuals, single nucleotide polymorphisms could be found in mucin domains of MUC2, MUC5AC, MUC5B and MUC6, while different number of tandem repeats could be found in mucin domains of MUC2 and MUC6. Furthermore, we get the sequence of MUC2, MUC5AC, and MUC5B mucin domain in a Chinese individual for each nucleotide at accuracy of possibly 99.98–99.99%, 99.93–99.99%, and 99.76–99.99%, respectively. We report a new method to obtain DNA sequence of gel-forming mucin domains. This method will provided new insights on getting the sequence for Tandem Repeat parts which locate in coding region. With the sequences we obtained through this method, we can give more information for people to study the sequences of gel-forming mucin domains.

## Introduction

The gel-forming mucins are large glycosylated proteins essential for mucus layer to cover epithelial cells^1^. The gel-like mucus could protect against harmful microorganisms^2^. There are five gel-forming mucins in mammals^1^. They are MUC2, MUC5AC, MUC5B, MUC6 and MUC19. Each protein contains a mucin domain which is rich in amino acids proline (P), threonine (T) and serine (S). The mucin domain, also called PTS domain, is heavily glycosylated and therefore has a stiff conformation which could give the mucin protein the function of protection^3^.

For each gel-forming mucin, the sequence before the mucin domain is called N-terminal sequence and the sequence afterwards is called C-terminal sequence. All the N-terminal sequences of gel-forming mucins share a similar structure which contains three VWD domains, and all the C-terminal sequences have Cysteine knot (CK) domains^4–7^. Both the cysteine number and their positions are extremely conserved in those domains, which play an essential role in forming dimers and trimers^7^.

In human, the genes of MUC2, MUC5AC, MUC5B and MUC6 are clustered in a complex of 400 kb very rich in CpG islands on chromosome 11 in region p15.5^8,9^. Computational and phylogenetic analyses suggested an evolutionary history of the four human mucin genes from an ancestor gene common to the human von Willebrand factor gene^7,10^.

In human, DNA sequence of mucin domain in MUC2/MUC5AC/MUC5B/MUC6 is only one exon. This exon is organized in tandem repeats. These tandem repeats are very large, and the length of each repeat could vary^9,11^. Therefore, it is very difficult to get the exact sequence of this part.

SMRT DNA sequencing technology could generate reads up to 10–20 k bases^12^. This length could span the whole mucin domain. Therefore, the sequence of mucin domain could be assembled with SMRT reads^13^. However, this technology has high error rate^14^. Thus a high coverage of the reads is needed to obtain the sequence of mucin domain^15^.

Several human reference genomes are available, and these could be used to locate mucin domains^13,14,16^. Moreover, a whole human genome SMRT reads of a Chinese individual (HX1) are available on the website http//:hx1.wglab.org/, and this could be used to make the assembly of mucin domains^13^. In our work, by combining with a new method we constructed, we take advantage of these data in obtaining the exact full sequences of MUC2, MUC5AC, and MUC5B mucin domains in HX1 which are extremely difficult to get due to their tandem repeats structure. Meanwhile, SNP and CNV information of mucin domains of MUC2, MUC5AC, MUC5B and MUC6 is also demonstrated in HX1.

## Methods

### Downloading of Chr11p15.5 of human genome of reference sequence (Refseq), a Korean individual, and an American individual

From NCBI, we downloaded whole chromosome 11 of human genome of Refseq, a Korean individual (BioProject PRJNA294231), and an American individual (BioProject PRJNA294231). Then we extracted whole chromosome 11 from 1 to 1.4 M.

In Refseq, this region includes six genes. They are APA2 (forward strand, position 925,809–1,012,245), MUC6 (reverse strand, position 1,012,823–1,036,706), MUC2 (forward strand, position 1,074,875–1,110,508), MUC5AC (forward strand, position 1,157,953–1,201,138), MUC5B (forward strand, position 1,239,777–1,249,564), and TOLLIP (reverse strand, position 1,274,368–1,309,662).

### Downloading of whole human genome sequence read archive (SRA) data of a Chinese individual (HX1) and converting of SRA data to fasta sequence data

We downloaded whole human genome SRA data of a Chinese individual (HX1) from NCBI. There are two types of SRA data. One is PACBIO_SMRT data from PacBio RS II, and the other is ILLUMINA data from Illumina Hiseq 2000. HX1 human genome assembly was made with Illumina Hiseq data which has 2.8 billion reads (N50 length 151) as well as PacBio SMRT data which has 44.2 million reads (N50 length 12.1 k). We only downloaded PACBIO_SMRT data and the size is 2773.6Gbp.

For the genome data of HX1 downloaded, we used perl and python scripts to convert SRA data to fasta sequence data (Supplementary S1 and S2). In the converting process, we only kept the reads which were longer than 5 k bases. The number of reads kept was 24,290,526. The number of bases kept was 284,581,254,229, and that was about 94.86X coverage.

### Obtaining of mucin domain of MUC2/MUC5AC/MUC5B/ MUC6 in the assembly of Refseq, Korean individual and American individual

In human genome, the mucin domains of MUC2/MUC5AC/MUC5B/ MUC6 have only one exon. Thus first we found the DNA sequence of mucin domains in NCBI MUC2 (Accession number NM_002457.4), MUC5AC (Accession number NM_001304359.1), MUC5B (Accession number NM_002458.2), and MUC6 (Accession number NM_005961.2). Next we used the sequence found as query sequences doing similarity search into genome assembly of Refseq in Chr11p15.5, Korean individual in Chr11 and American individual.

### Obtaining of mucin domain of MUC2/MUC5AC/MUC5B/ MUC6 in the SMRT reads of HX1

First we found the DNA sequence of the exons of mucin domains in NCBI MUC2 (Accession number NM_002457.4), MUC5AC (Accession number NM_001304359.1), MUC5B (Accession number NM_002458.2) and MUC6 (Accession number NM_005961.2). Next for each mucin, we took introns up-stream and down-stream of the mucin domain exon. Then we used the combination of the three fragments as query sequences doing similarity search into SMRT reads of HX1.

### Assembly of mucin domain of MUC2, MUC5AC, MUC5B and MUC6 in HX1 with SMRT reads

Due to the TR character, it is hard for CLUSTALW to make a good alignment if only PTS exon was used. Therefore, it is essential to include the introns up-stream and down-stream.

For MUC2, in all the SMRT reads downloaded, 4 reads could be found to cover introns both up-stream and down-stream of mucin domain exon. In human MUC2 mucin domain, there is a CysD domain in the middle of a domain which is full of proline, threonine and serine (PTS). Although TR structure of PTS domain makes it impossible to be identified accurately with similarity search, CysD domain could be precisely found. Therefore, we took use of the CysD domain in the middle of PTS exon. We regarded the last “cysteine” in CysD domain as delimiter. The first part could contain the intron up-stream, PTS TRs, and the CysD island, and the second part could contain the CysD island, PTS TRs, and the intron down-stream. Thus we searched for two types of read: I. intron up-stream mucin domain exon + up-stream part of mucin domain exon; II. down-stream part of mucin domain exon + intron down-stream mucin domain exon. We found 18 type I and 9 type II reads. Combining with the 4 reads which could be found to cover introns both up-stream and down-stream, 22 reads could be used to build up-stream part and 13 reads could be used to build down-stream part.

For MUC5AC, MUC5B and MUC6, we could not split the mucin domain exons and had to find the reads which could cover introns both up-stream and down-stream of mucin domain exon. In all the SMRT reads downloaded, 10, 9, and 3 reads could be found for MUC5AC, MUC5B and MUC6, respectively.

### Bioinformatic methods

For similarity search we used BLAST^17^. For multiple sequence alignment we used CLUSTALW^18^. Genetic codon table for human was used for translation. HMMER was used for identifying protein domains^19^. Perl and python scripts were used for other tasks (Supplementary materials).

For CLUSTALW, we set gap_opening_penalty and gap_extension_penalty to 0. The reason is that if you consider to align two sequences with different TRs numbers, the default numbers of gap_opening_penalty and gap_extension_penalty in CLUSTALW will cause the interruption of one complete repeat unit. However, the alignment of two common sequences (none TR) has no such problem.

## Results

### Mucin domain of MUC2/MUC5AC/MUC5B/ MUC6 in Refseq, Korean, and American assembly

The length of mucin domains of MUC2 of Refseq, Korean and American assembly are 5884 bases, 4471 bases and 8773 bases, respectively. In NCBI there is a nucleotide entry of human MUC2 DNA sequence with Accession number NM_002457.4. The mucin domain of this entry is 9270 bases and only a part of it could be found in Refseq. We regard the mucin domain of NM_002457.4 as the most complete assembly among all available MUC2 mucin domain assemblies by similarity search.

The length of mucin domains of MUC5AC of Refseq, Korean and American assembly are 10,371 bases, 10,576 bases and 11,196 bases, respectively. We regard the mucin domain of MUC5AC in Refseq as the most accurate assembly among all the three assemblies by similarity search.

The length of mucin domains of MUC5B of Refseq, Korean and American assembly are 10,893 bases, 11,589 bases and 10,772 bases, respectively. We regard the mucin domain of MUC5B in Refseq as the most accurate assembly among all the three assemblies by similarity search.

The lengths of mucin domains in MUC6 of Refseq, Korean and American assembly are 3009 bases, 13,299 bases and 8727 bases, respectively. For the American mucin domain of MUC6, the beginning sequence goes into gap region. We regard the mucin domain of MUC6 in Korean individual as the most complete assembly among the three assemblies by similarity search.

We could see that even by length, these domains in each of the MUC2, MUC5AC, MUC5B and MUC6 make some differences (Table 1).

**Table 1:** Length comparison of mucin domain exons of MUC2, MUC5AC, MUC5B and MUC6 in Refseq, Korean assembly, American assembly, HX1 own assembly and HX1 assembly with our pipeline.

Assemblies	Length of MUC2 mucin domain exon	Length of MUC5AC mucin domain exon	Length of MUC5B mucin domain exon	Length of MUC6 mucin domain exon
Refseq	5884 bases	10,371 bases	10,893 bases	3009 bases
Korean assembly	4471 bases	10,576 bases	11,589 bases	13,299 bases
American assembly	8773 bases	11,196 bases	10,772 bases	8727 bases
HX1 own assembly	None	10,368 bases	10,789 bases	11,992 bases
HX1 assembly by our pipeline	8994 bases	10,371 bases	10,893 bases	13,470 bases

We can see that the length of the domains in each mucin make differences, and the domain constructed from our pipeline has the more length than HX1 own assembly for each mucin.

### Programing pipelines to get consensus sequence with SMRT reads

The PacBio SMRT data of HX1 human genome was used to get the mucin domains. Since the whole data set has 44.2 million reads, the data with a size of several tens of Terabytes was dealt with. Therefore, it is essential to make an efficient pipeline to obtain the reads which could be used to assemble the mucin domains.

**Figure 1:**
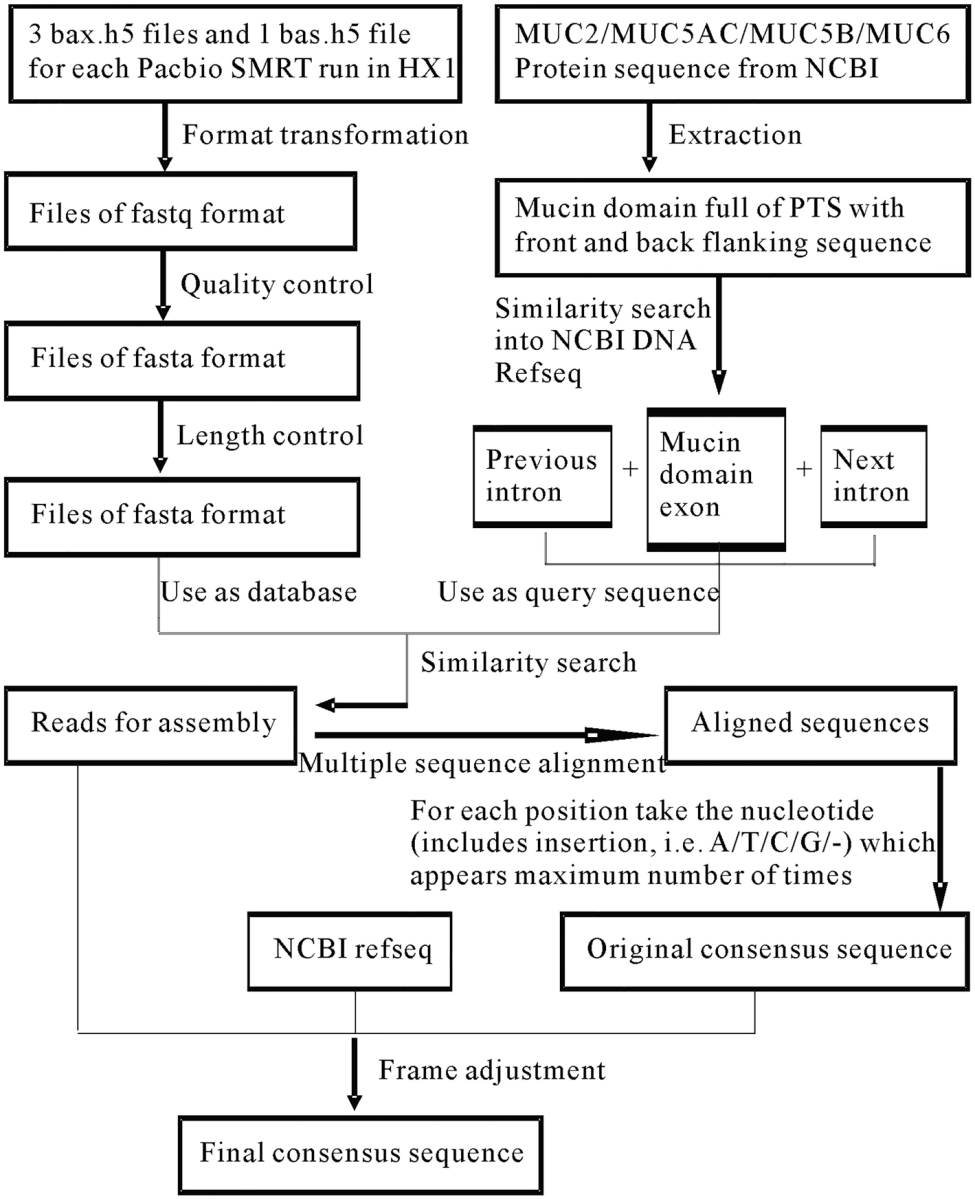
Programing pipeline to get consensus with SMRT reads. For SMRT reads from HX1, original files downloaded were bax.h5 files and bas.h5 files. These files were transferred into fastq files. Then they were trimmed by quality control and length control methods. Meanwhile, DNA sequence of mucin domain exons of MUC2, MUC5AC, MUC5B and MUC6 were extracted from Refseq by comparing with their protein sequences which were also downloaded from NCBI. Next the similarity search were performed using both SMRT reads and extracted Refseq to get reads which could be used to build the mucin domain. After multiple sequence alignment and maximum number count, original consensus sequences could be obtained. After frame adjustment, final consensus sequences were constructed.

The SMRT reads downloaded were bax.h5 files and bas.h5 files. We transferred them into fastq files for further treatments. We used quality control and length control methods on those fastq files with programming scripts (Supplementary S3 and S4). By similarity search with BLAST to the Refseq sequences of MUC2, MUC5AC, MUC5B and MUC6 which could be downloaded from NCBI in both DNA and protein level, we managed to find reads which could cover each mucin domain^17^. Then we used multiple alignment method CLUSTALW to get an alignment^18^ (Fig. 1).

**Figure 2:**
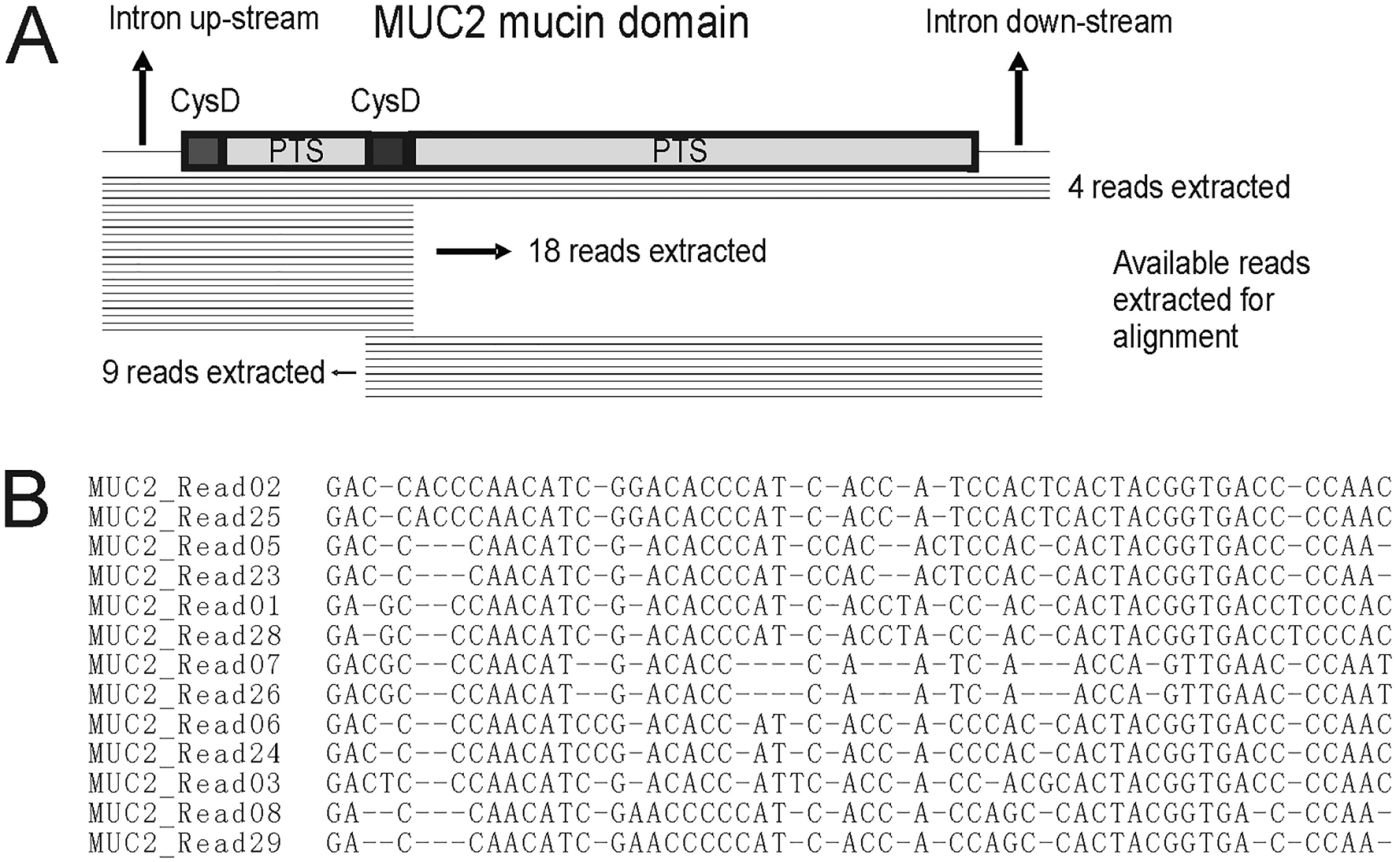
MUC2 mucin domain of HX1 and available reads for building MUC2 mucin domain as well as a small part of aligned reads. (**A**) Four reads could cover both the intron up-stream of mucin domain exon and the intron down-stream of mucin domain exon. 18 reads could cover the intron up-stream of mucin domain exon and the CysD domain between two PTS parts. Nine reads could cover the CysD domain between two PTS parts and the intron down-stream of mucin domain exon. (**B**) A small part of aligned reads which could present the alignment for constructing consensus sequence.

In the alignment, for each column we took the nucleotide (including insertion, i.e. A/T/C/G/-) which appears maximum number of times. Then we removed all the insertions and got the consensus sequence (Supplementary S5). Next we aligned back the consensus to all reads, and corrected the errors which were caused by several continuous insertions or same nucleotides (Fig. 1). For example, in a fragment with three ‘-’s and one ‘A’, some reads will give ‘- - - A’ and some will give ‘A - - -’. Then ‘A’ will be replaced by ‘-’ and later be removed in the consensus, and this will cause a missing of a nucleotide. Same principle, several continuous same nucleotides with an insertion might cause a redundant nucleotide in the consensus (Fig. 2B). This problem could cause a frame shift, and we managed to correct it according to the translation result. If we regard the error rate of SMRT as average 15–30%^20^, for each position, the average error rate is 0.15–0.3 to the power of the number of reads which could be aligned in this position.

### MUC2 mucin domain assembly result and TR structure

Altogether 31 reads could be used to build MUC2 mucin domain DNA sequence (Fig. 2A). In each position at least 7 reads could be aligned, thus for each nucleotide the average error rate is 0.15–0.3 to the power of 7 and the accuracy is 99.98–99.99%. The whole mucin domain exon of MUC2 in HX1 has 8994 bases.

**Figure 3:**
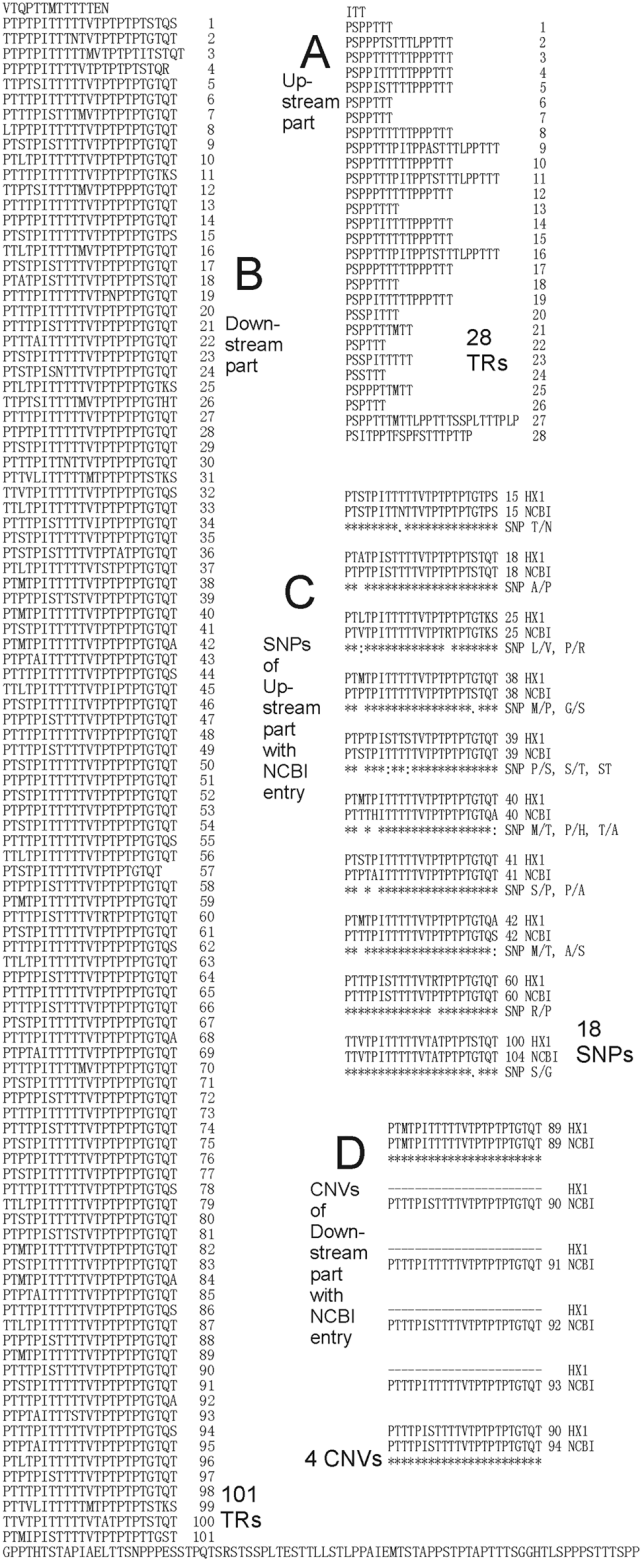
MUC2 mucin domain of HX1. (**A**) Up-stream part. Twenty-eight TRs could be found. (**B**) Down-stream part. One hundred and one TRs could be found. (**C**) SNP comparison of up-stream part with most complete NCBI entry. Eighteen SNPs could be found. (**D**) CNV comparison of down-stream part with most complete NCBI entry. Four more copy numbers could be found in NCBI entry.

The protein sequence of up-stream part of MUC2 mucin domain in HX1 has 2 CysD domains at the beginning and the end (Fig. 2A). They have 95 and 97 amino acids, respectively. Between them is the PTS TR up-stream part (Fig. 3A). The TR lengths vary a lot. We define each TR with a symbol “PS” at the start of it. Therefore, the PTS TR up-stream part has 28 TRs. The shortest TR has 7 amino acids and the longest TR has 26 amino acids. The protein sequences of PTS TR up-stream part as well as two CysD domains of MUC2 mucin domain in HX are exactly the same as those in NCBI (Nucleotide accession number NM_002457.4; Protein accession number NP_002448.4).

The PTS sequence after 2nd CysD domain of MUC2 mucin domain in HX1 is PTS TR down-stream part and it has 101 TRs (Fig. 3B). 98 TRs have 23 amino acids, respectively. 3rd TR has 24 amino acids. 4th TR has 22 amino acids. 57th TR has 21 amino acids.

The protein sequence of PTS TR down-stream part of MUC2 mucin domain in NCBI (Nucleotide accession number NM_002457.4; Protein accession number NP_002448.4) has 105 TRs. Comparing with that in HX1, it has 4 more TR CNVs directly after 89th repeat and 18 SNPs at 15th, 18th, 25th, 25th, 38th, 38th, 39th, 39th, 39th, 40th, 40th, 40th, 41st, 41st, 42nd, 42nd, 60th, and 104th repeat, respectively (Fig. 3C,D).

### MUC5AC mucin domain assembly result and TR structure

**Figure 4:**
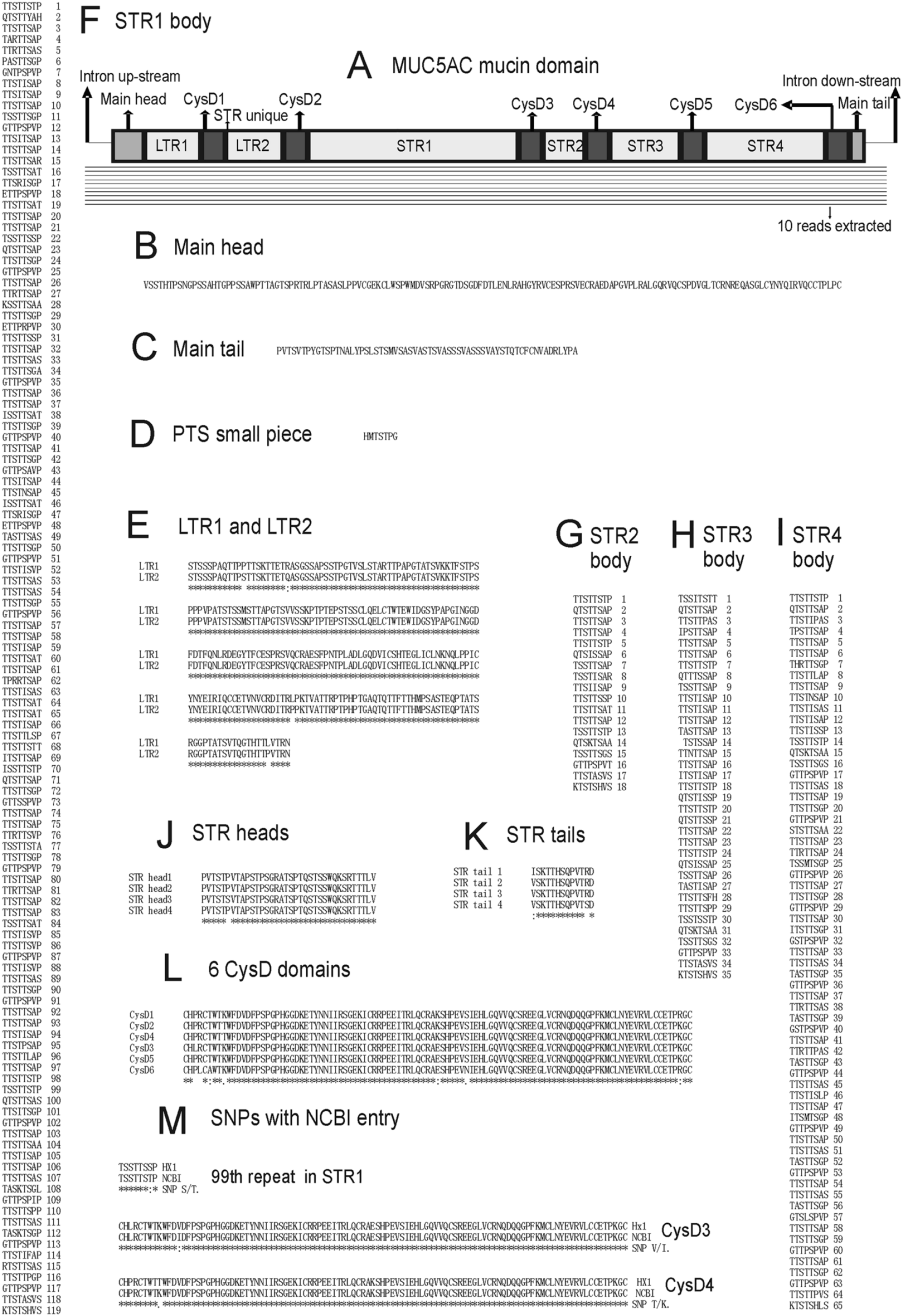
MUC5AC mucin domain of HX1. (**A**) Mucin domain and reads available for alignment. Ten reads could be used. (**B**) Main head. (**C**) Main tail. (**D**) Small PTS piece. (**E**) Alignment of LTR1 and LTR2. Four SNP sites could be found. (**F**) STR1 body. One hundred and nineteen TRs could be found. (**G**) STR2 body. Eighteen TRs could be found. (**H**) STR3 body. Thirty-five TRs could be found. (**I**) STR4 body. Sixty-five TRs could be found. (**J**) Alignment of four STR heads. One SNP site could be found. (**K**) Alignment of four STR tails. Two SNP sites could be found. (**L**) Alignment of six CysD domains. Seven SNP sites could be found. (**M**) SNP comparison with most complete NCBI entry. One SNP could be found in 99th repeat in STR1, one SNP could be found in CysD3, and one SNP could be found in CysD4.

Altogether 10 reads could be used to build MUC5AC mucin domain DNA sequence (Fig. 4A). In each position at least 6 reads could be aligned, thus for each nucleotide the average error rate is 0.15–0.3 to the power 6 and the accuracy is 99.93–99.99%. The whole mucin domain exon of MUC5AC in HX1 has 10,371 bases.

The protein sequence of MUC5AC mucin domain in HX1 has one main head, one main tail, 6 CysD domains, 2 Long Tandem Repeat (LTR) groups, 4 Short Tandem Repeat (STR) groups and 1 unique short piece (Fig. 4A). The main head is composed of a PTS domain of 45 amino acids long and a CysD like domain of 99 amino acids long (Fig. 4B). The main tail is composed of a PTS domain of 50 amino acids long and a short piece of 12 amino acids long (Fig. 4C). For all 6 CysD domains, each has 105 amino acids and locates after each LTR/STR group (Fig. 4L). For 2 LTR groups, each is composed of one PTS domain of 95 amino acids long, one CysD like domain of 101 amino acids long, and one PTS domain of 65 amino acids long (Fig. 4E). Other than one CysD domain, there is one small PTS piece of 7 amino acids long between the 2 LTR groups (Fig. 4D). For 4 STR groups, each has one PTS head of 36 amino acids long and one PTS tail of 13 amino acids long (Fig. 4J,K). 1st, 2nd, 3rd and 4th STR group have 119, 18, 35, and 65 STRs, respectively (Fig. 4F–H,J). Each repeat has 8 amino acids except that 14th repeat of 3rd STR group has only 7 amino acids (Fig. 4F–I). As the delimiters of LTR/STR groups, 6 CysD domains are nearly identical (Fig. 4L).

The protein sequence of MUC5AC mucin domain in NCBI (Nucleotide accession number NM_001304359.1; Protein accession number NP_001291288.1) has same length and TR structure as that in HX1. There are only 3 SNPs. One is in 99th repeat in 1st STR group; another two are in 3rd and 4th CysD, respectively (Fig. 4M).

### MUC5B mucin domain assembly result and TR structure

**Figure 5:**
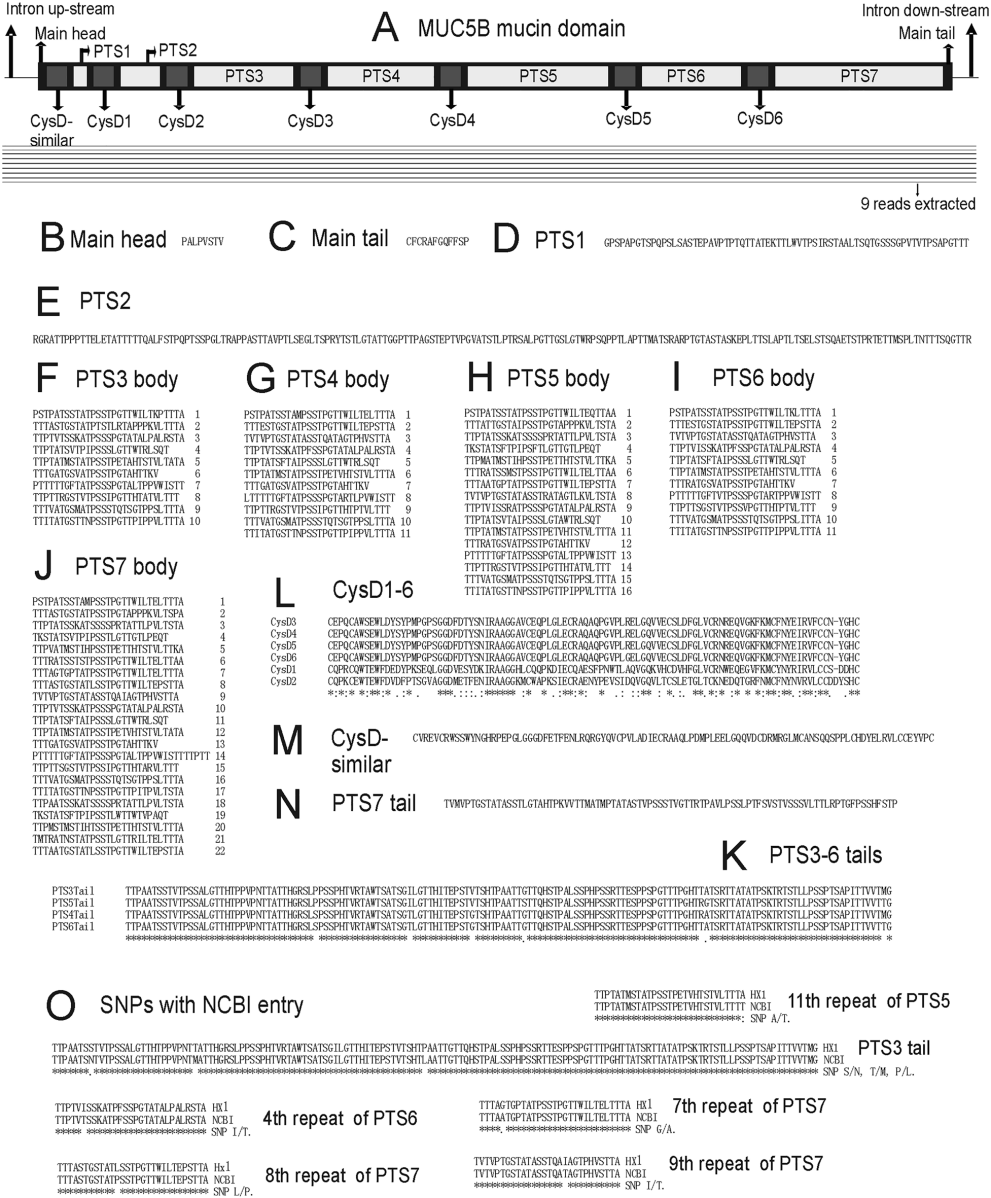
MUC5B mucin domain of HX1. (**A**) Mucin domain and reads available for alignment. Nine reads could be used. (**B**) Main head. (**C**) Main tail. (**D**) PTS1. (**E**) PTS2. (**F**) PTS3 body. Ten TRs could be found. (**G**) PTS4 body. Eleven TRs could be found. (**H**) PTS5 body. Six-teen TRs could be found. (**I**) PTS6 body. Eleven TRs could be found. (**J**) PTS7 body. Twenty-two TRs could be found. (**K**) Alignment of tails of PTS3, PTS4, PTS5 and PTS6. Seven SNP sites could be found. (**L**) Alignment of six CysD domains. Fifty-nine SNP sites could be found. (**M**) CysD-similar domain. (**N**) PTS7 tail. (**O**) SNP comparison with most complete NCBI entry. Three SNPs could be found in PTS3 tail, one SNP could be found in 11th repeat of PTS5, one SNP could be found in 4th repeat of PTS6, one SNP could be found in 7th repeat of PTS7, one SNP could be found in 8th repeat of PTS7 and one SNP could be found in 9th repeat of PTS7.

Altogether 9 reads could be used to build MUC5B mucin domain DNA sequence (Fig. 5A). In each position at least 5 reads could be aligned, thus for each nucleotide the average error rate is 0.15–0.3 to the power 5 and the accuracy is 99.76–99.99%. The whole mucin domain exon of MUC5B in HX1 has 10,893 bases.

The protein sequence of MUC5B mucin domain in HX1 has one main head, one main tail, 1 CysD-similar domain, 6 CysD domains, and 7 PTS domains (Fig. 5A). The main head is composed of a small piece of 8 amino acids long (Fig. 5B). The main tail is composed of a small piece of 12 amino acids long (Fig. 5C). The CysD-similar domain has 100 amino acids (Fig. 5M). 2nd CysD domain has 102 amino acids (Fig. 5L). For other 5 CysD domains, each has 101 amino acids (Fig. 5L). For all 7 CysD and CysD-similar domains, each locates before one PTS domain (Fig. 5A). The first 2 PTS domains have no repeats, but a long piece of 70 and 180 amino acids, respectively (Fig. 5D,E). Each of the last 5 PTS domains has several STRs and one PTS tail (Fig. 5F–K,N). The number of STRs of the bodies of 3rd, 4th, 5th, 6th, and 7th PTS domain are 10, 11, 16, 11, and 22, respectively (Fig. 5F–J). For all the STRs in the bodies of last 5 PTS domains, 5 have 24 amino acids, respectively; 8 have 26 amino acids, respectively; 8 have 28 amino acids, respectively; 48 have 29 amino acids, respectively; one has 34 amino acids (Fig. 5F–J). The PTS tails of 3rd, 4th, 5th and 6th PTS domains are homologous and they all have 147 amino acids, respectively (Fig. 5K). The PTS tail of 7th PTS domain has 87 amino acids (Fig. 5N). As the delimiters of 7 PTS domains, 2nd, 3rd, 4th, 5th, and 6th CysD domains are nearly identical (Fig. 5L).

The protein sequence of MUC5B mucin domain in NCBI (Nucleotide accession number NM_002458.2; Protein accession number NP_002449.2) has same length and TR structure as that in HX1. There are only 7 SNPs. Three are in PTS tail of 3rd PTS domain, one is in 11th repeat of 5th PTS domain, one is in 4th repeat of 6th PTS domain, and three are in 7th, 8th and 9th repeats of 7th PTS domain, respectively (Fig. 5O).

### MUC6 mucin domain assembly result and TR structure

Altogether only 3 reads could be used to build MUC5B mucin domain DNA sequence (Fig. 5A). Therefore, it is impossible to get the exactly correct nucleotide in each position. However, due to the TR structure, the number of TRs and the lengths of each TR could be obtained. The MUC6 Refseq has all the non-TR part of mucin domain, thus we can use this as the template to get the whole mucin domain exon of MUC6 in HX1 which has been identified to have 13,470 bases by us.

**Figure 6:**
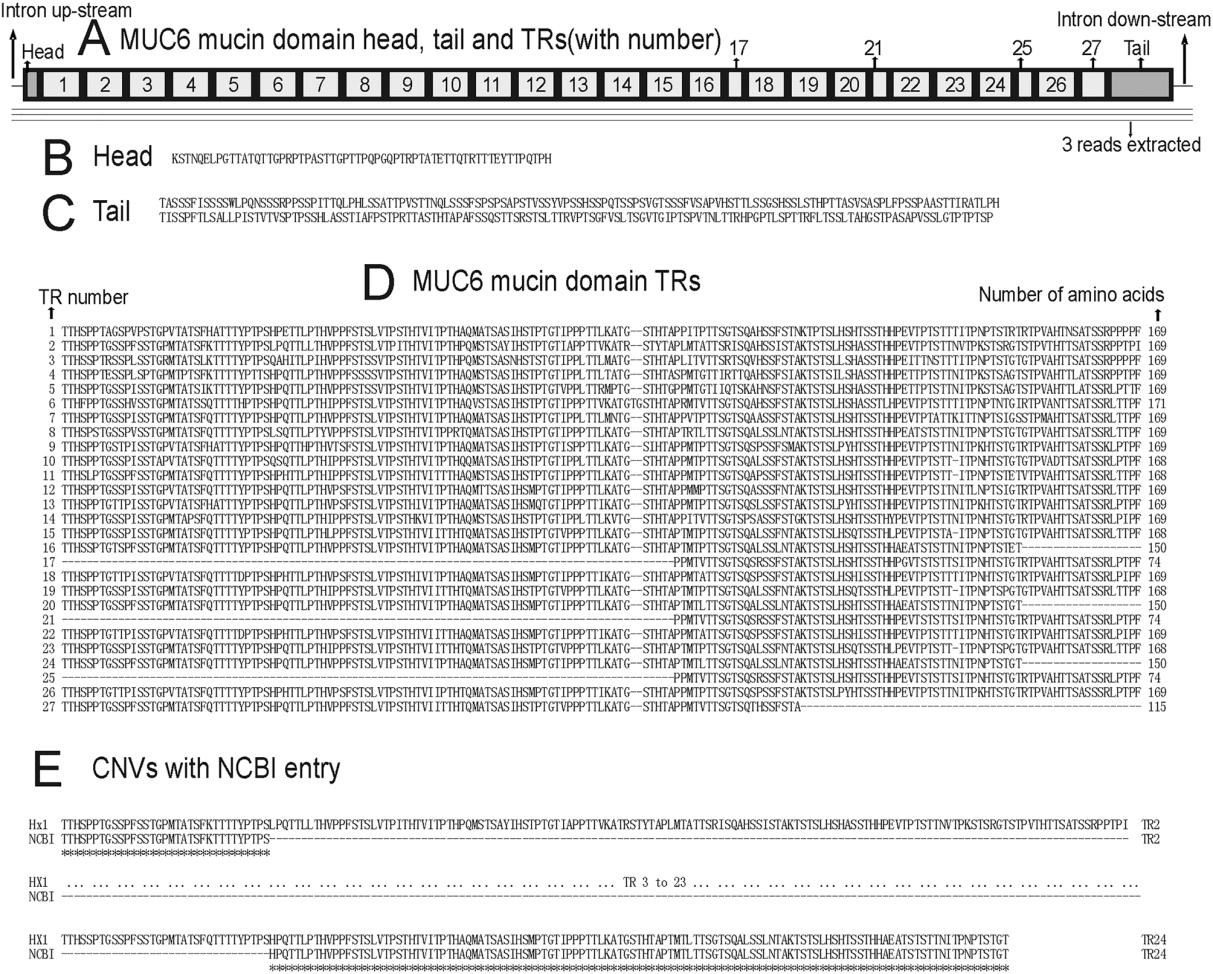
MUC6 mucin domain of HX1. (**A**) Mucin domain and reads available for alignment. Three reads could be used. (**B**) Head. (**C**) Tail. (**D**) Twenty-seven TRs. (**E**) CNV comparison with most complete NCBI entry. Twenty-one more copy numbers could be found in HX1.

The protein sequence of MUC6 mucin domain inHX1 has one head, one tail and 27 TRs (Fig. 6A). The head has 60 amino acids and the tail has 265 amino acids (Fig. 6B,C). 27 TRs could be found between the head and the tail. 1st, 2nd, 3rd, 4th, 5th, 7th, 8th, 9th, 12th, 13th, 14th, 18th, 22nd, and 26th TRs have 169 amino acids, respectively. This number is most of the case among all TRs, thus we call this type of TRs “typical TR”. 6th TR has 171 amino acids, and there is a “TG” insertion comparing with the typical TR. 10th, 11th, 15th, 19th, and 23rd TRs have 168 amino acids, respectively, and there is a deletion comparing with the typical TR. 16th, 20th, and 24th TRs have 150 amino acids, respectively, and they are first 150 amino acids of the typical TR. 17th, 21st, and 25th TRs have 74 amino acids, respectively, and they are last 74 amino acids of the typical TR. 27th TR has 115 amino acids, and it is the first 115 amino acids of the typical TR (Fig. 6D).

The protein sequence of MUC6 mucin domain in NCBI (Nucleotide accession number NM_005961.2; Protein accession number NP_005952.2) only has the head, 1st TR, first 33 amino acids of 2nd TR, last 117 amino acids of 24th TR, 25th TR, 26th TR, 27th TR, and the tail (Fig. 6E). Since we cannot be sure the exact nucleotide in each position due to only 3 reads available, we cannot say SNP information.

### Estimation of number of TRs in down-stream part of mucin domain of MUC2 for another individual

**Figure 7:**
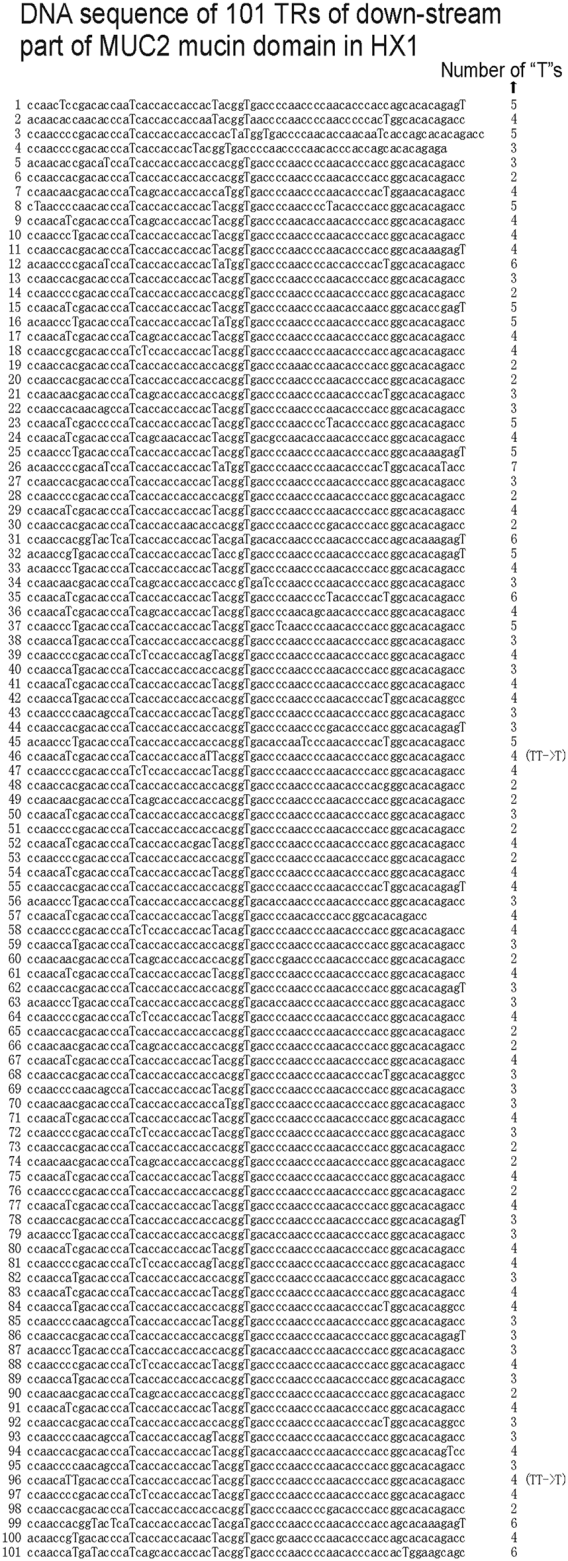
DNA sequence of down-stream part TRs of MUC2 mucin domain in HX1. Other than 2 “TT” SNPs at 17th and 96th TR, no continuous “T”s could be found in this part. The number difference of “T”s between two MUC2 mucin domain DNA sequences could be used to estimate the number difference of TRs between these two domains.

In the result from the pipeline, all frameshifts are caused by several continuous same nucleotides. In HX1, in the DNA sequence of TRs in down-stream part of mucin domain of MUC2 mucin domain, no continuous multiple “T”s could be found other than two SNPs at 46th and 96th TR, respectively, which cause two continuous “T”s (Fig. 7). Therefore, the number of “T”s in the TR part from pipeline consensus could be used to estimate the number of repeats without arranging each frameshift (Table 2).

**Table 2:** Nucleotide statistics of MUC2 mucin domain down-stream TR part.

A	T	C	G
**Number of nucleotides in pipeline consensus of HX1**			
2304	364	3623	1100
**Number of nucleotides in real HX1 after adjustment**			
2228	364	3640	1101

We can see that the number of single letter “T” keeps same after adjustment. Therefore that number could be used to estimate the number of tandem repeats.

**Figure 8:**
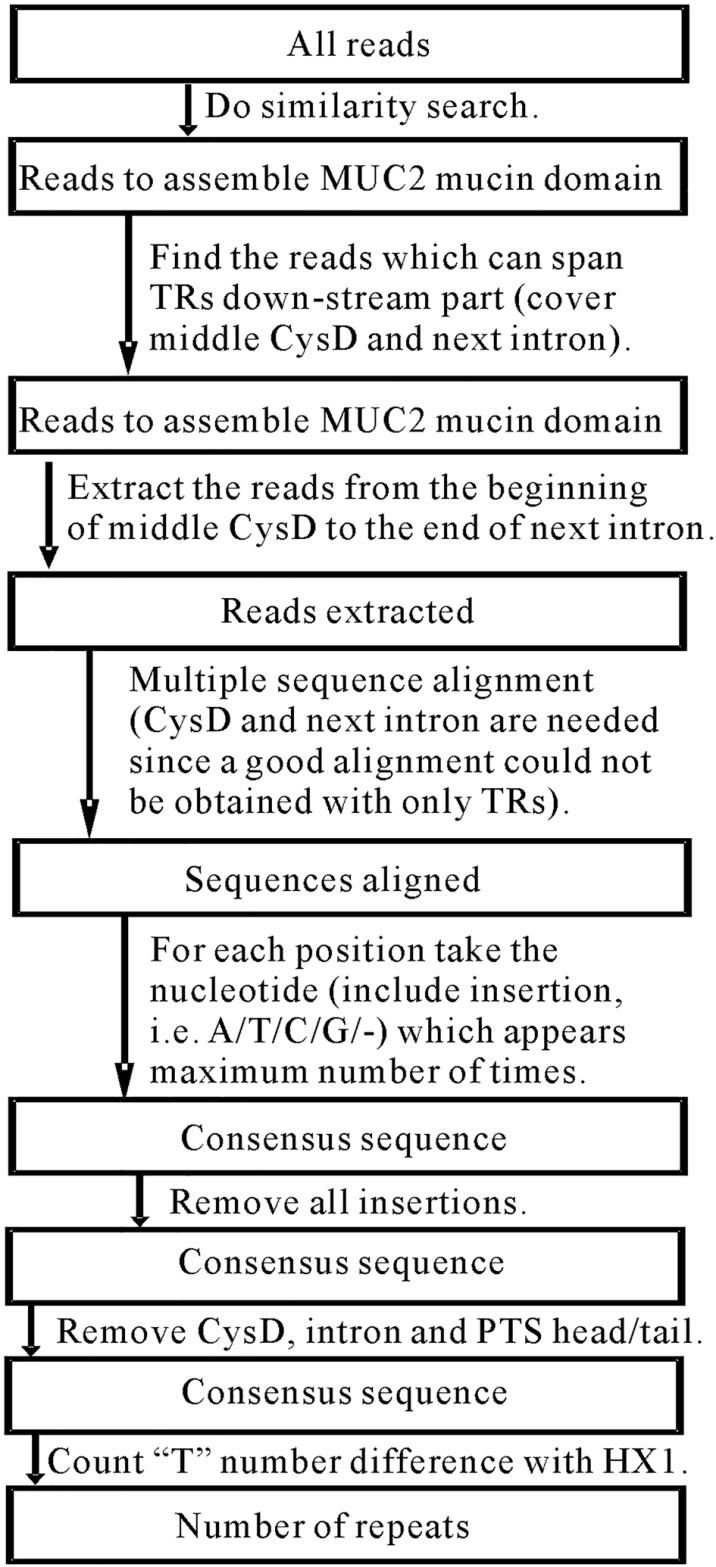
Steps to estimate number of repeats in down-stream part of MUC2 mucin domain. Step1: use all reads to do similarity search and get reads which could be used to assemble MUC2 mucin domain. Step2: Find reads which can cover middle CysD and intron down-stream. Step3: Extract those reads from the beginning of middle CysD to the end of intron down-stream. Step4: Do multiple sequence alignment. Step5: Get consensus sequence by counting maximum number of nucleotide in each position. Step6: Modify consensus sequence by removing all insertions. Step7: Remove non-TR parts (CysD, intron, PTS head/tail). Step 8: Count “T” number difference with HX1. Since the number of repeats of HX1 is 101, we can get the exact number.

In down-stream part of mucin domain of MUC2 for HX1, the number of “T”s keeps same after adjustment. For each repeat, in most cases “T” appears 2 or 3 or 4 or 5 times, in some less number of cases “T” appears 6 times, and only once “T” appears 7 times. Therefore, for any individual, after checking “T” number we could get repeat number roughly by comparing with that in HX1. In HX1, the down-stream part has 101 TRs and 364 “T”s. 46th and 96th TR have one “TT”, respectively. Since in common repeats we cannot find “TT”, we regard such cases as SNPs and count two continuous “T”s as one. Therefore “T” number of 362 shall correspond to repeat number of 101, and on average each repeat has 3.58 “T”s (Fig. 7). If we divide “T” number difference by 3.58, we can roughly get repeat number difference. For instance, DNA sequence of most complete MUC2 protein in NCBI (accession number NP_002448.4) has 13 more “T”s and 4 more repeats. However, some SNPs (T/A, T/C, or T/G) might affect T number difference. Anyway a roughly estimation of repeat number could be obtained in this way (Fig. 8).

## Discussion

The DNA sequences of mucin domains of human gel-forming mucin genes MUC2, MUC5AC, MUC5B and MUC6 consist of long tandem repeats that are difficult to read and measure. This lack of essential sequence information has left these genes mysterious with huge amount of gaps, which has hindered people for understanding gene function in health and disease^21^. To fill in these gaps, it is essential to use the correct way of assembly with SMRT long sequence reads which could cover the mucin domains.

**Figure 9:**
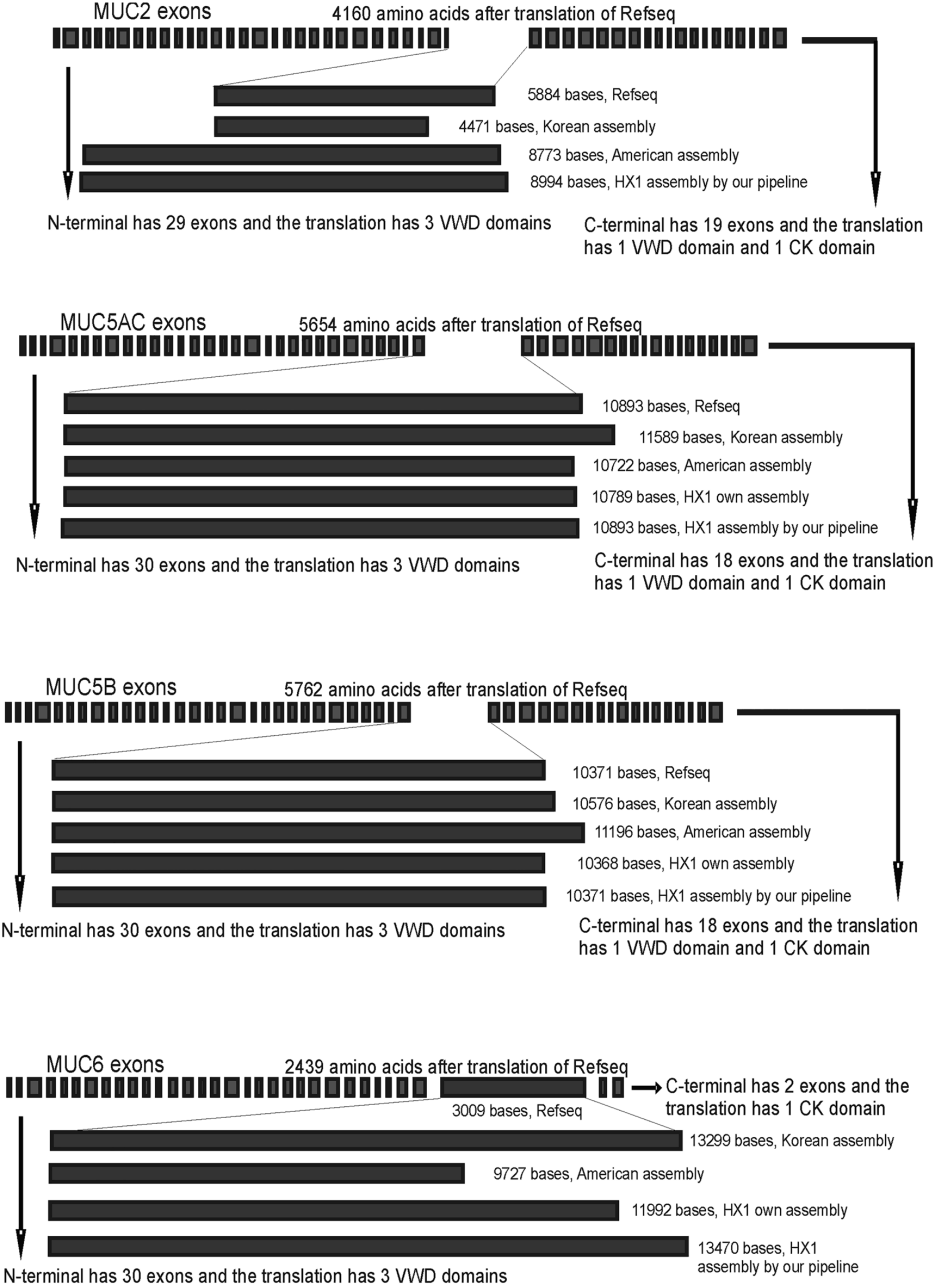
Exon structure of gel-forming mucin MUC2, MUC5AC, MUC5B and MUC6. For each gene, mucin domain is one big exon and takes most proportion of the coding sequence. The length of each exon is in scale here. N-terminal and C-terminal exons are taken from Refseq. The translated amino acids numbers of Refseq for MUC2, MUC5AC, MUC5B and MUC6 are 4160, 5654, 5703 and 2439, respectively. Note that the length of DNA of MUC2 mucin domain in Refseq is 5884 bases, and it is needed to remove one base to get the correct frame after combining with N-terminal and C-terminal coding sequences.

In HX1 own assembly, mucin domains of MUC2, MUC5AC, MUC5B and MUC6 could not be found in one cluster^13^. MUC2 mucin domain could not be found at all. MUC5AC mucin domain and MUC5B mucin domain could be found in one contig with 10,368 bases and 10,789 bases, respectively. MUC6 mucin domain could be found in another contig with 11,992 bases^13^. However, with our pipeline, we got the DNA lengths of mucin domains of MUC2, MUC5AC, MUC5B and MUC6 at 8994 bases, 10,371 bases (same as Refseq), 10,893 bases (same as Refseq), and 13,470 bases, respectively (Table 1). Moreover, for each of the gel-forming mucins, mucin domain exon takes big proportion of whole coding sequence part, and the amino acids translated are highly glycosylated to fulfill protection function, thus make this exon much more important (Supplementary S6–S9). Therefore, the accuracy of deciphering mucin domain exons is of great significance, and our result could provide more precise information than HX1 own assembly (Fig. 9).

Through the method we developed, we managed to generate full and reliable sequences which have verified to be extremely hard to obtain in the past^5,6,11,21^. With this we could reveal the exact number of TRs in gel-forming mucin domains in a rather quick way. Therefore, people could try to seek the link between mucin domain TR numbers and the corresponding human traits in large populations, and this will be helpful to study genetic associations with many mucus related diseases such as pancreatic cancer, breast cancer, lung cancer, egg groove cancer, colon cancer, asthma, bronchitis, chronic obstructive pulmonary disease, pulmonary cyst fibrosis, gastric ulcer, edema, etc.

## Supplementary Information

Supplementary Information.

## Data Availability

All data are downloaded from NCBI website. Whole chromosome 11 of NCBI human genome Refseq (Accession number NC_000011): https://www.ncbi.nlm.nih.gov/nuccore/NC_000011.10. Chromosome 11 of human genome assembly of a Korean individual (Accession number GCA_001712695.1): https://www.ncbi.nlm.nih.gov/assembly/GCA_001712695.1. Whole human genome assembly of an American individual (Accession number GCA_001013985.1): https://www.ncbi.nlm.nih.gov/assembly/GCA_001013985.1. Whole human genome SRA data of a Chinese individual (HX1, Accession number GCA_001708065.2): https://www.ncbi.nlm.nih.gov/bioproject/PRJNA301527/.

## Abbreviations

TR(s): Tandem repteat(s)
LTR: Long tandem repeat
STR: Short dandem repeat
SMRT: Single molecule real-time
SNP(s): Single nucleotide polymorphism(s)
CNV(s): Copy number viaration(s)
PTS: Proline, threonine and serine
VWD: Von Willebrand D
CK: Cysteine knot
HX1: A Chinese individual
Chr: Chromosome
NCBI: National Center for Biotechnology Information
Refseq: NCBI reference sequence
SRA: Sequence read archive
Gbp: Gigabases
