# Supplementary material for: Tandem repeats structure of gel-forming mucin domains could be revealed by SMRT sequencing data
Source: Sci Rep. 2022 Nov 30;12:20652. doi: 10.1038/s41598-022-25262-7 (PMC9712336; doi:10.1038/s41598-022-25262-7)
Supplement: Supplementary file 1 — Supplementary Information. [file 41598_2022_25262_MOESM1_ESM.pdf]

## Supplementary information to

# Tandem repeats structure of gel-forming mucin domains could be revealed by SMRT sequencing data

Tiange Lang

**Scripts S1 – S5**

1. Python script for converting SRA data to fasta sequence data.
2. Perl script for executing the python script with specific parameters as well as dealing with all reads one by one automatically.
3. Perl script for quality control.
4. Perl script for length control.
5. Perl script for making consensus sequence.

**Sequences S6 – S9**

6. Refseq genome DNA sequence of human MUC2. Exons are uppercase letter with underline (5' and 3' untranslated regions are not included). Mucin exon is the 30th exon. Note that adjustment of mucin exon is needed if you want to get one frame translation.
7. Refseq genome DNA sequence of human MUC5AC. Exons are uppercase letter with underline (5' and 3' untranslated regions are not included). Mucin exon is the 31st exon.
8. Refseq genome DNA sequence of human MUC5B. Exons are uppercase letter with underline (5' and 3' untranslated regions are not included). Mucin exon is the 31st exon.
9. Refseq genome DNA sequence of human MUC6. Exons are uppercase letter with underline (5' and 3' untranslated regions are not included). Mucin exon is the 31st exon.

# Script S1

:::::::::::::
bash5tools.py: This program was used to convert SRA data to fasta sequence data.
This program was downloaded from Github.
:::::::::::::

```
#!/usr/bin/env python
################################################################################
##
# Copyright (c) 2011-2013, Pacific Biosciences of California, Inc.
#
# All rights reserved.
#
# Redistribution and use in source and binary forms, with or without
# modification, are permitted provided that the following conditions are met:
# * Redistributions of source code must retain the above copyright
#     notice, this list of conditions and the following disclaimer.
# * Redistributions in binary form must reproduce the above copyright
#     notice, this list of conditions and the following disclaimer in the
#     documentation and/or other materials provided with the distribution.
# * Neither the name of Pacific Biosciences nor the names of its
#     contributors may be used to endorse or promote products derived from
#     this software without specific prior written permission.
#
# NO EXPRESS OR IMPLIED LICENSES TO ANY PARTY'S PATENT RIGHTS ARE GRANTED BY
# THIS LICENSE.    THIS SOFTWARE IS PROVIDED BY PACIFIC BIOSCIENCES AND ITS
# CONTRIBUTORS "AS IS" AND ANY EXPRESS OR IMPLIED WARRANTIES, INCLUDING, BUT NOT
# LIMITED TO, THE IMPLIED WARRANTIES OF MERCHANTABILITY AND FITNESS FOR A
# PARTICULAR PURPOSE ARE DISCLAIMED. IN NO EVENT SHALL PACIFIC BIOSCIENCES OR
# ITS CONTRIBUTORS BE LIABLE FOR ANY DIRECT, INDIRECT, INCIDENTAL, SPECIAL,
# EXEMPLARY, OR CONSEQUENTIAL DAMAGES (INCLUDING, BUT NOT LIMITED TO,
# PROCUREMENT OF SUBSTITUTE GOODS OR SERVICES; LOSS OF USE, DATA, OR PROFITS; OR
# BUSINESS INTERRUPTION) HOWEVER CAUSED AND ON ANY THEORY OF LIABILITY, WHETHER
# IN CONTRACT, STRICT LIABILITY, OR TORT (INCLUDING NEGLIGENCE OR OTHERWISE)
# ARISING IN ANY WAY OUT OF THE USE OF THIS SOFTWARE, EVEN IF ADVISED OF THE
# POSSIBILITY OF SUCH DAMAGE.
################################################################################
##
import os, os.path, sys, argparse, logging

from pbcore.util.ToolRunner import PBToolRunner
from pbcore.io import (BasH5Reader,
                       FastaWriter,
                       FastqWriter)
```

```
from pbh5tools._version import __version__

def _fileType(arg):
    """
    Canonicalize the given filetype argument
    """
    if    arg in ["fa", "fasta", "FASTA"]: return "fasta"
    elif arg in ["fq", "fastq", "FASTQ"]: return "fastq"
    else: raise ValueError("Unsupported output file format")


class FastaEmitter(object):
    def __init__(self, filename):
        self.writer = FastaWriter(filename)

    def emit(self, zmwRead):
        self.writer.writeRecord(zmwRead.readName,
                                zmwRead.basecalls())

class FastqEmitter(object):
    def __init__(self, filename):
        self.writer = FastqWriter(filename)

    def emit(self, zmwRead):
        self.writer.writeRecord(zmwRead.readName,
                                zmwRead.basecalls(),
                                zmwRead.QualityValue())

class BasH5ToolsRunner(PBToolRunner):

    def __init__(self):
        desc = "Tool for extracting data from .bas.h5 files"
        super(BasH5ToolsRunner, self).__init__(desc)

        self.parser.add_argument(
            "inFile", metavar="input.bas.h5",
            help="input .bas.h5 filename")
        self.parser.add_argument(
            "--outFilePrefix", dest="outFilePrefix", default=None,
            help="output filename prefix [%(default)s]")
        self.parser.add_argument(
            "--readType", dest="readType", default="",
            choices=["ccs", "subreads", "unrolled"],
```

```
            help="read type (ccs, subreads, or unrolled) [%(default)s]")
        self.parser.add_argument(
            "--outType", dest="outType", default="fasta", type=_fileType,
            help="output file type (fasta, fastq) [%(default)s]")

        groupFilt = self.parser.add_argument_group("Read filtering arguments")
        groupFilt.add_argument(
            "--minLength", type=int, dest="minLength", default=0,
            help="min read length [%(default)s]")
        groupFilt.add_argument(
            "--minReadScore", type=float, dest="minReadScore", default=0,
            help="min read score, valid only with --readType={unrolled,subreads}
[%(default)s]")
        groupFilt.add_argument(
            "--minPasses", type=int, dest="minPasses", default=0,
            help="min number of CCS passes, valid only with --readType=ccs [%(default)s]")

    def getVersion(self):
        return __version__

    def validateArgs(self):
        if not os.path.isfile(self.args.inFile):
            self.parser.error("File %s does not exist!" % self.args.inFile)
        if self.args.minReadScore > 1.0 or self.args.minReadScore < 0.0:
            self.parser.error("Minimum read score needs to be > 0.0 and < 1.0")

    def zmwReads(self, inBasH5, readType):
        """
        Extract all reads of the appropriate read type
        """
        for zmw in inBasH5:
            if readType == "ccs":
                r = zmw.ccsRead
                if r: yield r
            elif readType == "unrolled":
                yield zmw.read()
            else:
                for r in zmw.subreads:
                    yield r

    def run(self):
        inBasH5 = BasH5Reader(self.args.inFile)

        if not inBasH5.hasConsensusBasecalls and self.args.readType == "ccs":
```

```
            print "Input file %s contains no CCS reads." % self.args.inFile
            sys.exit(-1)

        if not inBasH5.hasRawBasecalls and self.args.readType in ["unrolled", "subreads"]:
            print "Input file %s contains no %s reads" % (self.args.inFile,
                                                           self.args.readType)
            sys.exit(-1)

        movieName = inBasH5.movieName
        outFilePrefix = self.args.outFilePrefix or movieName
        outFilename = "%s.%s" % (outFilePrefix, self.args.outType)

        if self.args.outType == "fasta":
            sink = FastaEmitter(outFilename)
        elif self.args.outType == "fastq":
            sink = FastqEmitter(outFilename)

        if self.args.readType == '':
            # choose based on file.
            if inBasH5.hasRawBasecalls:
                readType = 'subreads'
            elif inBasH5.hasConsensusBasecalls:
                readType = 'ccs'
            else:
                print "Input bas.h5 file has neither CCS nor subread data"
                sys.exit(-1)
        else:
            readType = self.args.readType

        for zmwRead in self.zmwReads(inBasH5, readType):
            zmw = zmwRead.zmw
            #
            # Emit read if filters pass
            #
            if ((readType != "ccs" or zmw.numPasses >= self.args.minPasses)        and
                (readType == "ccs" or zmw.readScore >= self.args.minReadScore) and
                (len(zmwRead) >= self.args.minLength)):

                sink.emit(zmwRead)

if __name__ == "__main__":
    sys.exit(BasH5ToolsRunner().start())
```

# Script S2

:::::::::::::

python_h5.pl: This program was used to execute the python program which could convert SRA data to fasta sequence data with specific parameters as well as deal with all reads one by one automatically.

This program is made by Tiange Lang.

:::::::::::::

```
#!/usr/bin/perl

@files=qx{ls};
chomp $files[1];
$h=$files[1];
$h=~s/\..*//;
system ("python bash5tools.py $h.bas.h5 --minLength 5000");
```

# Script S3

:::::::::::::

fastqTrim_Quality.pl: This program was used to remove the nucleotides which have Phred score lower than a specific value.

This program is made by Tiange Lang.

:::::::::::::

```
#!/usr/bin/env perl

use strict; use warnings; use Getopt::Long; use File::Spec;

my $usage = "
$0 input_files [-p|probcutoff 0.05] [-h|phredcutoff 13] [-b|bwa] [-d|directory path] [-sanger
-solexa -illumina] [-454]\n
-p|probcutoff  probability value (between 0 and 1) at which base-calling error is considered too
high (default; p = 0.05) *or*
-h|phredcutoff    Phred score (between 0 and 40) at which base-calling error is considered too
high
-b|bwa                  use BWA trimming algorithm
-d|directory        path to directory where output files are saved
-sanger                 Sanger format (bypasses automatic format detection)
-solexa                 Solexa format (bypasses automatic format detection)
-illumina              Illumina format (bypasses automatic format detection)
-454                      set flag if trimming Roche 454 data (experimental feature)
\n";
if( !$ARGV[0] ){ die "$usage"; }
my $prob_cutoff;
my $phrd_cutoff;
my $ascii_cutoff;
my $automatic_detection_lines = 10000;
my $sanger;
my $solexa;
my $illumina;
my $format;
my $user_defined;
my $bwa;
my $directory;
my $roche;
my $poor_quality_char = "B";
GetOptions(
        "p|probcutoff=f"    => \$prob_cutoff,
        "h|phredcutoff=f" => \$phrd_cutoff,
        "b|bwa"                     => \$bwa,
        "d|directory=s"      => \$directory,
```

```
	"sanger"		=> \$sanger,
	"solexa"		=> \$solexa,
	"illumina"		=> \$illumina,
	"454"			=> \$roche
);
if( ($sanger && $solexa) || ($sanger && $illumina) || ($solexa && $illumina) ){
	die "error: please select only one of -sanger, -solexa or -illumina\n";
}
if( $sanger || $solexa || $illumina ){
	$user_defined = 1;
}
if( $sanger ){
	$format = "sanger";
}elsif( $solexa ){
	$format = "solexa";
}elsif( $illumina ){
	$format = "illumina";
}
if( $roche ){
	$format = "sanger";
}
my @files = @ARGV;
if( !$files[0] ){ die "$usage"; }
if( !defined( $prob_cutoff ) && !defined( $phrd_cutoff ) ){
	$prob_cutoff = 0.05;
	print STDOUT "Info: Using default quality cutoff of P = $prob_cutoff (change with -p or -h
flag)\n";
}elsif( defined( $prob_cutoff ) && defined( $phrd_cutoff ) ){
	die "Error: Please enter either a probability or a Phred quality cutoff value, not both";
}elsif( defined( $prob_cutoff ) && ( $prob_cutoff < 0 || $prob_cutoff > 1 ) ){
	die "Error: P quality cutoff must be between 0 and 1";
}elsif( defined( $phrd_cutoff ) && $phrd_cutoff < 0 ){
	die "Error: Phred quality cutoff must be greater than or equal to 0";
}
if( !`which R 2> err.log` ){
	print STDERR "Warning: Subsidiary program R not found. Histogram will not be
produced.\n";
}
`rm err.log`;
foreach my $input_file ( @files ){
	open( INPUT, "<$input_file" ) or die "Error: Failure opening $input_file for reading: $!\n";
	my @filepath = split( /\//, $input_file );
	my $filename = $filepath[$#filepath];
	if( !$user_defined ){
```

```
		$format = "";
	}
	if( !$format ){

		$format = &get_format(*INPUT, $automatic_detection_lines);
		if( !$format ){
			die "Error: File format cannot be determined\n";
		}
	}
	my %dict_q_to_Q;
	%dict_q_to_Q=&q_to_Q();
	if( $format eq "sanger" ){
		$poor_quality_char = "!";
	}elsif( $format eq "solexa" ){
		$poor_quality_char = ";";
	}elsif( $format eq "illumina" ){
		$poor_quality_char = "@";
	}
	if( $roche ){
		print STDOUT "User defined format: Roche 454, Sanger FASTQ format\n";
	}elsif( $format eq "sanger" ){
		if( $user_defined ){
			print STDOUT "User defined format: Sanger FASTQ format\n";
		}else{
			print STDOUT "Automatic format detection: Sanger FASTQ format\n";
		}
	}elsif( $format eq "solexa" ){
		if( $user_defined ){
			print STDOUT "User defined format: Solexa FASTQ format, Illumina pipeline 1.2 or
less\n";
		}else{
			print STDOUT "Automatic format detection: Solexa FASTQ format, Illumina
pipeline 1.2 or less\n";
		}
	}elsif( $format eq "illumina" ){
		if( $user_defined ){
			print STDOUT "User defined format: Illumina FASTQ format, Illumina pipeline
1.3+\n";
		}else{
			print STDOUT "Automatic format detection: Illumina FASTQ format, Illumina
pipeline 1.3+\n";
		}
	}
	if( defined( $phrd_cutoff ) ){
```

```
	$ascii_cutoff = &Q_to_q( $phrd_cutoff );
	$prob_cutoff = sprintf("%.5f", &Q_to_p( $phrd_cutoff ));
}else{
	$ascii_cutoff = &Q_to_q( &p_to_Q( $prob_cutoff ) );
}
my $threshold = 0;
if( $bwa ){

	if( defined( $phrd_cutoff ) ){
		$threshold = $phrd_cutoff;
	}else{
		$threshold = &p_to_Q( $prob_cutoff );
	}
}
my $output_file;
if ( $directory ){
	# remove any trailing '/'
	$directory =~ s/\/\z//;
	my $file_name = $filename . ".trimmed";
	$output_file = File::Spec->catpath( undef, $directory, $file_name );
}else{
	$output_file = $filename . ".trimmed";
}

if( -e $output_file ){
	die "Error: Output file $output_file already exists: $!\n";
}
open( OUTPUT, ">$output_file" )
			or die "Error: Failure opening $output_file for writing: $!\n";
my @segment_hist;
my %hash=();
my $segment_sum	= 0;
my $segment_count = 0;
my $original_length;
my $seq_count = 0;
while( <INPUT> ){
	my $ID1 = $_;
	if( substr( $ID1, 0 , 1) ne "@" ){
		die "Error: Input file not in correct FASTQ format at seq ID $ID1\n";
	}
	chomp( my $seq_string = <INPUT> );
	my $ID2 = <INPUT>;
	if( substr( $ID2, 0 , 1) ne "+" ){
		die "Error: Input file not in correct FASTQ format at qual ID $ID2\n";
```

```
}
chomp( my $quality_string = <INPUT> );
$original_length    = length $seq_string;
my $cutoff_hit             =    0;
my $best_start_index =    0;
my $best_length           =    0;
my $current_start        =    0;
my $bad_first               =    0;
if( $bwa ){
        my @qual = split(//, $quality_string );
        for( my $i = 0; $i < scalar @qual; $i++ ){

                $qual[$i] = $dict_q_to_Q{$qual[$i]};
        }
        if( !$qual[0] ){
                $bad_first = 1;
                $best_length = 0;

        }elsif( $qual[0] < $threshold ){
                $bad_first = 1;
                $best_length = &bwa_trim( $threshold, \@qual );

        }else{
                $best_length = &bwa_trim( $threshold, \@qual );
        }
}else{
        for( my $i = 0; $i < $original_length; $i++ ){
                if( substr($quality_string, $i, 1) le $ascii_cutoff ){
                        $cutoff_hit = 1;
                        my $current_segment_length = $i - $current_start;
                        if( $current_segment_length > $best_length ){
                                $best_length           = $current_segment_length;
                                $best_start_index = $current_start;
                        }
                        $current_start = $i + 1;
                }elsif( $i == $original_length - 1){
                        my $current_segment_length = ($i + 1) - $current_start;
                        if( $current_segment_length > $best_length ){
                                $best_length = $current_segment_length;
                                $best_start_index = $current_start;
                        }
                }
        }
        if( !$cutoff_hit ){
```

```
                $best_length = $original_length;
            }
        }
        if( !defined($segment_hist[ $best_length ] ) ){
            $segment_hist[ $best_length ] = 0;
        }
        $segment_hist[ $best_length ]++;
        $segment_sum += $best_length;
        $segment_count++;
        if (exists $hash{$best_length}) {
                $hash{$best_length}+=1;
                    }
        else{
                $hash{$best_length}=1
                    }
        if( $bwa ){
            if( $best_length <= 1 && $bad_first ) {
                $seq_string = "N";
                $quality_string = $poor_quality_char;
            }else{
                $seq_string = substr($seq_string, 0, $best_length);
                $quality_string = substr($quality_string, 0, $best_length);
            }
        }else{
            if ($best_length <= 0) {
                $seq_string = "N";
                $quality_string = $poor_quality_char;
            } else {
                $seq_string = substr($seq_string, $best_start_index, $best_length);
                $quality_string = substr($quality_string, $best_start_index, $best_length);
            }
        }
        print OUTPUT $ID1, $seq_string, "\n", $ID2, $quality_string, "\n";

    }
    my $segment_mean = sprintf( "%.1f", $segment_sum / $segment_count );
    my $halfway_index = $segment_count / 2;
    my $current_sum      = 0;
    my $current_index = 0;
    my $median_index1;
    my $median_index2;
    while( !defined( $median_index1 ) || !defined( $median_index2 ) ){
        if( defined( $segment_hist[ $current_index ] ) ){
```

```
			$current_sum += $segment_hist[ $current_index ];
		}
		if( $current_sum > $halfway_index ){
			if( !defined( $median_index1 ) ){
				$median_index1 = $current_index;
			}
			if( !defined( $median_index2 ) ){
				$median_index2 = $current_index;
			}
		}elsif( $current_sum == $halfway_index 	&& !defined( $median_index1 ) ){
			$median_index1 = $current_index;
		}
		$current_index++;
	}
	$current_index--;
	my $segment_median;
	if( $segment_count % 2 == 1){
		$segment_median = $median_index1;
	}else{
		$segment_median = sprintf( "%.0f", ( ( $median_index1 + $median_index2 ) / 2 ) );
	}
	print STDOUT "Info: $output_file: mean segment length = $segment_mean, median
segment length = $segment_median\n";
	close INPUT or die "Error: Cannot close $input_file: $!";
	close OUTPUT or die "Error: Cannot close $output_file; $!";
	my $segments_filename;
	if ( $directory ){
		$segments_filename="$directory/$filename.trimmed_segments";
					}
	else{
		$segments_filename="$filename.trimmed_segments";
				}
	open(SEGMENTS, ">$segments_filename");
	print SEGMENTS "Read_length\tProportion_of_reads\n";
	my $i;
	for ($i=0;$i <= $original_length; $i++){
		if (exists $hash{$i}){
			my $percentage=$hash{$i}/$segment_count;
			print SEGMENTS "$i\t$percentage\n";	}
		else{print SEGMENTS "$i\t0\n";
						}
						}
	close SEGMENTS or die "Error: Cannot close $segments_filename; $!";
}
```

# Script S4

:::::::::::::
fastqTrim_Length.pl: This program was used to delete the fastq reads which have length less than a specific value as well as to erase the “orphanage” reads (single reads without pair).
This program is made by Tiange Lang.
:::::::::::::

```
#!/usr/bin/env perl

use strict; use warnings; use Getopt::Long; use File::Spec;

my $length = 25;
my $paired = 0;
my $directory;
my $usage = "
$0 one single-end or two paired-end FASTQ files [-l|length 25] [-d|directory path]\n
-l|length            length cutoff [defaults to 25 nucleotides]
-d|directory         path to directory where output files are saved
\n";
GetOptions(
	"l|length=i"        => \$length,
	"d|directory=s"     => \$directory
);
my @files = @ARGV;
if( !$files[0] || length(@files) > 2 ){
	die $usage;
}
if( scalar(@files) == 2 ){
	$paired = 1;
}
unless( -e $files[0] ){
	die "error: file $files[0] does not exist\n";
}
open( FIRST, "<$files[0]" )
	or die "error: failure opening $files[0] for reading: $!\n";
if( $paired ){
	unless( -e $files[1] ){
		die "error: file $files[1] does not exist\n";
	}
	open( SECOND, "<$files[1]" )
		or die "error: failure opening $files[1] for reading: $!\n";
}
my $first_line;
my $second_line;
```

```
$first_line = <FIRST>;
if( substr($first_line, 0, 1) ne "@" ){
	die "error: $files[0] does not appear to be in FASTQ format\n";
}
if( $paired ){
	$second_line = <SECOND>;
	if( substr($second_line, 0, 1) ne "@" ){
		die "error: $files[1] does not appear to be in FASTQ format\n";
	}
}
my $first_id;
my $second_id;
if( $paired ){
	if( $first_line !~ /\S+\s\S+/ ){
		if( $first_line =~ /(\S*)\/\S*/ ){
			$first_id = $1;
		}else{
			$first_id = $first_line;
		}
	}elsif( $first_line =~ /\S+\s\S+/ ){
		my @first_line_elements = split( /\s+/, $first_line );
		pop @first_line_elements;
		$first_id = join( " ", @first_line_elements );
	}else{
		$first_id = $first_line;
	}
	if( $second_line !~ /\S+\s\S+/ ){
		if( $second_line =~ /(\S*)\/\S*/ ){
			$second_id = $1;
		}else{
			$second_id = $second_line;
		}
	}elsif( $second_line =~ /\S+\s\S+/ ){
		my @second_line_elements = split( /\s+/, $second_line );
		pop @second_line_elements;
		$second_id = join( " ", @second_line_elements );
	}else{
		$second_id = $second_line;
	}
	if( $first_id ne $second_id ){
		die "error: files $files[0] and $files[1] do not seem to be paired\n";
	}
}
if( $paired ){
```

```
		my $first_line_counter = 0;
		my $second_line_counter = 0;
		$first_line_counter++ while <FIRST>;
		$second_line_counter++ while <SECOND>;
		if( $first_line_counter != $second_line_counter ){
			die "error: files $files[0] and $files[1] appear to be different lengths\n";
		}
	}
	seek(FIRST, 0, 0);
	if( $paired ){
		seek(SECOND, 0, 0);
	}
	my $single_file;
	if ( $directory ){
		$directory =~ s/\/\z//;
		my @file_ending_elements = split(/\//, $files[0]);
		my $item = scalar @file_ending_elements - 1;
		my $file_name = $file_ending_elements[$item] . ".single";
		$single_file = File::Spec->catpath( undef, $directory, $file_name );
	}else{
		$single_file = $files[0] . ".single";
	}
	if( -e $single_file ){
		die "error: file $single_file already exists\n";
	}
	open( SINGLE, ">$single_file" )
		or die "error: failure opening $single_file for writing: $!\n";
	my $discard_file;
	if ( $directory ){
		$directory =~ s/\/\z//;
		my @file_ending_elements = split(/\//, $files[0]);
		my $item = scalar @file_ending_elements - 1;
		my $file_name = $file_ending_elements[$item] . ".discard";
		$discard_file = File::Spec->catpath( undef, $directory, $file_name );
	}else{
		$discard_file = $files[0] . ".discard";
	}
	if( -e $discard_file ){
		die "error: file $discard_file already exists\n";
	}
	open( DISCARD, ">$discard_file" )
		or die "error: failure opening $discard_file for writing: $!\n";
	my $paired_file1;
	my $paired_file2;
```

```
if( $paired ){
	if ( $directory ){
		$directory =~ s/\/\z//;
		my @file_ending_elements = split(/\//, $files[0]);
		my $item = scalar @file_ending_elements - 1;
		my $file_name = $file_ending_elements[$item] . ".paired1";
		$paired_file1 = File::Spec->catpath( undef, $directory, $file_name );
	}else{
		$paired_file1 = $files[0] . ".paired1";
	}
	if( -e $paired_file1 ){
		die "error: file $paired_file1 already exists\n";
	}
	open( PAIRED1, ">$paired_file1" )
		or die "error: failure opening $paired_file1 for writing: $!\n";
	if ( $directory ){
		$directory =~ s/\/\z//;
		my @file_ending_elements = split(/\//, $files[0]);
		my $item = scalar @file_ending_elements - 1;
		my $file_name = $file_ending_elements[$item] . ".paired2";
		$paired_file2 = File::Spec->catpath( undef, $directory, $file_name );
	}else{
		$paired_file2 = $files[0] . ".paired2";
	}
	if( -e $paired_file2 ){
		die "error: file $paired_file2 already exists\n";
	}
	open( PAIRED2, ">$paired_file2" )
		or die "error: failure opening $paired_file2 for writing: $!\n";
}
my $count_p1=0;
my $count_p2=0;
my $count_d=0;
my $count_s=0;
until( eof(FIRST) ){
	chomp( my $first_header_line1 = <FIRST> );
	chomp( my $first_sequence_line = <FIRST> );
	chomp( my $first_header_line2 = <FIRST> );
	chomp( my $first_quality_line = <FIRST> );
	my $second_header_line1;
	my $second_sequence_line;
	my $second_header_line2;
	my $second_quality_line;
	if( $paired ){
```

```
		chomp( $second_header_line1 = <SECOND> );
		chomp( $second_sequence_line = <SECOND> );
		chomp( $second_header_line2 = <SECOND> );
		chomp( $second_quality_line = <SECOND> );
	}
	if( $paired ){
		my $first_header_id;
		my $second_header_id;
		if( $first_header_line1 !~ /\S+\s\S+/ ){
			if( $first_header_line1 =~ /(\S*)\/\S*/ ){
				$first_header_id = $1;
			}else{
				$first_header_id = $first_header_line1;
			}
		}elsif( $first_header_line1 =~ /\S+\s\S+/ ){
			my @first_header_line_elements = split( /\s+/, $first_header_line1 );
			pop @first_header_line_elements;
			$first_header_id = join( " ", @first_header_line_elements );
		}else{
			$first_header_id = $first_header_line1;
		}

		# second of pair
		if( $second_header_line1 !~ /\S+\s\S+/ ){
			if( $second_header_line1 =~ /(\S*)\/\S*/ ){
				$second_header_id = $1;
			}else{
				$second_header_id = $second_header_line1;
			}
		}elsif( $second_header_line1 =~ /\S+\s\S+/ ){
			my @second_header_line_elements = split( /\s+/, $second_header_line1 );
			pop @second_header_line_elements;
			$second_header_id = join( " ", @second_header_line_elements );
		}else{
			$second_header_id = $second_header_line1;
		}
		if( $first_header_id ne $second_header_id ){
			die "error: header lines in $files[0] and $files[1] do not seem to be paired\n";
		}
	}
	if( $paired ){

		if( length($first_sequence_line) >= $length && length($second_sequence_line) >=
$length ){
```

```
			print PAIRED1 $first_header_line1, "\n", $first_sequence_line, "\n",
$first_header_line2, "\n", $first_quality_line, "\n";
			$count_p1+=1;
			print PAIRED2 $second_header_line1, "\n", $second_sequence_line, "\n",
$second_header_line2, "\n", $second_quality_line, "\n";
			$count_p2+=1;
		}
		elsif( length($first_sequence_line) < $length && length($second_sequence_line) <
$length ){
			print DISCARD $first_header_line1, "\n", $first_sequence_line, "\n",
$first_header_line2, "\n", $first_quality_line, "\n";
			$count_d+=1;
			print DISCARD $second_header_line1, "\n", $second_sequence_line, "\n",
$second_header_line2, "\n", $second_quality_line, "\n";
			$count_d+=1;
		}
		elsif( length($first_sequence_line) < $length && length($second_sequence_line) >=
$length ){
			print DISCARD $first_header_line1, "\n", $first_sequence_line, "\n",
$first_header_line2, "\n", $first_quality_line, "\n";
			$count_d+=1;
			print SINGLE $second_header_line1, "\n", $second_sequence_line, "\n",
$second_header_line2, "\n", $second_quality_line, "\n";
			$count_s+=1;
		}
		elsif( length($first_sequence_line) >= $length && length($second_sequence_line) <
$length ){
			print SINGLE $first_header_line1, "\n", $first_sequence_line, "\n",
$first_header_line2, "\n", $first_quality_line, "\n";
			$count_s+=1;
			print DISCARD $second_header_line1, "\n", $second_sequence_line, "\n",
$second_header_line2, "\n", $second_quality_line, "\n";
			$count_d+=1;
		}
	}
	else{
		if( length($first_sequence_line) >= $length ){
			print SINGLE $first_header_line1, "\n", $first_sequence_line, "\n",
$first_header_line2, "\n", $first_quality_line, "\n";
			$count_s+=1;
		}else{
			print DISCARD $first_header_line1, "\n", $first_sequence_line, "\n",
$first_header_line2, "\n", $first_quality_line, "\n";
			$count_d+=1;
```

```
		}
	}
}
my @name = split(/\./, $single_file);
my $summaryname= join '.', @name[0..$#name-1];
my $outputname = $summaryname.".summary.txt";
open(FILEOUT, ">$outputname");
if( $paired ){
print FILEOUT "paired1\t$count_p1\n",    "paired2\t$count_p2\n", "single\t$count_s\n", 
"discard\t$count_d\n";}
else{
print FILEOUT "single\t$count_s\n", "discard\t$count_d\n";}
close FILEOUT;
close FIRST or die "error: failure closing $files[0]: $!\n";
if( $paired ){
	close SECOND or die "error: failure closing $files[1]: $!\n";
}
close SINGLE or die "error: failure closing $single_file: $!\n";
close DISCARD or die "error: failure closing $discard_file: $!\n";
if( $paired ){
	close PAIRED1 or die "error: failure closing $paired_file1: $!\n";
	close PAIRED2 or die "error: failure closing $paired_file2: $!\n";
}
exit 0 or die "error: $0 ended abnormally: $!\n";
```

# Script S5

:::::::::::::

make_consensus_clustalw_dna.pl: This program was used to create consensus from the alignment file produced by CLUTALW.

This program is made by Tiange Lang.

:::::::::::::

```
#!/usr/bin/env perl

$input=shift;
open IN, "<$input";
open OUT, ">consensus.fa";

while ($line=<IN>){
        chomp $line;
        $len = length($line);
        last;
}

$site="";
for ($i=0;$i<$len;$i++){
        $a=0;$t=0,$c=0;$g=0;$ins=0;@nb=();$hit=0;
        open IN, "<$input";
        while ($seq=<IN>){
                chomp $seq;
                $hit++;
                print "Pos:",$i+1,"\tSeqNo:",$hit,"\n";
                $base=substr($seq,$i,1);
                if (uc$base eq "A"){$a++;}
                if (uc$base eq "T"){$t++;}
                if (uc$base eq "C"){$c++;}
                if (uc$base eq "G"){$g++;}
                if ($base eq "-"){$ins++;}
                print "A:",$a,"\tT:",$t,"\tC:",$c,"\tG:",$g,"\tIns:",$ins,"\n";
        }
        @nb=($a,$t,$c,$g,$ins);
        @nb=sort{$b<=>$a}@nb;
        if ($nb[0]==0){$site="N";}
        else {
                if ($nb[0]==$a){$site="A";}
                elsif ($nb[0]==$t){$site="T";}
                elsif ($nb[0]==$c){$site="C";}
                elsif ($nb[0]==$g){$site="G";}
                else {$site="-";}
```

```
        }
        print "Nt:",$site,"\n\n";
        print OUT $site;
}
print OUT "\n";
```

## Sequence S6

Refseq genome DNA sequence of human MUC2: NC_000011.10 Homo sapiens chromosome 11, GRCh38.p14 Primary Assembly (1074902-1110352)

ATGGGGCTGCCACTAGCCCGCCTGGCGGCTGTGTGCCTGGCCCTGTCTTTGGCAGGGGGCTCGGA
GCTCCAGACAGgtgagagagcagacacaggggtctggggcctggcagagtgtcctgggggcaggg
cgaggcgggcgggcaagtcgcgtctgggaggaggagctggtcccagagtgcagcctgcgcggctc
tgctgaggctcctggcccgggttggtccctggaagcccccggccctgctgactttcaaggagctg
gaaggtcggggctcccctgctattcctttggggttgactgcccgacgacagtgtgggtcttgggg
ccagcaccaggtggaaacagcaggtcaggccccagtgaactgggtcattgtccataggggaggaa
ggggtggccaggatcccaccagaaggccccattctcaggtggcagagacccttgaagagttgggg
cagcacagcccttgctggggagcggggtgcccagaatgccctctcctacatcccgcttggcaccc
ggccgcactcctcaccaggccgggggtagaagccctgagacccctgtggtggggtgaccaaggcc
cagcagagggcccgaggataggaaggaacctttcccggccaggggccctgtgctgggctcgaagc
tgcttccaggtgcttcttcaggggccttctctcgagggtagcttgggcagccttcccccctccggg
gccactcacccctcattccccgctgctccctcagAGGGCAGAACCCGAAACCACGGCCACAACGT
CTGCAGCACCTGGGGCAACTTCCACTACAAGACCTTCGACGGGGACGTCTTCCGCTTCCCCGGCC
CCTGCGACTACAACTTCGCCTCCGACTGCCGAGGCTCCTACAAGGAATTTGCTGTGCACCTGAAG
CGGGGTCCGGGCCAGGCTGAGGCCCCCGCCGGGGTGGAGTCCATCCTGCTGACCATCAAGGATGA
CACCATCTACCTCACCCGCCACCTGGCTGTGCTTAACGGGGCCGTgtgagtgtggtcggtggcac
ccctcccacatcctagcaacgggggctgatgtttcccaaagggatattccttgtagccctagaag
accccttccgccccagcacacagctcaggagaacagccttgaggtttgggttcaggtcactaatt
cattcaacaaacactgatgagcccccaccattccccccataggcaaggggtttcagttatccctt
tgcctgtgtgtccctgacagcccctcccctcggagcccaccaggctccggacagacttggcaccc
ctggaggctgcatgtctctggtcctgtgcatggagtggccgtgtgtgccctccccaggctagagt
tacagaagccggtgcagggggctgtgggaccccccttccccatccccagctattgctcccctattg
tctccagaacaatgaggccctgtaagtgcgttcccatccagcgcctgcccctcttctgcctgggg
atttagtttcctgcaaggcgccccagcatgggcatgggcaggcgggtggaggccctcaggcatgg
gcatgggcaggcgggtgggtagaggccctcaggcgtgagtgcgggcgggtgggtggatagaagcc
gtcaggcatgggtgcaggcgggtgggtagaggtcctcaggtgtgggcatgggcaggtgggtgggt
agaggccgtcaggtgtgggcgcgggtgggtgggtagaggccctcaggcatgggtgcaggcgggtg
ggtgggtagaggccctcaggcgtgggcgcgggtgggtggatagaggccgtcaggcgtaggtgcgg
gcgggtgggtagaggtcctcaggtgtgggcgcaggtgggtgggtgggtagaggccctcaggcatg
gcacaggtgggtgggtagaggccctcaggcatgggcgcaggcgggtgggtgggtaggggccctca
ggcatgggtgttggcaggtgggtgggtagaggctttcaggcatgggcaggcaggtagaggccctt
gaggaccgaggcacagaggctggggtgagtgcctctacctggaccagcaaggggcactggcagga
ggtggggtagggcccctgacgttctcaggggcagcctggggggctctgggggggtttgggacccca
tggggggatgttccaccaagcagggggcctggaagggggctgggcagcctggtcctccctcctct
cccaacctggtgccctcagggcctctgagggggggaccctgcccaggaccgtgccccgaggaggga
gtggagaggaggggcgtgcaggcaggaggtggctctgccggggaagcccggccagcggagatgga
caggtgctctttggccactgcctatgtccctccaccccagaggccggccaagttggtgatcccag
ggcaggagctgggcctggcagagccatctccaccaccccaggtgcccagcttcagtcccctctgg
gcggcggggtcccgggaggacaagctggggcgggggggcctgggtggtggacccaagagtgaccc

cgatgtgcctccgccagGGTCAGCACCCCGCACTACAGCCCCGGGCTGCTCATTGAGAAGAGCGA
TGCCTACACCAAAGTCTACTCCCGCGCCGGCCTCACCCTCATGTGGAACCGGGAGGATGCACTCA
TGgtgctcaggggtccccggactcgtggggctggtgggggctccgtcaggcctctgggcagaccc
caagggagggcagggagggcagtgctctgacccctcaccgagagggcatgggtggggcagggcct
cggcagcgcagggcgtcggtgctggacttgggggcagcagcagaagccgacctggccctgaccc
ccccaggcctcagccttcccccaaacgcactcggcttctcagggacctgccctgccaggccgctc
cctggctgctgaccccagccttcctgccccaccttcctctggctcaaacaagccacgagtcttgg
gggttcctggcggctgtgggccgggcgggaggccagctcacctgctccctcccgcaacagCTGGA
GCTGGACACTAAGTTCCGGAACCACACCTGTGGCCTCTGCGGGGACTACAACGGCCTGCAGAGCT
ATTCAGAATTCCTCTCTGACGgtgaggcccggagggcttggagggggcagggtaggctacgggcc
cccaggagccctagctgaagggccgtgcatccccagGCGTGCTCTTCAGTCCCCTGGAGTTTGGG
AACATGCAGAAGATCAACCAGCCCGATGTGGTGTGTGAGGATCCCGAGGAGGAGGTGGCCCCCGC
ATCCTGCTCCGAGCACgtgagtcccctcggtccggggtgggggtcctggcggagctggcctctga
atagcatgctcaccctgcgtctgtccccagCGCGCCGAGTGTGAGAGGCTGCTGACCGCCGAGGC
CTTCGCGGACTGTCAGGACCTGGTGCCGCTGGAGCCGTATCTGCGCGCCTGCCAGCAGGACCGCT
GCCGGTGCCCGGGCGGTGACACCTGCGTCTGCAGCACCGTGGCCGAGTTCTCCCGCCAGTGCTCC
CACGCCGGCGGCCGGCCCGGGAACTGGAGGACCGCCACGCTCTGCCgtaagccccggcgccttgt
gggcaggggaccccagggagaccccacgctggtgctttccccaagcccgggtgggagctgtgtct
gtgccgggcaccttgagctgggggacactcaccgcaccgggcaccttgagctgggggaacacta
accgtgccgggcaccgggagctgggggacactcaccgtgccgggcaccttgagctgggggaca
ctcaccgtgccgggcaccgggagctgggggacactcaccacgggcaccgagagctgggggaca
ctcaccgtgccgggcaccgggagctgggggacactcaccgtgacgggcaccgggagctgggggg
acactcaccgtgacgggcaccgggagctgggggacactcaccgtgccgggcaccgggagctggg
gggacactcaccacgggcaccgggagctgggggacactcaccgtgccgggcaccttgagctggg
gggacactcaccgtgccgggcaccgggagctgggggacactcaccgtgccgggcaccgggagct
gggggacactcaccgcgccgggcaccgggagctgggggacactcaccgtgccgggcaccggga
gctgggggacactcaccacgggcaccgagagctgggggacactcaccgcgccgggcaccggga
gctgggggacactcactgtgacgggcaccgggagctgggggacactcaccgtgccgggcaccg
ggagctgggggacactcaccacgggcactgggagctgggggacactcactgagggcaccggga
gctgggggacactcactgtgacgggcaccgagagctgggggacactcactgtgacgggcaccg
ggagctgggggacactcactgtgacgggcaccgggagctgggggacactcaccgtgccgggca
ccgggagctgggggacactcactgagggcaccgggagctgggggacactcaccgtgccgggca
ccgggagctgggggacactcaccacgggcaccgggagctgggggacactcaccgtgccgggca
ccgggagctgggggacactcaccgtgccgggcaccgggagctgggggacactcactgagggca
ccgggagctgggggacactcactgtgacgggcaccgagagctgggggacactcactgtgacgg
gcaccgggagctgggggacactcactgtgacgggcaccgggagctgggggacactcaccgtgc
cgggcaccgggagctgggggacactcactgagggcaccgggagctgggggacactcaccgcgc
cgggcaccgggagctgggggacactcactgagggcaccgagagctgggggacactcactgtga
cgggcaccgggagctgggggacactcaccgcgccgggcaccgggagctgggggacactcaccg
tgacgggcaccgagagctgggggacactcactgtgacgggcaccttgagctgggggacactca
ccacgggcactgggagctgggggacactcaccgcgccgggcaccgggagctgggggacactca
ctgagggcaccgggagctgggggacactcaccgtgccgggcaccgggagctgggggacactca
ctgagggcaccgggagctgggggacactcactgagggcaccgggagctgggggacactcactg
agggcaccaagagctgggggacactcaccacgggcaccgagagctgggggacactcaccgtga

cgggcaccgggagctgggggacactcaccacgggcaccgggagctgggggacactcaccgtga
cgggcaccgggagctgggggacactcactgagggcaccgggagctgggggacactcaccacgg
gcaccgggagctgggggacactcaccgcgccgggcaccgggagctgggggacactcaccacgg
gcactgggagctgggggacactcaccacgggcactgggagctgggggacactcaccacgggca
ccgggagctgggggacactcaccgtgacgggcaccgggagctgggggacactcaccacgggca
ccgggagctgggggacactcaccacgggcaccgggagctgggggacactcaccacgggcaccg
ggagctgggggacactcaccgcgggcactgggagctgggggacactcaccacgggcactggga
gctgggggacactcaccacgggcaccgggagctgggggacactcaccgtgacgggcaccggga
gctgggggacactcaccacgggcaccgggagctgggggacactcaccacgggcaccgggagct
gggggacactcaccacgggcaccgggagctgggggacactcaccgtgccgggcaccgggagct
gggggacactcactgagggcaccgggagctgggggacactcaccacgggcaccgagagctggg
gggacactcactgtgccgggcaccgggagctgggggacactcaccacgggcaccgggagctggg
gggacactcaccgtgacgggcaccgggagctgggggacactcaccacgggcaccgggagctggg
gggacactcaccgtgccgggcaccgggagctgggggacactcactgagggcaccgggagctggg
gggacactcaccacgggcaccgagagctgggggacactcactgtgccgggcaccgggagctggg
gggacactcaccacgggcaccgggagctgggggacactcaccgtgacgggcaccgggagctggg
gggacactcactgagggcaccgggagctgggggacactcaccacgggcaccgggagctgggggg
acactcaccacgggcaccgggagctgggggacactcaccgtgccgggcaccgggagctgggggg
acactcaccacgggcaccgggagctgggggacactcaccacgggcaccgggagctgggggaca
ctcaccgcgccgggcaccgggagctgggggacactcaccgtgggctgagagcccttctcggtgc
acttcggggtggagcggctgctgtgccccagcctcaccctcactgcgtggcctctgcggttccag
CCAAGACCTGCCCCGGGAACCTGGTGTACCTGGAGAGCGGCTCGCCCTGCATGGACACCTGCTCA
CACCTGGAGGTGAGCAGCCTGTGCGAGGAGCACCGCATGGACGGCTGTTTCTGCCCAGAAGgtgc
gtgtggaggatggccccgccctggcactgcccaccagatgagaggcagccctggcctggggttct
cgcctgcgctgaggggacggctccgctgggtggtgggggcagcggcggcacagaagtgcctctcc
ctccacccgataccgggggagaaggggcctcggtgtgaggcccttcccaaagggtggcttcaggg
aggccgggaagggggctgccttcctggttatcaccctggggacagacctcctcctgcccggcccc
tggcctggtgcctgaggcctttgggagcagctcgattgtcaggggcaggaaggtggcctggaggc
tggacccccatggccagaccccaacccagggaccaggtggggaccgcaggcgtcagcacagggga
ccagtggtgcctgcgggtgggaggcctggctggcagcccctcggtggggattctggctctttctg
agccagccggggtgacatcgcctccctggctgtcccagGCACCGTATATGACGACATCGGGGACA
GTGGCTGCGTTCCTGTGAGCCAGTGCCACTGCAGGCTGCACGGACACCTGTACACACCGGGCCAG
GAGATCACCAATGACTGCGAGCAGTGgtgagtcccggggccagggctgggcacagcagaggctgg
ggcggctgagccctgaccctgtgccccgctgcccaacagTGTCTGTAACGCTGGCCGCTGGGTGT
GCAAAGACCTGCCCTGCCCCGGCACCTGTGCCCTGGAAGGCGGCTCCCACATCACCACCTTCGAT
GGGAAGACGTACACCTTCCACGGGGACTGCTACTATGTCCTGGCCAAGgtaggctgcccagggtc
tggggcatggggcagagctggggctggcatccaggcccttggctgtcccggggtgggtgggctgg
ctgtccctgaagcagagggtgcctgtgggctgtcctggggcaggtgaccatgcttctgctctctg
gctggagaataagaagcaggccttcctttctaagccactgccgggtcctagggtgcagggtgctg
cccgtcccggccctcagcagctgcactgcctcttgccccatcacagGGTGACCACAACGATTCCT
ACGCTCTCCTGGGCGAGCTGGCCCCCTGTGGCTCCACAGACAAGCAGACCTGCCTGAAGACGGTG
GTGCTGCTGGCTGACAAGAAGAAGAATgtgagtggtcctgccccctccttctggagccccaggtc
ccccgagggggcccttctcagccctgagcaacctcggccttccctgcagGTGGTGGTCTTCAAG
TCCGATGGCAGTGTACTGCTCAACGAGCTGCAGGTGAACCTGCCCCACGTGACCGgtgagttgcg

ccccagggaggggcccgggcccttcgagctccactgggcctgcagtgattcggacagtccagcca
cctcggacccaggaggctgggtgggaaggttccacgggggagggtccctgcggcacccagcagg
ctccgtcctgggtcctctgctggaggggtggtgggagggtgacaccctcccgctgctcacctgg
gccaggcaggtcccgggagccccgcccctcgccatgccccttaccgtgtccctcatcgtgcccct
gcccacagCGAGCTTCTCTGTCTTCCGCCCGTCTTCCTACCACATCATGGTGAGCATGGCCATTG
GCGTCCGGCTGCAGGTGCAGCTGGCCCCAGTCATGCAACTCTTTGTGACACTGGACCAGGCCTCC
CAGGGGCAGGTGCAGGgtaagtggccccaccggggttgccccaacaaaggcccacaggggggcct
gctagccccagactcttcccaaccctgtcctggcccctcagGCCTCTGCGGGAACTTCAACGGCC
TGGAAGGTGACGACTTCAAGACGGCCAGCGGGCTGGTGGAGGCCACGGGGGCCGGCTTTGCCAAC
ACCTGGAAGGCACAGTCAACCTGCCATGACAAGCTGGACTGGTTGGACGATCCCTGCTCCCTGAA
CATCGAGAGCGgtgaggctcggcaacacgggcgcccccacctagcgtgcctagggtacccggccc
atggcctggaagggcagacggggctcccagcaggaagcatgggtggtgaggggcagaagtgaggt
ggctctcctccaggggcagcccggcccctgctgcttcctgctgtggctagtttatggcggccatg
gtggcagcctgccaggtgacctggaagagggcctgggctggtccctacctgccccgtcatgtcca
ggatgctgggcccttgggggtgagagacgggaggtggtgggtgccctgcaggggtttctatctag
ccaggagctgcctggaaatttgactcacggggaggaaggggcctgggcatcggtgcacagaggga
accatatctggggcctaggcagccaggcagcagggcccaggggatctcacgggggtcccgggccc
cgctgaagttccgatcccccactccccagCCAACTACGCCGAGCACTGGTGCTCCCTCCTGAAGA
AGACAGAGACCCCCTTTGGCAGGTGCCACTCGGCTGTGGACCCTGCTGAGTATTACAAGgtgggt
gggacccacacccccaggcccccatgccatcaaggtggactcagggcacccccagcccccatgc
cacccgtgaggtggactcagagcacccggttgggcccactggttgctgtgtgtgcgtgtgagctt
gcgtctgtgagcgccaggccacactctgcctccctgcctcactgcccgtccaccttgctctgtcg
cccagAGGTGCAAATATGACACGTGTAACTGTCAGAACAATGAGGACTGCCTGTGCGCCGCCCTG
TCCTCCTACGCGCGCGCCTGCACCGCCAAGGGCGTCATGCTGTGGGGCTGGCGGGAGCATGTCTG
CAgtgagtgccgtccccgtgggctgcatcctggggatggggtccgggctttgagctcctgggacg
gggctgggggccctgagcacgggtggtccagggagaggggttggcccccctgcagccacggaccag
gctccagcttcgtcggccggtggtagcaggaaaccagcaactcctatagcaaggggcggccacgt
agcaggggcagaacctggggtgggcctggagctgtggcggccgagtgtgggagtgggtcccagag
tgtgcactccctggccccctggccaccctggggatgggagctgggcgtctggctcttcccgtccc
tcacaccaccccgtggtcctctgcagACAAGGATGTGGGCTCCTGCCCCAACTCGCAGGTCTTCC
TGTACAACCTGACCACCTGCCAGCAGACCTGCCGCTCCCTCTCCGAGGCCGACAGCCACTGTCTC
GAGGGCTTTGCGCCTGTGGACGGCTGCGGCTGCCCTGACCACACCTTCCTGGACGAGAAGGGCCG
CTGCGTACCCCTGGCCAAGTGCTCCTGTTACCACCGCGGTCTCTACCTGGAGGCGGGGGACGTGG
TCGTCAGGCAGGAAGAACGATGgtgggtacctgctcgggggtcaggtgtggcgtgggggcggggg
aactccttctgaacctgccccaagcggagacctgggagtctctacctggggaagctgagacaccc
aaggctgaggggtgcctggggtgggggcgctgagaggcatcaggctcacatctgcggggaagct
gctggctgtctgtggccgtcctgcatgggccccgctcatccctggcctttccacagTGTGTGCC
GGGATGGGCGGCTGCACTGTAGGCAGATCCGGCTGATCGGCCAGAgtaagtggcactgccccggc
cacccctccccagccacccctccctgcctgccctggccaccctccccggccacccctcccgggcc
tgcctgagaccctcagcttcagctggagctgaggtggcccctccgtcccacagGCTGCACGGCCC
CAAAGATCCACATGGACTGCAGCAACCTGACTGCACTGGCCACCTCGAAGCCCCGAGCCCTCAGC
TGCCAGACGCTGGCCGCCGGCTATgtgcgtgttgggggcgctgctgtgggcgggcagggattcct
ggctggctgagcctggctcttgtgctgtgcccccgctagggtctgggtgccgagtcctgaggacg
caggccctgttgatgctgtccctggccctgggagggaagtggcagcctgtgagccactggggcac

aggggccagtgtagggcccttggccggcagccctcaccagtctcactgccctgtggcgggcccaa
ggggagggaagcctgagcccaggccagggggagtggtgggaggtctgggacatgacagagactgc
atggtcaggcctttcctggttgcacatccaatcctgaccccagggagggctgcagcctcacctgt
ccacccctgaaccccactctctggctgtccccagTACCACACAGAGTGTGTCAGTGGCTGTGTGT
GCCCCGACGGCTGATGGATGACGGCCGGGGTGGCTGCGTGGTGGAGAAGGAATGCCCTTGCGTC
CATAACAACGACCTGTATTCTTCCGGCGCCAAGATCAAGGTGGACTGCAATACCTGgtaagctgg
cccggcctgtcctggctgcctcccaggccccacgtgctccgcaggggtggccactggagagcggt
ccaaggggcaagtgcctctcctggggggttccgcctgggtcttgcgagatcctgtggtggcccctg
tcccacgggcagggtggtctctcatgtcaaccgctggtcttgaagccatggggggaagggacattt
ggagccacttttggggcctgcaggtgtcctgtgtgggaggcacagggagctgtctgcacggtgcc
cagggtctcctccagccacccatgagcaggtcctgggtcccttcaggctcctctcctgtcctcct
cagCACCTGCAAGAGAGGACGCTGGGTGTGCACCCAGGCTGTGTGCCATGGCACCTGCTCCATTT
ACGGGAGTGGCCACTACATCACCTTTGACGGGAAGTACTACGACTTTGACGGACACTGCTCCTAC
GTGGCTGTTCAGgtgtggtcacgggcactgcctggtcgggctgcttatggtcagggaccctctgc
ctgccccaagtgcagtgcttagctccccgagaaaccctgagacttgggaaggccggcctttcctc
agccccagacccgcacctgcacccgcaggaggattcgttcttctagccagggctgggtaggggtg
gtaaaacccctctgtactgcccagttctgtggttctcctctgggtcctcctccgggtcctcctcc
gggtcctcatctgggtcctccctcctctggcctcctctgggtcctccctcctctgggtcctcctc
tgggtcctccctcctctggcctcctctgggtcctccctcctctgggtcctccctcctctgggtcc
tcctccaggtcctcctctgggtcctccctcctctgggtcctccctcctctgggtcctcctccagg
tcctcctctgggtcctccctcctctgggtcctcctctgggtcctcctctgagtcctccctcctct
gggtcctcctctaggtcctcctctgtggtcctcatttgggtcctcctctgggtcctcctctgggt
ccttctctgggtgcacaaggtgggtgcaccagccatggggactgagggcacctgtttggggagct
gagtaaaggccagggctaggccgctgcccgcgcggctctccagatccaaatcccacagccctttg
aggcaccgtgatccccagggacaggggacaggcctgcagcagggtcaggtccttggatgggccag
gccagggcctggtttgtctgctcagtggctgtgaccctgccaactggggcgggtgtgccccggga
cacctggggtccagctgtcctggctgaccttgccctcctggcccccagGACTACTGCGGCCAGAA
CTCCTCACTGGGCTCATTCAGCATCATCACCGAGAACGTCCCCTGTGGCACTACGGGCGTCACCT
GCTCCAAGGCCATCAAGATCTTCATGGGGgtgagtgctgctggccctggggacgcgtgagccctg
cgggaccctcagaccagccagtgactgggcctctcctccgggcagAGGACGGAGCTGAAGTTGGA
AGACAAGCACCGTGTGGTGATCCAGCGTGATGAGGGTCACCACGTGGCCTACACCACGCGGGAGG
TGGGCCAGTACCTGGTGGTGGAGTCCAGCACGGGCATCATCGTCATCTGGGACAAGAGGACCACC
GTGTTCATCAAGCTGGCTCCCTCCTACAAGgtgggctgcctccctgcctgccctgccccctcctg
gccagccccccacccccctgccctggtgtttgcaggacaagcccctgtcctccctccagcccccttt
ttggagcccctgtgatgcttgtctcttgcagGGCACCGTGTGTGGCCTGTGTGGGAACTTTGACC
ACCGCTCCAACAACGACTTCACCACGCGGGACCACATGGTGGTGAGCAGCGAGCTGGACTTCGGG
AACAGCTGGAAGGAGGCCCCCACCTGCCCAGATGTGAGCACCAACCCCGAGCCCTGCAGCCTGAA
CCCGCACCGCCGCTCCTGGGCCGAGAAGCAGTGCAGCATCCTCAAAAGCAGCGTGTTCAGCATCT
GCCACAGCAAGgtgggctggccgggccatggtggggcaagtaggcagaggagggctgtaggtggg
ctgtgactgtgggctggggccatgggcggggccgactgtaggcagagcagggctgtagggggcct
gtgactataggccggggcatggcggggctaactaggcagagcagggctgtaggtgggctacagct
gtgggcggggccatgggcggggccgactaagcagagcagggctgtaggtgggctatagctgtggg
cggggccacgggcggggccgactgtaggcagagcagggctgtaggtggactatagctgtgggcgg
ggccatgggcggggccgactgtaggcagagcagggctgtaggtgggctgtggctgtgggcggggc

cgactaggcagagcggggctatgggctgactgtggacgtggtgagggtgccgtagagcatgctaa
tgaccagggcgtggtcatagcagggtagggtcttgggtgctcctggggctgggggcttctccac
atgctccccacaccttcaggagtcgccctgctgcgtcacgcaccacacggcgcttgtcctccagc
tttggctctggccgctgcctcctttggtcacatgaccgtataatcggcctcccctctgagaccct
gggctggacccccggcctccctctgcctccccaggctcagatattcacccggagggagaaaggac
atgtgtcccccatgcccacacatccccagctacaggcagctggggaggacgggttctaggatggc
catgttacagctgaggatgcagaggggttgggtgatgggtctgcacagccacggcgggacaggtg
tctctggaccctctccccaaggttggccctgccggggccctggctggctggtgctgggtaatgtg
ccctgtcccaggagcagggccggcctcagggtcctgagctccagggcactggggaagtcctggct
ccatgagggcagcacgggcccaggacagaccagggtgttctccccagGTGGACCCCAAGCCCTTC
TACGAGGCCTGTGTGCACGACTCGTGCTCCTGTGACACGGGTGGGGACTGTGAGTGCTTCTGCTC
TGCCGTGGCCTCCTACGCCCAGGAGTGTACCAAAGAGGGGGCCTGCGTGTTCTGGAGGACGCCGG
ACCTGTGCCgtaagagcctgcccgaactgcactcagggccgggacgggggctgggaggtgctgta
ttgcgggccggggtgacactccttgtccatccaggtgatgggtgtgcatcacccaccctttcccc
gacttctccagtgtccttctttggggccctgtgggacccgggttggcagagcaagcttgatgcgt
ctgcgtcccagcccccgaccccagattcgccctcaccccggcccaggcctgagccctcctgcgtc
tgaccctggccctgtctcccccaagCCATATTCTGCGACTACTACAACCCTCCGCATGAGTGTGA
GTGGCACTATGAGCCATGTGGGAACCGGAGCTTCGAGACCTGCAGGACCATCAATGGCATCCACT
CCAACATCTCCGTGTCCTACCTGGAGGgtgagcagggtggggcgggcttcagcgggggtgatggc
cgaggggcctggaggctgagtggggcagccctcgggagaggcaacagtccactggcctggagggt
gagccaggcggccctcgggggaggctacggccgacgggcctggcactgtggggctgaaggctgat
gtctggagacccatggggacacccggagggaggcctgaccctcagggtacccacagcccagggca
gccaggctccccttgctgcaggatcaggagggaagcaggctatcgtggaaactgggagtggcagg
ggtgggaggtgctgaggttcgtgcagagcagggcgggttggggagcatttcaggcacaggtcagg
ggaggcccctgccgggtgctggtgtctgagctgagaaccagtgacgtgaaggagggactggtggg
aagtttgggaggagtatcccgccatgggagaggaacatgggtcttgggactcagggctgctcggg
gggcccgatgagactgggcagggctcctcagcaggcagcgttcagggctcagtggggtggggaga
tccaggccctgcctttccaatccccggccttcccagaggggcatcctgcagagaagggcctgcca
gggtagggacggtgggtggggtgtggtggactgcggtggtcccaaccctatgccctgtgtccacc
agGCTGCTACCCCCGGTGCCCCAAGGACAGGCCCATCTATGAGGAGGATCTGAAGAAGTGTGTCA
CTGCAGACAAGTGTGGCTGCTATGTCGAGGACACCCACTACCCACCTGGAGCATCGGTTCCCACC
GAGGAGACCTGCAAGTCCTGgtacctaagcccacgtggcaggggggcctgggggagctgcacatat
gggcacatgagtacacacacacgtgtgagcacacagtgtacacagtacacagacacacaaccgtt
ccacatgggtgcacatgcacacaaacgcacacagcataccacgtgcatacacacggtcacatgca
tgcatggtgcacacatgcacacatgaatggatgccaacatgcaggcacacacagtcacacatgca
cacagcgcacacatggacacatgcctagacgcagatacccaggcatacactcacggttacacact
cacgcacatatgcatggatgcagacacgcaggcacacacggtcatatagtcatacaccacatgca
cacatgcacagacacccaggcacacacagttacacagtcacacatgcacacatgcatggacgcag
acacgcaggtgcacacacacatgcacagtgcacacatgtacacatgcctagacacagatacccag
gcacacacagtcacacatgcatggacacagagtcacatgtgtacacatacacacgtgtggacaga
cataggcacagtcacgtgcacacatgcactcacactcagtcacacatgaacatgtgctcacatgc
atggacactgacacgcaaggacacacagtcacacatgcacacatgcatagacacagacacccagg
cacacacagttacacagtcacacatgcatggatgcagacacgcagtcacacagtcacacatgcac
acactgcacacatgtacacatgcctagacacagatatgcaggcacacacacatagtcaaacatgc

acacatgcatggacacaaagtcacacgtgcacacatgcacacatgcatggacagacacaggcaca
cacagtcacgtgcacagatgcactcacagtcacacatgaacacatgctcacatgcacagacactg
acacgcaggcacacacagtcacacatgtacacgtgcctagacacagatacccagacacacacaat
tacacagtcgcacagtcacacatgcatggatgcagacacacaggtacacaaggtcacacagtcat
ataatgcacacatgcacacatgcatagatacagacacccaggtacacactcacggtgacacagtc
acacatgcacacatgcatggaggcagacacacaagcacacacagtcacacagtcacacatgcaca
caggagccaggctacagaggtaccagtccctcactgcggcgggggtcttctgttctcatcccat
cctctgggtctggctttttccttcctctcctcgcccctgctctgttcccacagttacaacccagt
ggggggctcttccggagctggctttggggcagtgcctgggggctttgggctcggtactagccaca
tggggaagctgggggtctgagcagcgtgggcgcgttgtcagtggagtgggacttgtagccatgtg
cttgctttgcagCGTGTGTACCAACTCCTCCCAAGTCGTCTGCAGGCCGGAGGAAGgtaagctgc
cctctgctgccagccctgcggtggccgggcccatcctggggaagcctgtggggccttggatcggt
ggggggtgctggtctcctcctgggctctgcccctttggtcccccccagctcagacccacctccg
atgtgtatcagccctggggggctgctgtgacccattttgtttcttctggggtgtcggtgtcctgt
ggggaatttccgtcaccctctcccgtgatccagcttctgcgttctgatgagattccctttattca
aagagaggggctctgggacgggtgcagtctcactggagcatttcttagctgcttgtgggggctcg
ggcacacctggccttcttcctatcttgctcctgatgaggtgattcttggcctcaccctcaccccc
agGAAAGATTCTTAACCAGACCCAGGATGGCGCCTTCTGCTACTGGGAGATCTGTGGCCCCAACG
GGACGGTGGAGAAGCACTTCAACATCTGTTCCATTACGACACGCCCGTCCACCCTGACCACCTTC
ACCACCATCACCCTCCCCACCACCCCCACCACCTTCACCACTACCACCACCACCACCACCCCGAC
CTCCAGCACAGgtaaggcccccctggttccctccatgcttcctcgggctctcaccttcccctgcat
ccagcatccagcacagagggctctttcgggggcaggccccggcctggtgcagccaggctgtgacc
cctgcacaccagctgcagagtgaggtgacagtggcattcctctgcactgaggtgtgagggggcct
gccctggctcccctggcctggtgcattgagatagtagcatcctgaccacatccccaagcccagac
cacagtggaggatcacctggggagatttctgaaaaccagcaggaaactatccctaagggttagag
aaatttcttatgttcccctgcgtttgttctggttgaaatcctagctaccactgaacaagccacc
aggggtatgatagccacagaaaaaagaaactttttttaaaaaaggcaagattttaaaagatcttg
aactatataatgatatcctcttttcttcctgctttattgcagTTTTATCAACAACTCCGAgtaag
tgacggtgatgatattcatgatgacaagcagggtgggaggagcgaagtcttataaaatcacctgc
aggatgcttccttcagggcccagatgtgaggctggcggggctggactcctctgcttatggaccaa
agatggatgtattttggccacttcattcatggtttgctgaggccaggggctaaagtgagacctga
ttggctgtcggtgacaatattgctggttaagagtggagacaaagccccttccgtcacacttcctt
actggaatgggaagctctcttgttattgattctttgaaaaaaaagtattgaaaatagctgaggaa
agggtccatcacacccaggtgtggccctgggtggccccgtctctttgggctcaggttttcagttg
caaaatgaggatggaagtggtgtccagccctgagctctctggccctgcactctggtttttggca
atgacagggaaaagagagattgcagctgggggatggtcatggaggtccctgggtcctctgaatcc
tggtggcttcctggaggtgcctctccccaggtgtgagagacaagaacttggttttgcttccctag
AGCTGTGCTGCCTCTGGTCTGACTGGATCAATGAGGACCACCCCAGCAGTGGCAGCGACGACGGT
GACCGAGAAACATTTGATGGGGTCTGCGGGGCCCCTGAGGACATCGAGTGCAGGTCGGTCAAGGA
TCCCCACCTCAGCTTGGAGCAGCTAGGCCAGAAGGTGCAGTGTGATGTCTCTGTTGGGTTCATTT
GCAAGAATGAAGACCAGTTTGGAAATGGACCATTTGGACTGTGTTACGACTACAAGATACGTGTC
AATTGTTGCTGGCCCATGGATAAGTGTATCACCACTCCCAGCCCTCCAACTACCACTCCCAGCCC
TCCACCAACCAGCACGACCACCCTTCCACCAACCACCACCCCCAGCCCTCCAACCACCACCACAA
CCACCCCTCCACCAACCACCACCCCCAGCCCTCCAATAACCACCACGACCACCCCTCCACCAACC

ACCACTCCCAGCCCTCCAATAAGCACCACAACCACCCCTCCACCAACCACCACTCCCAGCCCTCC
AACCACCACTCCCAGCCCTCCAACCACCACTCCCAGCCCTCCAACAACCACCACAACCACCCCTC
CACCAACCACCACTCCCAGCCCTCCAACGACTACGCCCATCACTCCACCAGCCAGCACTACCACC
CTTCCACCAACCACCACTCCCAGCCCTCCAACAACCACCACAACCACCCCTCCACCAACCACCAC
TCCCAGTCCTCCAACGACTACGCCCATCACTCCACCAACCAGCACTACTACCCTTCCACCAACCA
CCACTCCCAGCCCTCCACCAACCACCACAACCACCCCTCCACCAACCACCACTCCCAGCCCTCCA
ACAACCACCACTCCCAGTCCTCCAACAATCACCACAACCACCCCTCCACCAACCACCACTCCCAG
CCCTCCAACAACCACCACGACCACCCTTCCACCAACCACCACTTCCAGCCCTCTAACAACTACTC
CTCTACCTCCATCAATAACTCCTCCTACATTTTCACCATTCTCAACGACAACCCCTACTACCCCA
TGCGTGCCTCTCTGCAATTGGACTGGCTGGCTGGATTCTGGAAAACCCAACTTTCACAAACCAGG
TGGAGACACAGAATTGATTGGAGACGTCTGTGGACCAGGCTGGGCAGCTAACATCTCTTGCAGAG
CCACCATGTATCCTGATGTTCCCATTGGACAGCTTGGACAAACAGTGGTGTGTGATGTCTCTGTG
GGGCTGATATGCAAAAATGAAGACCAAAAGCCAGGTGGGGTCATCCCTATGGCCTTCTGCCTCAA
CTACGAGATCAACGTTCAGTGCTGTGAGTGTGTCACCCAACCCACCACCATGACAACCACCACCA
CAGAGAACCCAACTCCGACACCAATCACCACCACCACTACGGTGACCCCAACCCCAACACCCACC
AGCACACAGAGTACAACACCAACACCCATCACCACCACCAATACGGTAACCCCAACCCCAACCCC
CACTGGCACACAGACCCCAACCCCGACACCCATCACCACCACCACCACTATGGTGACCCCAACAC
CAACAATCACCAGCACACAGACCCCAACCCCGACACCCATCACCACCACTACGGTGACCCCAACC
CCAACACCCACCAGCACACAGAGAACAACACCGACATCCATCACCACCACCACCACGGTGACCCC
AACCCCAACACCCACCGGCACACAGACCCCAACCACGACACCCATCACCACCACCACCACGGTGA
CCCCAACCCCAACACCCACCGGCACACAGACCCCAACAACGACACCCATCACCACCACCACCATG
GTGACCCCAACCCCAACACCCACTGGAACACAGACCCAAACCCCAACACCCATCACCACCACCAC
TACGGTGACCCCAACCCCTACACCCACCGGCACACAGACCCCAACATCGACACCCATCAGCACCA
CCACTACGGTGACCCCAACACCAACACCCACCGGCACACAGACCCCAACCCTGACACCCATCACC
ACCACCACTACGGTGACCCCAACCCCAACACCCACCGGCACACAGACCCCAACCACGACACCCAT
CACCACCACCACTACGGTGACCCCAACCCCAACACCCACCGGCACAAAGAGTACAACCCCGACAT
CCATCACCACCACCACTATGGTGACCCCAACCCCACCACCCACTGGCACACAGACCCCAACCACG
ACACCCATCACCACCACCACTACGGTGACCCCAACCCCAACACCCACCGGCACACAGACCCCAAC
CCCGACACCCATCACCACCACCACCACGGTGACCCCAACCCCAACACCCACCGGCACACAGACCC
CAACATCGACACCCATCACCACCAACACTACGGTGACCCCAACCCCAACACCAACCGGCACACCG
AGTACAACCCTGACACCCATCACCACCACCACTATGGTGACCCCAACCCCAACACCCACCGGCAC
ACAGACCCCAACATCGACACCCATCAGCACCACCACTACGGTGACCCCAACCTCAACACCCACCG
GCACACAGACCCCAACCCCGACACCCATCTCCACCACCACTACGGTGACCCCAACCCCGACACCC
ATCTCCACCACCACTACAGTGACCCCAACCCCAACACCCACCGGCACACAGACCCCAACCATGAC
ACCCATCACCACCACCACCACGGTGACCCCAACCCCAACACCCACCGGCACACAGACCCCAACAA
CGACACCCATCAGCACCACCACCACAGTGACCCCAACCCCAACACCCACCGGCACACAGACCCCA
ACATCGACACCCATCACCACCACCACTACGGTGACCCCAACCCCAACACCCACCGGCACACAGAC
CCCAACCACGACACCCATCACCACCACCACCACGGTGACCCCAACCCCAACACCCACCGGCACAC
AGAGTACAACCCTGACACCCATCACCACCACCACCACGGTGACACCAACCCCAACACCCACCGGC
ACACAGACCCCAACCCCGACACCCATCTCCACCACCACTACGGTGACCCCAACCCCAACACCCAC
CGGCACACAGACCCCAACCACGACACCCATCACCACCACCACCACGGTGACCCCAACCCCAACAC
CCACCGGCACACAGACCCCAACAACGACACCCATCAGCACCACCACCACGGTGACCCCAACCCCA
ACACCCACCGGCACACAGACCCCAACATCGACACCCATCACCACCACCACTACGGTGACCCCAAC
CCCAACACCCACCGGCACACAGACCCCAACCACGACACCCATCACCACCACCACCACGGTGACCC

CAACCCCAACACCCACTGGCACACAGGCCCCAACCCCAACAGCCATCACCACCACCACTACGGTG
ACCCCAACCCCAACACCCACCGGCACACAGACCCCAACAACGACACCCATCACCACCACCACCAT
GGTGACCCCAACCCCAACACCCACCGGCACACAGACCCCAACATCGACACCCATCACCACCACCA
CTACGGTGACCCCAACCCCAACACCCACCGGCACACAGACCCCAACCCCGACACCCATCTCCACC
ACCACTACGGTGACCCCAACCCCAACACCCACCGGCACACAGACCCCAACCATGACACCCATCAC
CACCACCACCACGGTGACCCCAACCCCAACACCCACCGGCACACAGACCCCAACAACGACACCCA
TCAGCACCACCACCACGGTGACCCCAACCCCAACACCCACCGGCACACAGACCCCAACATCGACA
CCCATCACCACCACCACTACGGTGACCCCAACCCCAACACCCACCGGCACACAGACCCCAACCCC
GACACCCATCACCACCACCACCACGGTGACCCCAACCCCAACACCCACCGGCACACAGACCCCAA
CATCGACACCCATCACCACCACCACTACGGTGACCCCAACCCCAACACCCACCGGCACACAGACC
CCAACCACGACACCCATCACCACCACCACCACGGTGACCCCAACCCCAACACCCACCGGCACACA
GAGTACAACCCTGACACCCATCACCACCACCACCACGGTGAACACCAACCCCCAACACCCACCGG
CACACAAAACCCCAACATCAACACCCATCACCACCCACCACTACGGTTGACCCCAACCCCCAAAA
CCCACCGGCACACAGACCCCAACCCCAACACCCATTCTCCACCACCAATAACGGGTGACCCCAAC
CCCAACAACCCACCGGCACACAGACCCCAACCATGACACCCATCACCACCACCACCACGGTGACC
CCAACCCCAACACCCACCGGCACACAGACCCCAACATCGACACCCATCACCACCACCACTACGGT
GACCCCAACCCCAACACCCACCGGCACACAGACCCCAACCATGACACCCATCACCACCACCACCA
CGGTGACCCCAACCCCAACACCCACTGGCACACAGGCCCCAACCCCAACAGCCATCACCACCACC
ACTACGGTGACCCCAACCCCAACACCCACCGGCACACAGACCCCAACCACGACACCCATCACCAC
CACCACCACGGTGACCCCAACCCCAACACCCACCGGCACACAGAGTACAACCCTGACACCCATCA
CCACCACCACCACGGTGACACCAACCCCAACACCCACCGGCACACAGACCCCAACCCCGACACCC
ATCTCCACCACCACTACGGTGACCCCAACCCCAACACCCACCGGCACACAGACCCCAACCATGAC
ACCCATCACCACCACCACCACGGTGACCCCAACCCCAACACCCACCGGCACACAGACCCCAACAA
CGACACCCATCAGCACCACCACCACGGTGACCCCAACCCCAACACCCACCGGCACACAGACCCCA
ACATCGACACCCATCACCACCACCACTACGGTGACCCCAACCCCAACACCCACCGGCACACAGAC
CCCAACCACGACACCCATCACCACCACCACCACGGTGACCCCAACCCCAACACCCACTGGCACAC
AGGCCCCAACCCCAACAGCCATCACCACCACCAGTACGGTGACCCCAACCCCAACACCCACCGGC
ACACAGACCCCAACCACGACACCCATCACCACCACCACTACGGTGACACCAACCCCAACACCCAC
CGGCACACAGTCCCCAACCCCAACAGCCATCACCACCACCACTACGGTGACCCCAACCCCAACAC
CCACCGGCACACAGACCCCAACATTGACGCCCATCACCACCACCACTACGGTGACCCCAACCCCA
ACACCCACCGGCACACAGACCCCAACCCCGACACCCATCTCCACCACCACTACGGTGACCCCAAC
CCCAACACCCACCGGCACACAGACCCCAACCACGACACCCATCACCACCACCACCACGGTGACCC
CAACCCCGACACCCACCGGCACACAGACCCCAACCACGGTACTCATCACCACCACCACTACGATG
ACCCCAACCCCAACACCCACCAGCACAAAGAGTACAACCGTGACACCCATCACCACCACAACTAC
GGTGACCGCAACCCCAACACCCACCGGCACACAGACCCCAACCATGATACCCATCAGCACCACCA
CTACGGTGACCCCAACCCCAACACCCACCACTGGAAGCACGGGGCCCCCCACCCACACAAGCACA
GCACCGATTGCTGAGTTGACCACATCCAATCCTCCGCCTGAGTCCTCAACCCCTCAGACCTCTCG
GTCCACCTCTTCCCCTCTCACGGAGTCAACCACCCTTCTGAGTACCCTACCACCTGCCATTGAGA
TGACCAGCACGGCCCCACCCTCCACACCCACGGCACCCACGACCACGAGCGGAGGCCACACACTG
TCTCCACCGCCCAGCACCACCACGTCCCCTCCAGgtaagcagagccgcttggttcctctggcctg
ggatgcttcttcctccccttgtgccgggcaggactgtcccaggaaggctcaaggcacgttctggg
cgcctctctgcccacgaagcttggtcactgtgtgggcagaagccactgacactggccagtgctgg
gcagtgaagccaaaggccattccgcttgcccataggacagccttctgaggagctgctgacaccgg
ccagtgctgggcagtggagcccttggctatcctgctcgcccataagacggccttcttcaggggcc

cactgctatgtgatgcggtgctgtgggagcccatcaaggctgggggcagagagaggctgccagt
gaggtgcctgcgggtccacctgcttctggctgcagcccctccttggggccttttcctggtggacg
gcatgccacagccagtgccttctggacgcctcttgctggccatcggcttggccagcaagctgtgt
tgctgccagagcaccaggtcacctgcaggctctcgtgacactcggctgtggtgatactggccttg
ccgctccaccctgcctggtgactctgagagcctgggaggtgggcacgaggccctggtcctccagt
tctgccacccggtcggctgtctggctcccttgcagctggggagtggcagttgggaccctgtggca
tctgagatgtgcaacgtctcagccctcactggtgtctcctgctctcacagGCACCCCCACTCGCG
GTACCACGACTGGGTCATCttcagcccccacccccagcactgtGCAGACGACCACCACCAGTGCC
TGGACCCCCACGCCGACCCCACTCTCCACACCCAGCATCATCAGGACCACAGGCCTGAGGCCCTA
CCCTTCCTCTGTGCTTATCTGCTGTGTCCTGAACGACACCTACTACGCACCAGgtactcaggctg
ttcacatcctgtgcttgggtggccgaggctggccccggcatgtaccaatgggtcaggtgccaggg
ctgagatcgcagtagaagcgtctcaggaggcagcagccgtcgagggtggctgtgtccagggcacg
gcttcccttgggtggcctctgtggggacctccgctgtggggacctccacggggtccagcggctag
ccctgcctccggctagccctgcctctggacggtgtgatcgtgggtctgtctcccttcgcagGTGA
GGAGGTGTACAACGGCACATACGGAGACACCTGTTATTTCGTCAACTGCTCACTGAGCTGTACGT
TGGAGTTCTATAACTGGTCCTGCCCATCCACGCCCTCCCCAACACCCACGCCCTCCAAGTCGACG
CCCACGCCTTCCAAGCCATCGTCCACGCCCTCCAAGCCGACGCCCGGCACCAAGCCCCCCGAGTG
CCCAGACTTTGATCCTCCCAGACAGgtcagtgggctgcaggcggctttgtccccatggcactctg
cgcagcatgtccgggcagctgaggccccaggcaccacttcctgctggtcgtctgagggccgaggc
ctccagcaacccttgggtgcagggtctgccgagccctccacattttcaccgtgccccgctgtgcc
tggcgaggtggctggctgcagtgaggtccgtggaagccacttcggcctccagcctcccggctcag
cacccgcccctcctgagcgcagaccaccccatcctgtgccggtcccccctgacgtcccttgcctcc
cgtccccagGAGAACGAGACTTGGTGGCTGTGCGACTGCTTCATGGCCACGTGCAAGTACAACAA
CACGGTGGAGATCGTGAAGGTGGAGTGTGAGCCGCCGCCCATGCCCACCTGCTCCAACGGCCTCC
AACCCGTGCGCGTCGAGGACCCCGACGGCTGCTGCTGGCACTGGGAGTGCGACTgtgagtccggg
gcccccaggccctccccgcatctcctgccctctccgtgggtgggggctgcagggcccgtctcccg
ggggcggaagggctgaggctccttgggcacagatcccactgaggtgttcgctgaggctgggtgac
ttctgagggtcttctcacagccctgcttttgcctcattgggtggggagggcctgggcaggtggag
ggcttgcctggtggagttagggctcctccctggaacaagggtgcttctgaggcaagagggggctg
agttgaagtttgaaccctggtccgtcctgcagaatgggccactgtgggtgcgccagggcaagtgc
agctcagacatccccgtgcccacgcacaggagtggggttttcaggccccagcttcctgctggctc
ttcctgactatgccccagcccagcccttgcacccgaccccggccgaggggcacaggtggcacggc
tcactccggctcccttgcagGCTACTGCACGGGCTGGGGCGACCCGCACTATGTCACCTTCGACG
GACTCTACTACAGCTACCAGGGCAACTGCACCTACGTGCTGGTGGAGGAGATCAGCCCCTCCGTG
GACAACTTCGGAGTTTACATCGACAACTACCACTGCGATCCCAACGACAAGGTGTCCTGCCCCCG
CACCCTCATCGTGCGCCACGAGACCCAGGAGGTGCTGATCAAGACCGTGCATATGATGCCCATGC
AGGTGCAGgtaggcacagcgtggccacaggaggctggcatggaggcgggtgctgacatgggcccc
aatgcaccctggttccccaggggccagaggactgggctgtgggggtgccaaggcatagcctctcc
tagagttgggctagaaggtaggatggggtgggcgactggctccgggacatatcagctcttcctgc
aggccctccaggtgtgtcctgggcccctcgagccctggcaccatgccacgctgggcacagtctct
gcagcagaagctgcctcctgaggacagagtcagggacagggctctgcacacccttggctgagatg
cccctacttgcaggggaatcattggttctgaggctcaggaggccccgggagcctgcgccgggctc
cacagtccccaggtgctcccaggagagctccttcactggctcacccatgggaccagggtctggtt
gggagcagtggagtggaagcaagaaagggggcaggaaagcggggtaggcagggccctctccctac

atgtgtaggtcagagagcaggcggggtggggcagccctggagctctcacaaggagaggaccgagg
cagctgcagctcccatggtgtgtcggccacagGTGCAGGTGAACAGGCAGGCGGTGGCACTGCCC
TACAAGAAGTACGGGCTGGAGGTGTACCAGTCTGGCATCAACTACGTGGTGGACATCCCCGAGCT
GGGTGTCCTCGTCTCCTACAATGGCCTGTCCTTCTCCGTCAGGCTGCCCTACCACCGGTTTGGCA
ACAACACCAAGGGCCAGTGTGgtgagttccgtgacccccatggcccccgaggcccccacggctcc
caccgtcccctgtgcccccatgtcctgccccagggcgggtggccaggccaggctgaggctgaggc
tgcgtgtaaacacccatgggcctggctgtgggcctcttgccccgctgctcggggctgctgtggcc
atcacccgggttcagtctctgtgaggagccaacaggagggggcctggcctggtctctgccctcgg
ccctggctggccggtcctgggcatctgggctggagaagggcagggcttaccctgtctgcaacgtg
gcctctctcactgatacagGCACCTGCACCAACACCACCTCCGACGACTGCATTCTGCCCAGCGG
GGAGATCGTCTCCAACTGTGAGGCTGCGGCTGACCAGTGGCTGGTGAACGACCCCTCCAAGCCAC
ACTGCCCCCACAGCAGCTCCACGACCAAGCGCCCGGCCGTCACTGTGCCCGGGGGCGGTAAAACG
ACCCCACACAAGGACTGCACCCCATCTCCCCTCTGCCAGCTCATCAAGGACAGgtgaccccgccc
aggcctgcctgtggccacgacaccaataagctgagggcctctgtgccccagcccccagctcttgc
aaagaggaaggaggcagcgcgtggggcctggcgctggggctgggaaggcacggagccgcggaacc
aggatcaggcgctaggtcgccgtggggtccaggacccaggcccttgggttccacggggctgagct
gctacgtgcggcctgtgcctttgctgaactccagtctctcctggctcccgggaaggtgcagggct
ggccgagtgtgaggcccggagtaaaccagtcaacccaggacagagctcagggctgatattgggag
ggcagatttgggctttgacagagagggggtgctcctaacgctggcagtcatgggggtcagcatc
ctgtccctggaagtataggggccaggtataggctgggtgtccatctgccagggttgctggagggg
gtcctgaagctgatgaccacatagacgtggtttctatctctgggagccgggctgcagagccacct
tgctcggccatcccttggtctgtccctgagctgtccccctggctggcctgtcccttgaccctcca
tcagccacaggcgcctctctggcgggtgccggactccaggaggacagtccgggcagagacgctgg
ggtagagagcaggggagaggcaggtgccacctgagtgtgacctgtgcctctccctgcgcagCCTG
TTTGCCCAGTGCCACGCACTGGTGCCCCCGCAGCACTACTACGATGCCTGCGTGTTCGACAGCTG
CTTCATGCCGGGCTCGAGCCTGGAGTGCGCCAGTCTGCAGGCCTACGCAGCCCTCTGTGCCCAGC
AGAACATCTGCCTCGACTGGCGGAACCACACGCATGGGGCCTGCTgtaagtgcccatctgcccct
gccctggagctgggggcctgcaggccagacgtggtctctaggctctgccaggtgctgtgcccagc
ctgaagctagacctagatgggctgcggccagggacgcagagatggcgggtgtgagaccagggctg
gggccatggggtggggaaggccaggctggaggggctgaggtgctggggcttctgccagcatcgct
aaatgcaactgggtgcccaccacccagctcgggacaacctcgagggtggaggttgatgcccaggc
agctggtcaccctcctccgtgtgtggggcactgggcagctgtcactcaaggggtccaggctcct
ccgcctgacatgaggcagccctctgacctctgcccatgtccctcagTGGTGGAGTGCCCATCTCA
CAGGGAGTACCAGGCCTGTGGCCCTGCAGAAGAGCCCACGTGCAAATCCAGgtatgttgtttgag
ggtccaccaggaccgtgggctcgccttctgcagtgcggagggtggcatcatctgggcatagcagt
cccacctgccagctccccagccccaccccacctgtctgacaatgccctcccgcccccagCTCCTC
CCAGCAGAACAACACAGTCCTGGTGGAAGGCTGCTTCTGTCCTGAGGGCACCATGAACTACGCTC
CTGGCTTTGATGTCTGCGTGAAGACCTGCGgtacgccacccactcacactgtcccctcctgcctc
cctcctgcctcctcctgggtgtccacggaggctgggaccaggacgctgaccaccccccacctctg
atccctgttgcacaaggactctgctaacacaacttgtctcctgggtgtccatggaggctgggacc
aggaggctgaccaccccccacccctgctccctgctgcacaaggactctgctaacacaacttgtttc
ttccctcttcctagGCTGTGTGGGACCTGACAATGTGCCCAGAGAGgtaggccccaccgtgttgc
tggggggatccttccacaaattctgaattctggggagtgaggggatggacatgaaaacctggagcct
caaagattgaggaatgaggtcatctaagtcctggatggctgagttggcatggacaccacccactc

acccacccatccttccacccacccactcatccacctgtgcacccatctacccactcacctacccc
tccatccttccacctacctagtcatcacccactcatctatgcaccccccacccacccactcatc
catccatccatccaccatccacctacccaaccatccacccatccatccaccatccatctaccatc
caccatccacccaaccatccaccatccatccatccacccatcatccatctaccatccacccaccc
acctatccatccatccatccaccatctgtctaccatccacccacccactcatccatccatccatc
caccatctgtctaccatccacccacccacctatccatccacccatccatccatccatccatccat
ccatccatccatccatccacccaccatctgtctaccatccacccacccacctatccacccatcca
cccacccatccatccacccaaccatccaccatccatccatccatccatccatccaccatccatct
accatccaccctcccatccatccacgcatccacccaaccatccatccatccatccaccatccacc
caccatccacccatttatccatccattctccctccctccattcaccacccattggtcatatgata
ctctgtctagaagctctgacatgacatcttggccacctctgtgctgcccatgcctcctacctgtg
gtagcagccatgtggatgattccttagctaaattctgtacaaacctgagaggcctgagtggagaa
tttgccacgtgccaagcccctgcttgtcgatgctggtgagcaggtaatggctttgtgatatcagt
gaatgagcagctactgtcctatcccagaacctgcctggtgtgctcagaagtgaggagggacatgg
ttttcccccaggatccctcagcactctgctcagggtggctgtttctccccgctgaccacagctgc
agctccggggctgtggtgaggtggggcctgcctggtgccacctgtcctctctactcacccttctt
tccctgcagTTTGGGGAGCACTTCGAGTTCGACTGCAAGAACTGTGTCTGCCTGGAGGGTGGAAG
TGGCATCATCTGCCAACCCAAGAGGTGCAGCCAGAAGCCCGTTACCCACTGCGTGGAAGACGGCA
CCTACCTCGCCACGGAGGTCAACCCTGCCGACACCTGCTGCAACATTACCGTCTGCAgtaaggcc
atcccctggggcccatgccacctctcaggggtgcacacatccctgtaggctgggctgcctgctgt
cccctccttggcaagtgaggaaacagctggcttgggggcctctgctgtgccccttgagagggctt
gggagggggccgctgggcccagtccaggcatccctgctgcagggcctgacctgggtggggagggg
acccttggaggtgctggaggcccgaccctgtgcagtggccccgggggctttgcctgggaggagcc
accctcacggccgcgtgcgcaccctgtcttcagagtgcaacaccagcctgtgcagtggccccggg
ggcttggcctgggaggagccaccctcacggccgcgtgcacaccctgtcttcagAGTGCAACACCA
GCCTGTGCAAAGAGAAGCCCTCCGTGTGCCCGCTGGGATTCGAAGTGAAGAGCAAGATGGTGCCT
GGAAGGTGCTGTCCTTTCTACTGGTGTGgtaagcagggctggtgggcagggcagggaggaggctg
ccgcccggggtggggtggctgtaaggggggttggctccctcctgggggtctcagattctggggaca
cagatggctgtacgcttggctgatgcacccaccccagccctgagcgctcgctccatccactgggt
gtgcaccgggagtgggggtctggccaggtggccgccccggggcagtctccaacgaacggccttct
ccgttctttctcccaagAGTCCAAGGGGGTGTGTGTTCACGGGAATGCTGAGTACCAGgtgagcc
ctgggctgggtgagagggaggaggggaggaggtcggctgcagcgtgggggtcctggcaggctgtt
gggctggctgggatgctggagaggcccctgcctcatgtctctccctgtgcccgaagCCCGGTTCT
CCAGTTTATTCCTCCAAGTGCCAGGACTGCGTGTGCACGGACAAGGTGGACAACAACACCCTGCT
CAACGTCATCGCCTGCACCCACGTGCCCTGCAACACCTCCTGCAGCCCTgtaagcggccaccctc
ctccttcagcctgcccttttccctcctcccagacaagcacccgggcccatgtctgcatcgtgacc
ctttctttcctcctttcaacgccaacctgtccctgtccccacctctccatcctgacacctgccca
gcctggggcctcctccaggtgggggggtctcggcagccctgcaggctttgtgtggtgtggggtac
agcctgggagttcagttgcagtggcgtgtctatgtgcgcagGGCTTCGAACTCATGGAGGCCCCC
GGGGAGTGCTGTAAGAAGTGTGAACAGACGCACTGTATCATCAAACGGCCCGACAACCAGCACGT
CATCCTGAAGgtaggtgtgcactgccggccccgacgcggccgggttgcttgagcccagggcaagg
cgcgggccacccaggatcccccagctgagtcctcccagtcctgggcgcagctgtgatgggcgccc
tggggctgccatgacaaatgagcaggcgtcttcagggcagaaagggattctcctggttctgcggc
ccagaaatccatagagcaaagggcctcagggctgtgctccctcggaggcgctaggcaaggacctt

tcccagcctctggtcactctaggtgccccttggctgtgaccacgaggtttccttccctgtgtctg
cctctcctctcccttttaaggatttaggcaccccaagcaggatgatctcatcttaggatccttca
cttaatgacaccttcaaagacccccttccaaggcaggtcacattcatagattcagagttagaac
acagacagacctttgagggttgtgtgggctccaggctggtgcctgatgtggggccccgcccatgt
cacttgtcctgtggccctgggcctcaccaggaagcctccccggccaggtgtctccagggtgtctt
cctggccgggctggggctgggcctgctgccctccctcaccagagctccctgccccacag<u>CCCGGG</u>
<u>GACTTCAAGAGCGACCCGAAGAACAACTGCACATTCTTCAGCTGCGTGAAGATCCACAACCAGCT</u>
<u>CATCTCGTCCGTCTCCAACATCACCTGCCCCAACTTTGATGCCAGCATTTGCATCCCG</u>gtgagtt
ggccacctggggcctggctgtgtgtactctgccgggagtgggggtgcctggtgttctggggggct
ggggccccagtgctgcgacagtgacctcgggcctggtctgagctgccgcaggaggctttgcctgg
ggctttctgcagcagctacccccgcccacggcatcgtgggaaggtgctctcatccccaggaatgt
ccgggggtcccgggctcattctcctttccctctag<u>GGCTCCATCACATTCATGCCCAATGGATGC</u>
<u>TGCAAGACCT</u>gtgagtacagggcacagcctgggggtaggcagggtgggggcacaagggctggtg
ccctcagccccgcctggggtggctggaggctggacaacggcctctgggtgggcagtgagggctgg
gggctgaggccgagcctggggaggggacgcagcgagggagagcctcctcgaagatgtggaggccc
tgccctaagccgctgcccgctctccccag<u>GCACCCCTCGCAATGAGACCAGGGTGCCCTGCTCCA</u>
<u>CCGTCCCCGTCACCACGGAGGTTTCGTACGCCGGCTGCACCAAGACCGTCCTCATGAATCATTGC</u>
<u>TCCGGGTCCTGCGGGACATTTGTCAT</u>gtgagtcccaggctgggagtgtgcctggagggggtggtg
gagaccccagggaggcgagaggccagcgctggccccggaaggtcacccctcactccgccctcccc
ccag<u>GTACTCGGCCAAGGCCCAGGCCCTGGACCACAGCTGCTCCTGCTGCAAAGAGGAGAAAACC</u>
<u>AGCCAGCGTGAGGTGGTCCTGAGCTGCCCCAATGGCGGCTCGCTGACACACACCTACACCCACAT</u>
<u>CGAGAGCTGCCAGTGCCAGGACACCGTCTGCGGGCTCCCCACCGGCACCTCCCGCCGGGCCCGGC</u>
<u>GCTCCCCTAGGCATCTGGGGAGCGGG</u>

## Sequence S7

Refseq genome DNA sequence of human MUC5AC: NC_000011.10 Homo sapiens chromosome 11, GRCh38.p14 Primary Assembly (1158000-1200699)

ATGAGTGTTGGCCGGAGGAAGCTGGCCCTGCTCTGGGCCCTGGCTCTCGCTCTGGCCTGCACCCG
GCATACAGgtacggcttggcccctggccgctctactggtcctgggtggtgcggtactgagtgggc
ctcaggcagctcagtctttgccctgggttccgggcaggctgcatgtgccatgaacggctcccagc
agcatagcccctgactgtggcctggccacgaacgagcagtttccccttgtggggttgggaaggga
tctctgggcttcgcggacctctgaggctgggccattccctgaggcagggaagtaggagctcagat
ctcgggctttccctcccggcccggatccctgcacctgtccccagaagccgacagcacctggccca
cagtatctccagctgctcatggcccctgctgggcctggtcggggcattagccccaggcacctgcc
cttgcacaccccacacatgcccagttcaccagctcacgttccctgtctgggcctcgatggggatc
tcctgcgggggaggtctcagcctctgagctgggatgtgatggtgaggcccacaagggaactgtt
tcacccccgccctgaggctaagagggtgatgggggaggctccaggtcctgtggtctggactgccc
cttgctgggccagcagttcccttcctctgcttctgagggacacctgggaccgggtctgctggtga
ccatgtgcccccatggggtaatgccggggtttggagggggggtgggtgctcctgggctcctggaac
agcagggcagggacctcagcactgccgagaccgccacatggcccacccaggtccctcgcccagcc
ctgtccccagcctctggccctctgccaggcagtctcagagcaagaagaaccctctcccggtgtct
ccgtgccaccctccactgtggcctagtcggactctgccccgccgtccctcgtctgtaccaccctc
atctcagaagcaggaattctgtcccggggctgctcccaggagagggcggagctggggctggggtc
tgcgggggcttagaaggtgggggcaggcctgggctgggactagataagtgaggctcaggccgtca
tctccgtctcccccacggggcttgcagatgctaacacccctccgcccactgacggatttgatgca
gtgggccccactggggccagagggtgtgagggcgagggggtctcccagcctggcctgaggaccc
ctatgccagttgcgggaactggaaagctggggctggggtgcaggcgaatcacagctttccctaag
acccctctctgcaggtcctgggctgggccgggcgcccctcccaccatgctggtgctgtgcggggc
tgtgcggggctgtgcggggctggggtccagtcccacgatgatggtgctgggcggggctgtgcggg
gttgtgtgggggtctggtcccccatgctggggtgctgtgcggggctgtacgaggctgtgaggg
gctgtgcgaggctggggtctggtcccaccatgctggttctgtgcggggctgtgcggggctgtgcg
ggctgtgcggggctgtgcggggctgtgcggggctgtgcggggctgtgcggggctggggtctggt
cccaccatgctggctctgtgcagggctgtgcggggctggcgtctggccccaccatgctggttctg
tgcggggctgtgtggggctgtgtggggctgtgcggggctggggtctggtcccaccatgctggctc
tatgcagggctgtgcggggctggggtctggtcccaccatgctggctctgtgcagggctgtgcagg
gctggcgtctggccccaccatgctggttctgtgcggggctgtgtggggctgtgtggggctgtgtg
gggctggggctggtcccaccatgctggctctgcgacgggctgtgcggggctgtgtggggctgggg
ctggtcccaccatgctggctctgtgcagggctgtgcggggctggggtctggtcccaccatgctgg
ctctgtgcagggctgtgcgaggctgtggtctggtcccactatgctggctctgtgcagggctgtgc
ggggctgggccgggctgaaatctggtgacattctgcacattagcacagtctctatggaccctgaa
gatcagcctcctgcagggtctgctgcttgtagggaggcagggtcctccaggtggtcttggggccg
acccctcccatgcctttgactgctccagcccctcagtgggatcactgtactggacaggggtccca
agcgtggcagcgtgggggccagggcctggcacgcttggcagtgagtgggaatgaatagataggga
tgccagatcggggcgaagggtcctgacgccacctgctccatggggccaggctgaggagatgctcc
tggagggtctgggctcagacttcagactcacatggacagaggcctcctaggacccccccaaccca
gcacagagagagcccttgccacgggccacccccacgcactgtggcctcccgtccctctggtgtcc

atgctgcatctgtggccgcagcctgagccccctcagccaccctgcatctgggctcagcccccctc
ctctttctgcagGCCATGCCCAGGATGGCTCCTCCGAATCCAGCTACAAGCACCACCCTGCCCTC
TCTCCTATCGCCCGGGGGCCCAGCGgtgagtctgagtgtccggcccccaccctaagcctgtcaga
ttccaccctccaccgtgtggcacggcccctagggccactggtcttagggtggctccccagctggc
cactgctgaggaccaaacctgggggacaggaggacacccccacatccatggccctggtcggtca
cactacctgcctctccttgggccagcccctgcagcgcttaggctctgaaggagctgcagggcgg
ggcagatttgctgtgcccatgtcccgtgggagccggtcaccctccggggatctggagctgggtcc
tgccttggtgtcccccccgttcacctcctgacgcttcagcaacacgggcatctcccttggcgccg
gccgtccccatgtggctcgtgctagcgggagcccgtggaggcccagcagccctggcagaggcagg
gcaggcaagggcagccagggcagggggcttcaggcaaactctcagattgcatttctccaaatcgg
gccagacaggcctaatctcagcacctgagagattgtcttgagccgctgatgcagactcaggttct
gaggcccatgggcatgtgagagctggcagcccctggggacttcttcctggtgacagccgggagga
atgaaaaagtcaccgaacgcagggtgtgtggtcacaagtacacaacgtgcagatggtgggtgggt
gggagggggcccaacacgcagcagaatccggagttggcttcggagccaggagctgggacctgctg
actctggaacgtctcctctggctgcggagcccccgccccacgtggggccgtgcgtgaatcctacc
agcccctgtctccgcagGGGTCCCGCTCCGTGGGGCGACTGTCTTCCCATCTCTGAGGACCATCC
CTGTGGTACGAGgtgagtggagcccggaggcctgggtggggaagggtcatagctttgctgagctc
cccgctcaggcctggaggtgccgggtggagaggggccccagctttccgggtgaacactgggtggg
tattggagccagagggcccagcatctccctgcacacgtttctgggacttcccagatgcaggaagg
acccctgggtgccccgtccagggcagcagcacttcctgcaggaccctggggaggggcaggaggta
cagggcagaggcagggggtgcagggcgaggatgagggcgacgcccccaaacaccatgctgcttcc
accgcagCCTCCAACCCGGCGCACAACGGGCGGGTGTGCAGCACCTGGGGCAGCTTCCACTACAA
GACCTTCGACGGCGACGTCTTCCGCTTCCCCGGCCTCTGCAACTACGTGTTCTCCGAGCACTGCG
GTGCCGCCTACGAGGATTTTAACATCCAGCTACGCCGCAGCCAGGAGTCAGCGGCCCCCACGCTG
AGCAGGGTCCTCATGAAGGTGGATGGCGTGGTCATCCAGCTGACCAAGGGCTCCGTCCTGGTCAA
CGGCCACCCgtgagtctgggttctgggatggtgggggccacgcggcgtgtggggtggcatttccg
ggtggttgcggggtttcgcggtcctgggagggatgtggatttctcaggaagcccctgagagcaga
gctggacatgggccctccctcgtcccccgagtggcccacctgcccctcctgggatccccaggctc
ttattctgccccttcctagctcctcgtgacccccgagctccagaggtcaatgtccccatcccaga
cgcgacttcacggtcacgatgaccgtgtcacatttgcgaccgcaggcatctgccctgcctggggt
ctctcctcactgcgctcccagcccctcagccctgccttcctccacagGGTCCTGCTGCCCTTCAG
CCAGTCTGGGGTCCTCATTCAGCAGAGCAGCAGCTACACCAAGGtggaggccaggctgggcctTG
TCCTCATGTGGAACCACGATGACAGCCTGCTGgtgaggctgggtgggggtgtcccggtgtgcaac
tccagccctcgagggccggcctgctcccacagcctctccggagagggtagaaggtgccctgggcc
cagtcagggtcagactccaccccacacagcaggcaagaacaggtgcccagacaccaatggtgtcc
cggggtctgtgcccccaggatggagactgctttcgggggtgtctcttccgtgggcctcggcccct
cctcggaaatctcaggctcgagcctcatctgtccatctgcccctggtggggatgggtgtctgatg
tctctccctttgcagCTGGAGCTGGACACCAAATACGCCAACAAGACCTGTGGGCTCTGTGGGGA
CTTCAACGGGATGCCCGTGGTCAGCGAGCTCCTCTCCCACAgtaaggccccacatcgccctcagc
cccttcctcagtgtcccctgggggctcagtgttgtgtgcacacacaccctctgacactccgggca
cacacatgcacagatacacggatgcagctgccctccctcctagcacagcacacacgtgcacacac
gcgatcccgcaacgccggcctgtctcaggagtgcaggcagggagcaaatcgcccattgggcccct
tgctctgtgtggctgctctgggtggcgacccctgactgcacgccctctcctgaaatgacagacct
gcctccttcttggtctttgagtctctgccaccaggcagtggccttgcaaatgtgacctgcaccag

ccaaggccccactccctgcctcccctgccaggcccatgaagccccatacaagccccgagtacag
ggtgtccctctgtggggaactgagtctgcctgggtgtccagcagcccttggcaaggcaggctcag
tgctgggggggctgggttctgtggactgggaggtgttcaggctccagccagcctggcaggaggag
accagggtcctgggtcagttgaggcctcagccggcggcaccctgtggcccggacaccaacctcct
gggctgtttctccccatcattccagacccctgagggtcccagcagcatgcatggggtggccaag
cgggtgcagtcaggacaactcaggcatccagatggggcaggagccaccgatggcccttctaaccc
caccccagggacccggccctggggctctgctgctcgggtgctgggctgacgggtaccgggacct
gcaggcagagcccgcctctgtgcttgccgcagACACCAAGCTGACACCCATGGAATTCGGGAACC
TGCAGAAGATGGACGACCCCACGGACCAGTGTCAGGACCCTGTCCCTGAACCCCCGAGGAACTGC
TCCACTGGCTTTgtaagccttggagggaacagagggcccagcaggttgagcaggaggggttgtga
gcctgggaaccggtccagatcccccaccgaggactcagacgggctgtggcctttgtcctagGGCA
TCTGTGAGGAGCTCCTGCACGGCCAGCTGTTCTCTGGCTGCGTGGCCCTGGTGGACGTCGGCAGC
TACCTGGAGGCTTGCAGGCAAGACCTCTGCTTCTGTGAAGACACCGACCTGCTCAGCTGCGTCTG
CCACACCCTTGCCGAGTACTCCCGGCAGTGCACCCATGCAGGGGGGTTGCCCCAGGACTGGCGGG
GCCCTGACTTCTGCCgtgagtgtcccagccccctgtcccccaacccctttggcagggagggcagg
ggcaggcagacgtgagccctctctctgcctcccgcagCCCAGAAGTGCCCCAACAACATGCAGTA
CCACGAGTGCCGCTCCCCCTGCGCAGACACCTGCTCCAACCAGGAGCACTCCCGGGCCTGTGAGG
ACCACTGTGTGGCCGGCTGCTTCTGCCCTGAGGgtgaggctcccccgcccctgggaaacacaggt
gcaccccgacaactagggggctgtgctcccatggccaagcctcggaagaaggaccccagtcctag
tgtcccgggcccctgaggctgactgaggcccctgtcctgggccgctgaggctggctgaggatcct
gtcctgggcccccctgaggctggctgaggcccctctcctgggcccctaaggctggttgagaatcct
gtcctgagcctcctgaggctggctgatgcccctgtcccgggcccctgaggctggctgaggcccct
gtcctgggcccctgaggctggctgaggatcctgtcctgggccccctgaggctggctgatgcccct
gtcccgggcccctgaggctggctgaggatcctgtcctgagcccccctgaggctggctgatgcccct
gtcccgggcccctgaggctggctgaggcccctgtcccgggcccctgaggctggctgaggatcctg
tcctgggcccccctgaggctggctgaggcccctgtcctgggcccctgaggctggctgaggcccctg
tcctggcccccctgaggctggctgaggcccctgtcccgggcccctgaggctggctgatgcccctgt
cccgggcccctgaggctggctgaggccccgtcctgggcccctggagctggctgaggccccagct
cgctgtggggccgccatgttgttcccctgcaacgcccactgcgtggacacagcaggcgcccgtca
taggcctgcctgaccccctgcagGGACGGTGCTTGACGACATCGGCCAGACCGGCTGTGTCCCTGT
GTCAAAGTGTGCCTGCGTCTACAACGGGGCTGCCTATGCCCCAGGGGCCACCTACTCCACAGACT
GCACCAACTGgtaggtcccagccccctccaggccaccaaggatgtgctatgggacagacctgct
gggggttgcaacccaggccggcaggctccctcgtctgggctacggtgtaggcaggcctggggtga
gacccggtcagcctcctgacgcggaggctggaggctggtctcctggggccggcacccacgtggca
ccatctcttgctctcagCACCTGCTCCGGAGGCCGGTGGAGCTGCCAGGAGGTTCCATGCCCGGG
TACCTGCTCTGTGCTTGGAGGTGCCCACTTCTCAACGTTTGACGGGAAGCAATACACGGTGCACG
GCGACTGCAGCTATGTGCTGACCAAGgtacggcctggctgcctggggtgctcgccggacagaggg
ggcccatggccagcctcccacaggctcccccagcttggctgcatgtcactgctgcccctggggtc
acccttgggggtccccgatgttgagacctcaaggaagcactccagctccccagcgctagtcctca
cagggccatgaaggctgcagatcagagcctccagcacccacccagcattgggcctcacccagcac
tgggccctctgcaccttgggctgtttatttaacagactttactcttgagaatagttttaggttaa
caaggaaattgagcacaaagtactcctgccccaacacacagtgtccccacgatgagaccctgcac
caaacacacagtctctcccagaggagaccctgcacccaacacacagtctccctatggtgagaccc
tgcacccaacacacagtctacccaagatgagaccctgcacccaacacacagtctccctatggtga

gaccctgcacccaacatacagtctctgcacgatgagaccctgcacccaacacacagtctcccacg
atgagaccctgcacccaacacacagtctccctatggtgagaccctgcacccgacacacagtctct
ccacaatgagaccctgcacccgacacacagtctctgcacgatgagagcctgcacccaacacacag
tctcccacgatgagaccctacacccaacacacagtctctccacgatgagaccctgcacccaacac
acagtctctccacgatgagaccctgcacccaacacacagtctccccaagatgagaccctgcaccc
gacacacagtctctgcacgatgagaccctgcacccaacacacagtctccccaagatgagaccctg
cacccaacacacagtctccccaagatgagaccctgcacccgacacacagtctctgcacgatgaga
ccctgcacccaacacacagtctccccaagatgagaccctgcacccaacacacagtctccccaaga
tgagaccctgcacccaacacacagtctcccacgatgagaccctgcacccaacacacagtttctcc
acgatgagaccctgcacccaacacacagtctccccaagatgagaccctgcacccgacacacagtc
tctgcacgatgagaccctgcacccaacacacagtctccccaagatgagaccctgcacccaacaca
cagtctccccaagatgagaccctgcacccgacacacagtctctgcacgatgagaccctgcaccca
acacacagtctccccaagatgagaccctgcacccaacacacagtctccccaagatgagaccctgc
acccaacacacagtctcccacgatgagaccctgcacccaacacacagtctccctatggtgagacc
ctgcacccaacacacagtctctgcacgatgagaccctgcacccaacacacagtctccccaagatg
agaccctgcacccaacacacagtctctcccagatgagactctgcacccaacacagtctccccaag
atgagaccttgcactcaacacatagtctcccccgatgagaccctgcacccaacacacagtctccc
acaatgagaccctgcacccaatacacagtttccctatgatgataccctgcgctggggtggaggct
gcactggtaccctcacctcccaggtcatggttgacatcagcgaccctcgtggtggtgaaattcca
cgtttaagacagatgtgcagtgtcttgttcctgccaggaagtttcacgcagacagctccactgcc
ctgaaaccccttggagctcctcacccctctctcgcccagcccctggcagctgctggcatttgcac
cttctgagtgctgtctttcccagagggtcctatagttggcgttgcagagtgtggcctcctcaggg
tgcatgcagatttctgtgtgtgcctgggtgtccacacctgggggcctcctagcacacctccctct
cggggactgggatggtggagtggggttttctagtgagggagaggagcacagccgggtggactggc
agggccaggtgggctgggcgttggatggagtgtgaggacccctggtgtgtcgtgttccgcagCCC
TGTGACAGCAGTGCCTTCACTGTACTGGCTGAGCTGCGCAGGTGCGGGCTGACGGACAGCGAGAC
CTGCCTGAAGAGCGTGACACTGAGCCTGGATGGGGCGCAGACGgtgagtggagcctggcagggca
aaccccgggaagagggaaggggcctgtctcttctgcaagtcacctctgcccaagcccttcatccc
tggcatgaacagcgagaggcggggaccctctggcaacactggctgtgtccattacttatgggtcc
acgtgactgtccctaaagggagaagctcagggccgggcctcccgggccgccatacctgacccaag
gggccccaggaaggagggactgggcgcacctgcggccagcatggcttctccactgaagtcaattt
atgtttagttaataaccttggttttcactgaagaatgaaaggcatggtcctcgcagaaaattcag
aaaatcaggcgagcacaaggccaccttgtaggagccgcagctgcagggaaaggcctcctaggcac
ctgcaggcacatttcacagcctccgcggggctgaggtgccgcaagcctgccccgcgctcacacat
ctgtctggctgccctcagGTGGTGGTGATCAAGGCCAGTGGGGAAGTGTTCCTGAACCAGATCTA
CACCCAGCTGCCCATCTCTGCAGgtgagggcagtggcttcttccccaccccggggctgcctgggg
tcccgccccacagcccccagcaaaacccttgtcctttgtgtccccagCCAACGTCACCATCTTCA
GACCCTCAACCTTCTTCATCATcgcccagaccagcctgggcctgcAGCTGAACCTGCAGCTGGTG
CCCACCATGCAGCTGTTCATGCAGCTGGCGCCCAAGCTCCGTGGGCAGACCTGCGgtaagagggc
tgccttctgggcttggagcccacccactctggccagggcgcatggtcctcaacctgcctaacccg
cccccagGTCTCTGTGGGAACTTCAACAGCATCCAGGCCGATGACTTCCGGACCCTCAGTGGGGT
GGTGGAGGCCACCGCTGCGGCCTTCTTCAACACCTTCAAGACCCAGGCCGCCTGCCCCAACATCA
GGAACAGCTTCGAGGACCCCTGCTCTCTGAGCGTGGAGAATGgtacgggtgtccacggctcgcct
ctgtgctggccgcctggcgctggttcacccgcttccatttggcactgcaggcagcgaggccggcc

ctgcgtgtgcctgtgagccgggtggggtggctcacgaaggggcccaaggacaggctcatggtggg
cgcccaacccagcttatgtggagcttcaggaatgtggggcatctgcttcagggtcaaagaaaggg
tacaagtctctgttggtcaacatccgccctgaccgcctacccctgcacagggtggagtggtgggg
acgtggggaatggattcacccactcacccacccgttcacccattcactcactcacccactcaccc
actcacccacctcactcgccgactcaaccattcactcacccattcgcccactcacctcactcact
cacccactcactcactcacctcactcactcacccactcactcactcacctcactcactcacccat
tcacccattcactcactcacccactcacccactcacccacctcactcgccgactcaaccattcac
tcacccattcgcccactcactcacctcactcactcacccactcactcactcacctcactcactca
cccattcacccattcactcactcacccactcacccactcactcacccattcacccattcacccac
tcactcacctcactcactgactcaactattcactcacccattcgctcactcactcacctcactca
ctcgcccactcacccactcacgctttcacccactcacctactcactcacccactcacccattcac
ccactcactcacccactcaccgactcacccattcacccactcacccactcactcacccactcacc
gactcaaccattcactcacccattcacccattcactcactcgcccactcacccactcacccattc
acccactcacctactcactcacccactcacccattcacccattcacccactcactcacccactca
ccgactcaaccattcactcacccattgcccactcactcacctcactcactcacccactcactcac
tcacctcactcactcacccactcacccattcacccattcactcactcacccactcacccattcac
ccattcactcactcacccactcacccattcacccattcactcactcacccactcacccattcacc
cattcactcactcacccactcacccattcacccattcactcactcacccactcacccactcactc
acccattcacccattcacccactcactcacctcactcactgactcaaccattcactcacccattt
gcccactcactcacctcactcacgcgcccactcacccactcacccattcacccactcacctactc
acccactcacccattcacccattcacccactcactcacccactcaccgactcacccattcaccca
ctcacccactcactcacccactcaccgactcaaccattcactcacccattcacccattcactcac
tcgcccactcacccactcacccactcacccactcacctactcactcactcactcacccattcacc
cactcacctactcactcacccactcacccattcacccattcacccactcactcacccactcacca
actcacccattcacccactcacccactcactcacccactcactgactcaaccattcactcaccca
ttcacccattcactcactcgcccactcacccactcacccactcacctattcactcactcactcac
ccactcacccattcacccattcacccactcactcacccactgactgactcaaccattcactcgcc
tattcgcccactcactcacctcactcactcacccactcactcacctcactcactcacccactcac
ccattcacccactcacctactcactcacccactcacccattcaccccactcacctactcactcac
ccactcacccattcacccattcacccactcactcacccactcaccgactcacccattcacccact
caccgactcacccattcacacactcaccgactcaaccattcactcacccattcgcccactcattc
acctcactcactcacccactcacccactcactcatccattcacccattcacacactcactcactt
actcactcactcaacttactcactcacccactcacccattcactcattcacccactcatccactc
acccactcactcacccactcactcacccattcacccactcactcacctactcactcagtcacctc
actcactcactcacgcattcactcactcactcacccactcacccactcacccattcactcaccca
ttcacccattcatccactcactcacctcactcactcactcaacttactcacccactcacccattc
acccattcacccccctcactcacccactcacccactcactcacccattcacccattcacccactca
ctcacccactcacccactcactcacctactcaaccacccactcacccactcacttacccattcac
ccactcacccactcactcacccattcacccactcacgaactcactcacccactcacccactcact
cactcacccattcacccactcacccactcactcacccattcacccactcacccactcactcaccc
actcatccattcactaactcacccattcacccactcacccactcaacactcactcacccactcac
ccattcacccactcacccactcatccattcactcactcacccactcacccactcaacactcactc
acccactcacccattcactcactcacccattcacccactcacccactcgctcacccattcaccca
ctcacctactcactcactcactcatccactcattcactcactcactcactcactcactcactcac

tcactcactcagtcattcacttcgccttcagagcttgggcgccagccgtggggaggtgggatccc
atttgctggttctggccttcttcctgcagcctgggaagccagggtccagtgagcccagtggaggg
gccagaagggccttggtttgtctgtccacagtggtggtcactagcgtccctggaaggcggcactg
tgagaggctgtgaggggctgggtgggacatgcaggaaacgagagaagcctcggttctggatgttg
taccctgcgtttgggggagcttttcctcggggagggcgccacaccacccaggctgagtgtgtagc
aggatgaagggcccaacatcagactgcccagagaagctggccacccaaaccccacaccagaggcc
aagctgggtgggatgagtgtgctgcctgatcttaatcctaaccctatccctcctctgtccacagA
GAAGTATGCTCAGCACTGGTGCTCGCAGCTGACCGATGCCGACGGCCCCTTCGGCCGGTGCCATG
CTGCCGTGAAGCCGGGAACCTACTACTCGgtaacatctgccgcctcttggcccggtggggctcca
gccactgagcctcacggctgcctccaggagggctgggagggcactggtgggctcagcaggcagga
ctaacctttctaggctgtgatgggcaccaggggctggaacccagtctgtctgtccagctccattc
cccacatccccacccacccattcactcattcacacattcaccactcacccacacactcacccatt
cacctactcactcacccgttcacccattcactcacccactcactcacccattcacccatttaccc
actcacccattcactcactcacccactcatccacccattcacccactcactcacccattcaccca
tttgcccccccactcactcacctattcacccatttacccactcacccattctctcactcgcccac
tcacccattcactcactcatccactcacccacccattcacccactcactcacccgttcacccatt
cgcccccccactcacccattcactcactcattcactaacttactcactcactcactcacccacta
gctctttcactcactcactcactcattcattcactcatatcttcactgactcattcactcattta
cccactcactaattccttcacctactcatctacttactcattcactcatccgctcactcatttac
tcactcattcacgaatttcttcactcatttactcatttactcactcactaattccttcacttatt
cattactcactcactcatccactcactcacccactcactcactcatccactcactcactcatcca
ctcacccactcactcactcattccttcattcattccttcactcactcattcactcatttactcac
tcactaattccttcacctactcattcattcatccactcatccactcactcactcatccactcact
catccactcactcatccactcactcgctcattccctcattcattccttcactcactcattcactc
atttactgactcactaattccttcacctactcattcatccactcactcacccactcactcatcca
ctcactcactcatccactcattactcatccactcactcatccactcactgattccctcattcatt
cctttactcattcactcatttactcactcactaattccttcacctactcattcattcacgcactc
atccactcactcactcactcatccactcactcatccactcactcactcatccactcactcgctca
ttccctcattcattccttcactcactcattcactcatttactgactcactaattccttcacctac
tcattcatccactcactcacccactcactcatccactcattactcatccactcacttactcatcc
actcactgattccctcattcattccttcactcactcattcactcatttactcactaattccttca
cctactcattcactcactcattcatccactcattcactcactcactcatccactcactcacttat
tccctcattcgttccttcactcattggttaaccatgtttatggcatttttcttgcacttccagcc
ctacactaggcccaggggctaaacttcaggggtttgttgcccacctggccccaggggccaaatga
gaattaggccaaggctgagctgatgtgctgtgtctgaggcaagctggtcttggctggacctgttg
ggtcacagagatgaaggatctggttctcccagtaacccctgctctgcacagtggttctgctccat
gacatgccaagcaattctgctgagggggcaggatgcctgtgaggactcacagaggggtcctgggg
gcaggaagacctgggatgggaggaccctggctgctggatgtgtggcctgcagggcgtgggggcc
accaggtgtgggctggggtctctgatgccccgatgaccccccttccctgcagAACTGCATGTTTGA
CACCTGCAACTGTGAGCGGAGCGAGGACTGCCTGTGCGCCGCGCTGTCCTCCTACGTGCACGCCT
GTGCCGCCAAGGGCGTGCAGCTCGGCGGCTGGAGGGACGGCGTCTGCAgtgagtgcctgccaagc
ccagcccctttccctcgctgggtggccgcgggtcttggggtgcccccagtgtgcacaggttgctc
taagggcccccgtcctctgtgctgggcttgagggcaaggagcgcccaggtcagtgtggccttgga
cccggccgaggaggggaggggagggtagccgagagggctgggctcactcactgctggctgcccac

tgccgggctgtgtgtcctgagaatcccctcttcctggcatcccgcagCGAAGCCTATGACCACTT
GCCCCAAGTCAATGACGTACCACTACCATGTCAGCACCTGCCAGCCCACCTGCCGCTCCCTGAGC
GAGGGGGACATCACCTGCAGTGTTGGCTTCATCCCCGTGGATGGCTGCATCTGTCCCAAGGGCAC
CTTCCTGGACGACACGGGCAAGTGTGTGCAGGCCAGCAACTGTCCCTGCTACCACAGAGGCTCCA
TGATCCCCAATGGGGAGTCGGTGCACGACAGCGGGGCTATCTGgtaagagctcccgctgtggact
gggggtccctcgtgtctcctggcccaggctgaggctctgaccaccatctcctcacagCACCTGC
ACACATGGGAAGCTGAGCTGCATCGGAGGCCAAGCCCCCGCCCCAGgtgagtgccagaaaggagg
cactgtggcccccagcctcctcatctctataagaaatcctgaaaaatggcttcagggttagcccc
accacaagtcagcggggctggtggttgtccatcagctgctcagtgtcaggcatggtttgtgccct
ctaggcagcaagggggaagaagggagggtgtggggcgcacatatgacactcacacccactcatgc
acatgctcacacacccactcatgcacacgctgacacgcccactcatgcacacacacccattcatg
cacacactcacactcatgcacatgctcacacacccactcatgcacacgcattcacaccctcatgc
acacgcactcacacccacttatgcaacactcacacacccactcatacacacgcactcacacccac
tcatgcacacacacccattcatactcatgcacacgctcacacacccactcatgcacacacaccgt
catgcacccacactcatgcacacgctcacacacccacatgcacacgcactcacacccacccactc
atgcacaccacacccactcatgcacacgcactcacacacccacttatggacacgctcacacatcc
actcatgcacatgcacacacacccacacatgcacacacacccactcatgcacacacactcatact
catgcacacacactcacacccagttatgcaacgctcacacccactcatggacacgctcacacacc
cactcatgcacatgcactcacacccactcatgcacacgcacccactcaatgtgcacgcacatggc
acagacacgtcctccctgaacacatgtttgaggcaccgcagcagcctgtggctggcccccctgacg
gcccctccctccccagTGTGTGCTGCGCCCATGGTGTTCTTTGACTGCCGAAATGCCACGCCCGG
GGACACAGGGGCTGGCTGTCAGAAGAGCTGCCACACACTGGACATGACCTGTgtaagtccctgag
gactccccaatgacagaccctccatctgcccctgcctgctaagggcgcctgtccccagggtgggc
agtggtgagcctctgacacattaggccatgggctgccccacgtcccagaggacccctgcccatgc
gctcccggactccctggtcctgtggtccctccactgtggtgtgggtgagctgtccctgggaggac
ctggcaggccccgtgccctgccccatcactcagtggtgtctgctggccctgcagTACAGCCCCCA
GTGTGTGCCTGGCTGCGTGTGCCCCGACGGGCTGGTGGCGGACGGCGAGGGCGGCTGCATCACTG
CGGAGGACTGCCCCTGCGTGCACAATGAGGCCAGCTACCGGGCCGGCCAGACCATCCGGGTGGGC
TGCAACACCTGgtatgccgggggctcaaagcccatgggggggtgtcaggcccaggaaaccagaggc
cctccttaaagacgggcgagcccccagcacaggggtcccgggaaaacgcagggcacagactcagg
gctggacgccaccagcagccccagccagggaggcccagtgggcgcgtgtctatggtgccaggtcc
ccccaggggtaggaaggctgcacccagtcaggcaggcaccctgtgtgtgctctagcctgaccccc
agatgtcccccagCACCTGTGACAGCAGGATGTGGCGGTGCACAGATGACCCCTGCCTGGCCACC
TGCGCCGTGTACGGGGACGGCCACTACCTCACCTTCGACGGACAGAGCTACAGCTTCAACGGAGA
CTGCGAGTACACGCTGGTGCAGgtgagccggcgcgtttggggtcctcacggcggcccccgtggcc
cgaagctgctcactgcctctctgcggctgccccagggtgcacacaggttgtccccgcctcatcct
ccttgcgggaaggagggcagggcctgcctggtccttgatggcctctgcttccccagAACCACTGT
GGCGGGAAAGACAGCACCCAGGACTCCTTTCGTGTTGTCACCGAGAACGTCCCCTGCGGCACCAC
AGGGACCACCTGCTCCAAGGCCATCAAGATTTTCCTGGGGgtgagcgaggctgggtggtcgcatg
ccctccaggaggctcccatggcagcgtctggtcaggtgggctggggttcttgctgggggccctga
gtgaccccttgccatgcagGGCTTCGAGCTGAAGCTAAGCCATGGGAAGGTGGAGGTGATCGGGA
CGGACGAGAGCCAGGAGGTGCCATACACCATCCGGCAGATGGGCATCTACCTGGTGGTGGACACC
GACATTGGCCTGGTGCTGCTGTGGGACAAGAAGACCAGCATCTTCATCAACCTCAGCCCCGAGTT
CAAGgtgagaccacgcccctcgtccaggccagggccggctggaaacctggaggtggggagcgtgg

aacaggtgggcagagacgagaggcacagagacacagagagaaacacagagatggaagcggggtgg
gaagtgggggggacggagccttggcaagggcagcgggtcaggagactccttggctgggtgtcagt
gttctggggctagagcaaaggcccgcaccctggcggatttgcccgcagcctggacactggaagtc
cagcagggcatccacagggccgaactctttcagaaggctctgggggctccttttggccccttgc
atctgccggggtccccagcgctccttggctgtggccacatcacttggatcgccgccccgtcttca
cgcagcctcctccctgtgactgggtctaagtttccctcttttataaggacaccaggcattggatt
caggacccactcattacctcagtctgcaagaccccatctcccagtgaggtgccatgcataggcgc
cggggtcaggacttaggcgggtccttcgaggccacgtcagcttctttttctccaggagggcttg
cctctggctccggccctcggtgctcagctgcactgcccggggcctccccaggtgtagctggaggt
gggaaggaggaagcagcacctgtggtcgctgttggccacctgggtgggaggaggcggccgcaggg
cagggtggccctgggtgggaggaggcggcctaggggcagggtggctctggccttctcggtttgct
gcacccacctccacctctcccggtgcctctgcagGGCAGGGTCTGCGGCCTGTGTGGGAACTTCG
ACGACATCGCCGTTAATGACTTTGCCACGCGGAGCCGGTCTGTGGTGGGGGACGTGCTGGAGTTT
GGGAACAGCTGGAAGCTCTCCCCCTCCTGCCCAGATGCCCTGGCGCCCAAGGACCCCTGCACGGC
CAACCCCTTCCGCAAGTCCTGGGCCCAGAAGCAGTGCAGCATCCTCCACGGCCCCACCTTCGCCG
CCTGCCACGCACACgtatgctggccgggggcgtttctctgggcccaaggggggtcatggtgaccc
aaggagcccccagaagggagaagggaatggggtctgggagacagctgccaaccaagggtgtgggc
tgctgggactggcgctggtatggactcgcctagaggggctgggcttccagctctgacacctgtca
gctatggtttcggggccctgggggggtgttaaccccaagggctgccgaggaagccccaggcactgt
ggatatccagatgggcccagccggccacttggggatgggcattcgccctccctgagctccactgg
aactcggccccggtcaatgtgccagcatgggcccggtccccaagagcccgcggggtggtgggggg
gtccctggaacctgaagccccgtctccctcagGTGGAGCCGGCCAGGTACTACGAGGCCTGCGTG
AACGACGCGTGCGCCTGCGACTCCGGGGGTGACTGCGAGTGCTTCTGCACGGCTGTGGCCGCCTA
CGCCCAGGCCTGCCATGAAGTAGGCCTGTGTGTGTCCTGGCGGACCCCGAGCATCTGCCgtgagt
gcgagtgggcacctggggaagaacaggaagcgccggcagcgtgtgccccaccacactcgccgctg
atgcgtggggtgtgtggggcgggtggggaaggtttccaaataaacagaaacacctgggcccagag
aggagggcgtggctgacagaggggtccctggcatggtagaacgttctgggcagaggggtcagcag
agctgcccagctctagagaggagggcgtggctgacagaggggtcgctggcatggtagaacattct
gggcagaggggtcagcagagttgcccagctctagagaggagggcgtggctgatggaggggtccct
ggcatggttgggcagagggttcagcagagctgcccagctctagggaggagggcgtggctgacgga
ggggtccctggcatggttgggcagagggttcagcagagctgcccagctctagggaggagggcgtg
gctgacgggtcgctggcgtggtagaacgttctgggcagtggggtcagcagagctgcccagctcta
gcccacacggagccttgccgggaacgggcactgctggatctgctctgactggagaccccactctg
gtggcagcctccgtggcaccctgataattgggggtcccgatctggcccaccctcccctgtcccc
atcttgtgatggccgggaggtgcaggggagggaagcggctttagaacagccctggggtgcggggc
ctctgcgagtgagttcctgggaccccaccgagcccttccttcctccctgcagCTCTGTTCTGCGA
CTACTACAACCCCGAAGGCCAGTGCGAGTGGCACTACCAGCCCTGCGGGGTGCCCTGCCTGCGCA
CCTGCCGGAACCCCCGTGGAGACTGCCTGCGGGACGTCCGGGGCCTGGAAGgtgggctggggccg
gtcggagggtggcctgggctccgccgcctgtggccttctcctggcccctcgaggagcctctgtgg
ccccagcttccagcacattctggtgctgtcggcgaggcccgctgcttgggggctgggcggagccc
tccagagcgaggtggacgacactcggtctggttgtgactctggcctctttggcccacagGCTGCT
ACCCCAAGTGCCCACCAGAGGCTCCCATCTTTGATGAGGACAAGATGCAGTGTGTGGCCACCTGC
CCAACCCCGCCTCTGCCACCACGGTGCCACGTCCATGGGAAGTCCtaccggccaggtgcagtggt
gcccTCGGACAAGAACTGCCAGTCCTGgtgagtcctttgggggaggaatatggagcctgcagca

tgcaggggaatttgaccacggcctgggctttgggtggggctgggagcggcagggctggggacctc
cccgttactggagttgaagcttgggtcctgtcctggcccaggcatctgtgacatcagcttctcag
ggaccagcacccccatgtcctgagtcccagaggcgggactgggtgtagggctcctctgggcagaa
aaccagcacaacctctggggagcaggctcgaggggctcagggggcggcagggggaagctgggccg
agatggagccctagggtccccaccggaaggatgcccattaaaatcaccccctggaaagtaatggct
ccactctggcacccacctcccctgacccccgactggaggggccccacgagggagggaccttgggc
tgacgctggagagacaactgctgggacccaggtggggccggcctcctgtcccccagtctgggggt
gcaatgcagttcccagggaactccaccccctgtcggagctgctccctgcccgccgttggtagcatg
ggatgcccgtggaaggcacacggccgcccccacgcatcggcctgcccttcttccttgtctccagC
CTTTGTACGGAGCGCGGCGTGGAGTGCACCTACAAAGCTGAGGgtgagcggccggcagcccctgg
ggctggggtccgggggtctctgtctgcgcccagcctctgacgggcctgggccctccgtccccata
gCCTGTGTCTGCACCTACAATGGACAGCGCTTCCACCCAGGGGACGTCATCTACCACACGACGGA
TGGCACGGGTGGCTGCATCTCCGCCCGCTGCGGGGCCAACGGCACCATTGAGAGGAGGGTCTACC
CCTGCAGCCCCACCACCCCTGTCCCCCCAACCACCTTCTCCTTCTCCACACCCCCGCTTGgtaag
gcaaatgtggccgaggagccccagggtgagcccccctcccactgcctggcacagctgcccttgctg
gacgctgaggtcacagcagctctgggcatgggcggcagcccctgaggatcaagaagggatcgagg
agcagggagtcgggctcggggatgacctgctgtttccccaccagccacctgcagtggggagggcc
tggcctccaagtcaccccagcaggcctggcgtccaagtcctgccctcagcagcccccgggcctgg
cctcattggggtgtcaggggtctggcagcccaggctggccccggggatgcatggctatcacttgc
ctcacaatgctcagggctcaggtaccccccatgtcctcgccctcttctgggggatgtctcagggcc
ccaaggcatcatctgagcttctctgagagtagaaagctctggaactcacgtgttctggggcacag
atgtgtggaccctgctcagggcatggcctgggagccacttccctaaaactcagtcggttcgcctg
gctcttcagaacctgtgccgctcatgtgggttctgagcagggaccgcgggtggggacaggaagac
agacctgctggcgcagctccagaccggtgacagagccgcagggagggggcgggcggcccccaagtg
cgggcctctcatgctcagctgccttctcttctgcccacagTCGTGAGCTCCACGCACACCCC
CAGCAATGGCCCAAGCAGCGCGCACACAGGCCCTCCGAGCAGCGCCTGGCCCACCAC
AGCAGGCACTTCTCCCAGGACGAGGCTGCCCACAGCCTCTGCCTCACTGCCGCCGGT
CTGTGGGGAAAAGTGCCTGTGGTCGCCATGGATGGATGTCAGCCGCCCTGGACGGGG
CACGGACAGCGGTGACTTCGACACACTGGAGAACCTCCGCGCCCATGGGTACCGGGT
GTGCGAATCACCCAGGTCGGTGGAGTGCCGAGCTGAGGACGCCCCCGGAGTGCCGCT
CCGAGCCCTGGGGCAGCGTGTGCAGTGCAGCCCGGATGTGGGGCTGACCTGTCGTAA
CAGGGAGCAGGCATCGGGGCTCTGCTACAACTACCAGATCAGGGTCCAGTGCTGCAC
GCCCCTACCCTGCTCCACCTCTAGCAGTCCAGCCCAGACCACTCCTCCAACTACCTC
CAAGACCACTGAAACCCGGGCCTCAGGCTCCTCAGCTCCCAGCAGCACACCTGGCAC
CGTGTCTCTCTCTACAGCCAGGACGACACCTGCCCCAGGTACCGCTACCTCTGTCAA
AAAAACTTTCTCAACTCCCAGCCCTCCGCCAGTGCCGGCAACATCAACATCATCCAT
GTCGACCACGGCCCCGGGGACCTCTGTGGTCTCCAGCAAGCCCACCCCCACGGAGCC
CAGCACATCCTCCTGCCTGCAGGAGCTTTGCACCTGGACCGAGTGGATCGATGGCAG
CTACCCTGCTCCTGGAATAAATGGTGGAGATTTTGACACATTTCAAAATTTGAGAGA
CGAAGGATACACATTCTGTGAAAGTCCTCGAAGCGTGCAGTGCCGGGCAGAGAGCTT
CCCCAACACGCCGCTGGCAGACCTGGGGCAGGACGTCATCTGCAGCCACACAGAGGG
GCTGATTTGCCTGAACAAGAACCAGCTCCCACCCATCTGCTACAACTATGAGATCCG
CATCCAGTGTTGCGAGACGGTGAACGTGTGCAGAGACATCACCAGACTGCCAAAGAC
CGTCGCAACGACACGGCCGACTCCACATCCAACCGGAGCTCAGACCCAGACCACCTT

CACCACACACATGCCCTCGGCCTCCACAGAGCAACCCACGGCAACCTCCAGGGGTGG
GCCCACAGCAACCAGCGTCACACAGGGCACCCACACCACACTAGTCACCAGAAACTG
TCATCCCCGGTGCACCTGGACAAAGTGGTTCGACGTGGACTTCCCGTCCCCCGGACC
CCATGGTGGAGACAAGGAAACCTACAACAACATCATCAGGAGTGGGGAAAAAATCTG
CCGCCGACCTGAGGAGATCACCAGGCTCCAGTGCCGAGCCAAGAGCCACCCAGAGGT
GAGCATCGAACACCTGGGCCAGGTGGTGCAGTGCAGCCGGGAAGAGGGCCTGGTGTG
CCGGAACCAGGACCAGCAGGGACCCTTCAAGATGTGCCTCAACTACGAGGTGCGTGT
GCTCTGCTGCGAGACCCCCAGAGGCTGCCACATGACCTCCACACCTGGCTCCACCTC
TAGCAGTCCAGCCCAGACCACTCCTTCAACAACCTCCAAGACCACTGAAACCCAGGC
CTCAGGCTCCTCAGCCCCCAGCAGCACACCTGGCACCGTGTCTCTCTCTACAGCCAG
GACGACACCTGCCCCAGGTACCGCTACCTCTGTCAAAAAAACTTTCTCAACTCCCAG
CCCTCCGCCAGTGCCGGCAACATCAACATCATCCATGTCGACCACGGCCCCGGGGAC
CTCTGTGGTCTCCAGCAAGCCCACCCCCACGGAGCCCAGCACATCCTCCTGCCTGCA
GGAGCTTTGCACCTGGACCGAGTGGATTGATGGCAGCTACCCTGCTCCTGGAATAAA
TGGTGGAGATTTTGACACATTTCAAAATTTGAGAGACGAAGGATACACATTCTGTGA
AAGTCCTCGAAGCGTGCAGTGCCGGGCAGAGAGCTTCCCCAACACGCCGCTGGCAGA
CCTGGGGCAGGACGTCATCTGCAGCCACACAGAGGGGCTGATTTGCCTGAACAAGAA
CCAGCTCCCACCCATCTGCTACAACTATGAGATCCGCATCCAGTGTTGCGAGACGGT
GAACGTGTGCAGAGACATCACCAGACCGCCAAAGACCGTCGCAACGACACGGCCGAC
TCCACATCCAACCGGAGCTCAGACCCAGACCACCTTCACCACACACATGCCCTCGGC
CTCCACAGAGCAACCCACGGCAACCTCCAGGGGTGGGCCCACAGCAACCAGCGTCAC
ACAGGGCACCCACACCACACCAGTCACCAGAAACTGTCATCCCCGGTGCACCTGGAC
AACGTGGTTCGACGTGGACTTCCCGTCCCCCGGACCCCATGGTGGAGACAAGGAAAC
CTACAACAACATCATCAGGAGTGGGGAAAAAATCTGCCGCCGACCTGAGGAGATCAC
CAGGCTCCAGTGCCGAGCCAAGAGCCACCCAGAGGTGAGCATCGAACACCTGGGCCA
GGTGGTGCAGTGCAGCCGGGAAGAGGGCCTGGTGTGCCGGAACCAGGACCAGCAGGG
ACCCTTCAAGATGTGCCTCAACTACGAGGTGCGTGTGCTCTGCTGCGAGACCCCCAA
AGGCTGCCCCGTGACCTCCACACCTGTGACAGCTCCTAGCACCCCTAGTGGGAGAGC
CACCAGCCCAACTCAGAGCACCTCCTCTTGGCAGAAATCCAGGACAACCACTTTGGT
GACAACCAGCACAACCTCCACTCCACAGACCAGTACAACCTATGCCCATACAACCAG
CACAACCTCTGCTCCTACAGCCAGAACAACCTCTGCTCCTACAACCAGAACAACCTC
TGCCTCTCCAGCCAGCACAACCTCTGGTCCTGGAAATACTCCCAGCCCTGTTCCTAC
CACCAGCACAATCTCTGCTCCTACAACTAGCATAACCTCTGCCCCTACAACCAGCAC
AACCTCTGCCCCTACAAGCAGCACAACCTCTGGTCCTGGAACTACTCCCAGCCCTGT
TCCTACCACCAGCATAACCTCTGCCCCTACAACCAGCACAACCTCTGCTCCTACAAC
CAGCACAACCTCTGCCCGTACAAGCAGCACAACCTCTGCCACTACCACCAGCAGAAT
CTCTGGTCCTGAAACTACTCCCAGCCCTGTTCCTACCACCAGCACAACCTCTGCCAC
TACAACCAGCACAACCTCAGCTCCTACAACCAGCACAACCTCTGCCCCTACAAGCAG
CACAACCTCCAGTCCACAGACCAGCACAACCTCGGCTCCTACAACCAGCACAACTTC
TGGTCCTGGAACTACCCCAAGCCCTGTTCCCACGACCAGCACAACCTCTGCCCCTAC
AACAAGAACAACTTCTGCTCCTAAAAGCAGCACAACCTCTGCCGCTACAACCAGCAC
AACCTCTGGTCCTGAAACTACTCCTAGACCTGTTCCTACCACCAGCACAACCTCTTC
TCCTACAACCAGCACAACCTCTGCTCCTACAACCAGCACAACCTCTGCTTCTACAAC
CAGCACAACCTCTGGTGCTGGAACTACTCCCAGCCCTGTTCCCACCACCAGCACAAC

CTCTGCTCCTACAACCAGCACAACCTCTGCCCCTATAAGCAGCACAACCTCTGCCAC
TACAACCAGCACAACCTCTGGTCCTGGAACTACTCCCAGCCCTGTTCCTACCACGAG
CACAACCTCTGCTCCTACAACCAGCACAACCTCTGGTCCTGGAACTACTCCCAGTGC
TGTTCCCACCACCAGCATAACCTCTGCACCTACAACCAGCACAAACTCTGCCCCTAT
AAGCAGCACAACCTCTGCCACTACAACCAGCAGAATCTCTGGTCCTGAAACTACTCC
CAGCCCTGTTCCTACCGCCAGCACAACCTCTGCTTCTACAACTAGCACAACCTCTGG
TCCTGGAACTACTCCCAGCCCTGTTCCTACCACCAGCACAATCTCTGTTCCTACCAC
CAGCACAACTTCTGCTTCTACAACCAGCACAACCTCTGCTTCTACAACCAGCACAAC
CTCTGGTCCTGGAACTACTCCCAGCCCTGTTCCCACCACCAGCACAACCTCTGCTCC
CACAACAAGCACAACCTCTGCCCCTACAACCAGCACAATCTCGGCCCCAACAACCAG
CACAACCTCTGCCACTACAACCAGCACGACCTCTGCTCCTACACCCAGAAGAACCTC
AGCCCCTACAACCAGCACAATCTCTGCCTCTACCACCAGCACAACCTCTGCGACTAC
AACCAGCACAACCTCTGCTACTACAACCAGCACAATCTCTGCCCCTACAACCAGCAC
AACTTTGTCTCCTACAACCAGCACAACCTCTACTACTATAACCAGCACAACTTCTGC
CCCTATAAGCAGCACAACTTCCACACCACAGACCAGCACAACTTCGGCTCCTACAAC
CAGCACAACTTCTGGTCCTGGAACTACTTCAAGCCCTGTTCCCACCACCAGCACAAC
CTCTGCCCCTACAACCAGCACAACCTCTGCCCCTACAACCAGAACAACCTCTGTCCC
TACAAGCAGCACAACCTCCACTGCTACAACCAGCACAACCTCTGGCCCTGGAACTAC
TCCCAGCCCTGTTCCCACCACCAGTACAACCTCTGCTCCTACAACCAGAACAACCTC
TGCTCCTACAACCAGCACAACCTCTGCCCCTACAACCAGCACAACCTCTGCCCCTAC
AAGCAGCACAACCTCAGCTACTACAACCAGCACAATCTCTGTTCCTACAACCAGCAC
AACTTCTGTTCCTGGAACTACTCCCAGCCCTGTTCCTACCACCAGCACAATCTCTGT
TCCTACCACCAGCACAACTTCTGCTTCTACAACCAGCACAACCTCTGGTCCTGGAAC
TACTCCCAGCCCTGTTCCCACCACCAGCACAACCTCTGCTCCCACAACAAGCACAAC
CTCTGCCCCTACAACCAGCACAATCTCGGCCCCAACAACCAGCACACCCTCTGCCCC
TACAACCAGCACAACCTTAGCTCCTACAACCAGCACAACCTCTGCCCCTACAACCAG
CACAACCTCTACCCCTACAAGCAGCACAACCTCCTCTCCACAGACCAGCACAACCTC
GGCTTCTACCACCAGCATAACTTCTGGTCCTGGAACTACCCCAAGCCCTGTTCCCAC
CACCAGCACAACCTCTGCTCCTACAACCAGCACAACCTCTGCCGCTACAACCAGCAC
AATCTCGGCCCCAACAACCAGCACAACGTCTGCTCCTACAACCAGCACAACCTCTGC
CTCTACAGCCAGCAAAACCTCTGGTCTTGGAACTACTCCCAGCCCTATTCCTACCAC
CAGCACAACCTCTCCTCCTACAACCAGCACAACTTCTGCCTCTACAGCCAGCAAAAC
CTCTGGTCCTGGAACCACTCCCAGCCCTGTTCCCACCACCAGCACAATCTTTGCTCC
TAGAACCAGCACCACTTCTGCCTCTACAACCAGCACAACCCCTGGTCCTGGAACCAC
TCCCAGCCCCGTTCCCACCACCAGCACAGCCTCTGTTTCAAAGACCAGCACAAGCCA
TGTTTCCATATCCAAGACAACCCACTCCCAACCAGTCACCAGAGACTGTCATCTCCG
GTGCACCTGGACCAAGTGGTTTGACATAGACTTCCCATCCCCTGGACCCCACGGCGG
GGACAAGGAAACCTACAACAACATCATCAGGAGTGGGGAAAAAATCTGCCGCCGACC
TGAGGAGATCACCAGGCTCCAGTGCCGAGCCGAGAGCCACCCGGAGGTGAGCATTGA
ACACCTGGGCCAGGTGGTGCAGTGCAGCCGTGAAGAGGGCCTGGTGTGCCGGAACCA
GGACCAGCAGGGACCCTTCAAGATGTGCCTCAACTACGAGGTGCGTGTGCTCTGCTG
CGAGACCCCTAAAGGTTGCCCCGTGACCTCCACACCTGTGACAGCTCCTAGCACCCC
TAGTGGGAGAGCCACCAGCCCAACTCAGAGCACTTCCTCTTGGCAGAAATCCAGGAC
AACCACTTTGGTGACAACCAGCACAACCTCCACTCCACAGACCAGCACAACCTCTGC

TCCTACAACCAGCACAACCTCTGCTCCCACAACCAGCACAACTTCTGCCCCTACAAC
CAGCACAACCTCCACTCCACAGACCAGCATATCCTCTGCCCCTACAAGCAGCACAAC
CTCGGCTCCTACAAGCAGCACAATCTCTGCTCGTACAACCAGCATAATCTCTGCCCC
TACAACCAGCACAACCTCTTCCCCTACAACCAGCACAACCTCTGCTACTACAACCAG
CACAACCTCTGCCCCTACAAGCAGCACAACCTCCACTCCACAGACCAGCAAAACCTC
AGCTGCTACAAGCAGCACAACCTCCGGTTCTGGAACTACTCCCAGCCCTGTTACCAC
CACCAGCACAGCCTCTGTTTCAAAGACCAGCACAAGCCATGTTTCTGTATCCAAGAC
AACCCACTCCCAACCAGTCACCAGAGACTGTCATCCCCGGTGCACCTGGACCAAATG
GTTTGATGTGGACTTTCCATCCCCTGGACCCCACGGTGGGGACAAGGAAACCTACAA
CAACATCATCAGGAGTGGGGAAAAAATCTGCCGCCGACCTGAGGAGATCACCAGGCT
CCAGTGCCGAGCCAAGAGCCACCCGGAGGTGAGCATCGAACACCTGGGCCAGGTGGT
GCAGTGCAGCCGCGAAGAGGGCCTGGTGTGCCGGAACCAGGACCAGCAGGGACCCTT
CAAGATGTGCCTCAACTACGAGGTGCGTGTGCTTTGCTGCGAGACCCCCAAAGGCTG
CCCCGTGACCTCCACATCTGTGACAGCTCCTAGCACCCCTAGTGGGAGAGCCACCAG
CCCAACTCAGAGCACCTCCTCTTGGCAGAAATCCAGGACAACCACTTTGGTGACAAG
CAGCATAACCTCCACTACACAGACCAGCACAACCTCTGCCCCTACAACTAGCACAAC
CCCTGCTTCTATACCCAGCACAACCTCTGCCCCAACAACCAGCACAACCTCTGCTCC
CACAACGAGCACAACTTCTGCCCCTACAACCAGCACAACCTCCACTCCACAGACCAC
CACATCCTCTGCCCCTACAAGCAGCACAACCTCGGCTCCTACCACCAGCACAATCTC
TGCCCCTACAACCAGCACAATCTCTGCCCCTACAACCAGCACAACCTCTGCTCCCAC
AGCCAGCACAACGTCAGCTCCTACGAGCACTTCCTCGGCTCCTACAACCAACACAAC
CTCTGCCCCTACAACTAGCACTACCTCTGCTCCCATAACCAGCACAATCTCTGCCCC
TACAACCAGCACAACCTCCACTCCACAGACCAGCACAATCTCTTCCCCTACAACCAG
CACAACCTCCACTCCGCAGACCAGCACAACCTCTTCCCCTACAACTAGCACAACCTC
AGCTCCTACAACCAGCACAACTTCTGCCCCTACAACCAGCACAACCTCCACTCCACA
GACCAGCATATCCTCTGCCCCTACAAGCAGCACAACCTCTGCTCCTACAGCCAGCAC
AATCTCTGCCCCTACAACCAGCACAACCTCTTTCCATACAACCAGCACAACCTCTCC
CCCTACAAGCAGCACAAGCTCCACTCCACAGACCAGCAAAACCTCAGCTGCTACAAG
CAGCACAACCTCCGGTTCTGGAACTACTCCCAGCCCCGTTCCCACCACCAGCACAGC
CTCTGTTTCAAAGACCAGCACAAGCCATGTTTCTGTATCCAAGACAACCCACTCCCA
ACCAGTCACCAGAGACTGTCATCCCCGGTGCACCTGGACCAAGTGGTTTGACGTGGA
CTTTCCATCCCCTGGACCCCACGGTGGGGACAAGGAAACCTACAACAACATCATCAG
GAGTGGGGAAAAAATCTGCCGCCGACCTGAGGAGATCACCAGGCTCCAGTGCCGAGC
CGAGAGCCACCCGGAGGTGAGCATCGAACACCTGGGCCAGGTGGTGCAGTGCAGCCG
GGAAGAGGGCCTGGTGTGCCGGAACCAGGACCAGCAGGGACCCTTCAAGATGTGCCT
CAACTACGAGGTGCGTGTGCTCTGCTGCGAGACCCCCAAAGGCTGCCCCGTGACCTC
CACACCTGTGACAGCTCCTAGCACCCCTAGTGGGAGAGCCACCAGCCCAACTCAGAG
CACTTCCTCTTGGCAGAAATCCAGGACAACCACTTTGGTGACAACCAGCACAACCTC
CACTCCACAGACCAGCACAACCTCTGCCCCTACAACCAGCACAATCCCTGCTTCTAC
ACCCAGCACAACCTCTGCCCCTACAACCAGCACAACCTCTGCCCCTACAACCAGCAC
GACCTCAGCTCCTACACACAGAACGACTTCTGGTCCTACAACCAGCACAACCTTGGC
TCCTACAACCAGCACAACCTCTGCTCCAACAACCAGCACAAACTCTGCTCCTACAAC
CAGCACAATCTCTGCCTCTACAACCAGCACAATCTCTGCCCCTACAACCAGCACAAT
CTCTTCCCCTACAAGCAGCACAACCTCCACTCCACAGACCAGCAAAACCTCAGCTGC

TACAAGCAGCACAACCTCCGGTTCTGGAACTACTCCAAGCCCTGTTCCCACCACCAG
CACAACCTCTGCCTCTACAACCAGCACAACTTCTGCTCCTACAACCAGCACAACCTC
TGGTCCTGGAACTACTCCAAGCCCTGTTCCCAGCACCAGTACAACCTCTGCTGCTAC
AACCAGCACAACCTCTGCTCCTACAACCAGAACAACATCTGCTCCTACAAGCAGCAT
GACCTCTGGTCCTGGAACTACTCCCAGCCCTGTTCCCACCACCAGCACAACCTCTGC
TCCTACAACTAGCACAACCTCTGGTCCTGGAACTACTCCCAGCCCTGTTCCCACCAC
CAGCACAACCTCTGCTCCTATAACCAGCACAACCTCTGGTCCTGGAAGTACTCCCAG
CCCTGTTCCCACCACCAGCACAACCTCTGCTCCTACAACCAGCACAACCTCTGCCTC
TACAGCCAGCACAACCTCTGGTCCTGGAACTACTCCCAGCCCTGTTCCCACCACCAG
CACAACCTCTGCTCCTACAACCAGAACAACCTCTGCCTCTACAGCCAGCACAACCTC
TGGTCCTGGAAGTACTCCCAGCCCTGTTCCCACCACCAGCACAACCTCTGCTCCTAC
AACCAGAACAACCCCTGCCTCTACAGCCAGCACAACCTCTGGTCCTGGAACTACTCC
CAGCCCTGTTCCCACCACAAGCACAACCTCTGCTTCTACAACCAGCACAATCTCTCT
CCCTACAACCAGCACAACCTCTGCTCCTATAACCAGCATGACCTCTGGTCCTGGAAC
TACTCCCAGCCCTGTTCCCACCACCAGCACAACCTCTGCTCCTACAACCAGCACAAC
CTCTGCCTCTACAGCCAGCACAACCTCTGGTCCTGGAACTACTCCCAGCCCTGTTCC
CACCACCAGCACAACCTCTGCTCCTACAACCAGCACAACCTCTGCCTCTACAGCCAG
CACAACCTCTGGTCCTGGAACTTCTCTCAGCCCTGTTCCCACCACGAGCACAACCTC
TGCTCCTACAACTAGCACAACCTCTGGTCCTGGAACTACTCCCAGCCCTGTTCCCAC
CACCAGCACAACCTCTGCTCCTACAACCAGCACGACCTCTGGTCCTGGAACTACTCC
CAGCCCCGTTCCCACCACCAGCACAACCCCTGTTTCAAAGACCAGCACAAGCCATCT
TTCTGTATCCAAGACAACCCACTCCCAACCAGTCACCAGTGACTGTCATCCTCTGTG
CGCCTGGACAAAGTGGTTCGACGTGGACTTCCCATCCCCTGGACCCCACGGCGGGGA
CAAGGAAACCTACAACAACATCATCAGGAGTGGGGAAAAAATCTGCCGCCGACCTGA
GGAGATCACCAGGCTCCAGTGCCGAGCCGAGAGCCACCCGGAGGTGAACATTGAACA
CCTGGGTCAGGTGGTGCAGTGCAGCCGTGAAGAGGGCCTGGTGTGCCGGAACCAGGA
CCAGCAGGGACCCTTCAAGATGTGCCTCAACTACGAGGTGCGCGTGCTCTGCTGCGA
GACCCCCAGAGGCTGCCCGGTGACCTCTGTGACCCCATATGGGACTTCTCCTACCAA
TGCTCTGTATCCTTCCCTGTCTACTTCCATGGTATCCGCCTCCGTGGCATCCACCTC
TGTGGCATCCAGCTCTGTGGCATCCAGCTCTGTGGCTTACTCCACCCAAACCTGCTT
CTGCAACGTGGCTGACCGGCTCTACCCTGCAGgttcgtgagtgtttctggtgcaattgttt
ctgagctcaccctggtcagtttttatccaggaacgccaagctgtgatgatgataggagtctctg
ctctttgtggcacaggctcattgtcacagagtggctgctggcattctctgaaatttttcccatt
acacaggtggtagaaagtgcccctctggctctgggagtttttgcttctcctttgagcaggactc
cactaaaggctgccatgtcccttcctcttacagGATCCACCATATACCGCCACAGAGACCTCGCT
GGCCATTGCTATTATGCCCTGTGTAGCCAGGACTGCCAAGTGGTCAGAGGGGTTGACAGTGACTG
TCCGTCCACCACgctgcctcctgccccagccacgTCCCCTTCAATATCCACCTCCGAGCCCGTCA
CTGAGCTGGGATGCCCAAATGCGGTTCCCCCCAGAAAGgtaacccccctacttctcacccttctga
aggctcaggggctcctacagggaacttgaatgtctttgcaattagtcttcagctgcaaagtcttg
agaaggtcactggcgctagtggaggcagcccggccgtcaggagagggccatgctgtagcccggcg
tctctgatcaccctgggcttcccaggcacgtgctttccagacactgggtgtgtggggccgaaggc
tgaggctgtgctgaggacagccagggccagagcaggtggatgaggctgcacagccagggagtgtg
gcagagccagggaggagccactcccacaccagaggcccctgcccgggccagctcggtgtctgggg
tgggtgctgagggtggggcaggaaggaaactggggcacagccaccctcccctgtccccacacggg

actctcgggactgtgaccccgagaaccaaggggtgccccggagatcgggagaggccggaccctgc
aggtctctgtgcttctgcagAAAGGTGAGACCTGGGCCACACCCAACTGCTCCGAGGCCACCTGT
GAGGGCAACAACGTCATCTCCCTGCGCCCGCGCACGTGCCCGAGGGTGGAGAAGCCCACTTGTGC
CAACGGCTACCCGGCTGTGAAGGTGGCTGACCAAGATGGCTGCTGCCATCACTACCAGTGCCAGT
gtgagtggagcgccgagcgggacccagggcagccggggcagccactccccacccaggtggaaatg
ggcggggcagaggagggggcttggctgaagcagaaaagaattgggtgggacagcaggaaggactt
cccagcatcaaggcggggccgcatggggccgagcctccccgcttaggggtgctgagtgaatccct
gtgagcctcggagcagcctggctggagaaggtggaggagtctgtccctgcctcgctccagccttg
tcaagcgcccgctggatggagctctgtgcggacgctggccacgtgtgttctgatgacgtgagggt
cttgtgcgccctgtgagatgagacggtgggggtcagggacagatgtgacctgttgggaggaaag
ccccagggccatggtgccaccacccgagccacccgtaaggctgcccctggggcctggcagGTGTG
TGCAGCGGCTGGGGTGACCCCCACTACATCACCTTCGACGGCACCTACTACACCTTCCTGGACAA
CTGCACGTACGTGCTGGTGCAGCAGATTGTGCCCGTGTATGGCCACTTCCGCGTGCTCGTCGACA
ACTACTTCTGCGGTGCGGAGGACGGGCTCTCCTGCCCGAGGTCCATCATCCTGGAGTACCACCAG
GACCGCGTGGTGCTGACCCGCAAGCCAGTCCACGGGGTGATGACAAACGAGgtgggggcgcgccc
ggtgtgccgcggagggggtgggggacgcggctttcccggcaagagcctgagcagcggctgaccgc
ccgcccgcctgccttctgacttcccgtcgaccacgccctgcgtccagATCATCTTCAACAACAAG
GTGGTCAGCCCCGGCTTCCGGAAAAACGGCATCGTGGTCTCGCGCATCGGCGTCAAGATGTACGC
GACCATCCCGGAGCTGGGAGTCCAGGTCATGTTCTCCGGCCTCATCTTCTCCGTGGAGGTGCCCT
TCAGCAAGTTTGCCAACAACACCGAGGGCCAGTGCGgtgaggccacagggctcccgggcatcgtc
tggcattcgcgggggcgggggtgccgggcaggggcgaggccaccacgtgccgcgtgtgccggtgt
ctctgctttctggctgctctgctgagtgcaggccacaggcatgaggcttacgcctgccggtacct
gcagctccccagtaccatagggactgtcccagggttgttctcgggggacagtgaggcacaggcag
ggcccgctgcagttcaccaagttgtgaccctgagacaccagggtcctgtcccccagctgggggtg
gggccagccggctctgctgtccagcagcctgacccccaccgcgtctgcccagGCACTTGCACCAA
CGACAGGAAGGATGAGTGCCGCACGCCTAGGGGGACGGTGGTCGCTTCCTGCTCCGAGATGTCCG
GCCTCTGGAACGTGAGCATACCCGACCAGCCAGCCTGCCACCGGCCTCACCCGACGCCCACCACG
GTCGGGCCCACCACAGTTGGGTCTACCACGGTCGGGCCCACCACAGTTGGGTCTACCACGGTCGG
GCCCACCACACCGCCTGCTCCGTGCCTGCCATCACCCATCTGCCAGCTGATTCTGAGCAAgtgag
acttgggtgcaagggagggagggtcagtgtggccgccccacctcccacccttccaagggctggag
gggaagaagcgcattttcagggggctcctgagaggaaacgagagctgctggtaccacagaccaag
gaggggtgggtgttggggagaagtgggtgccggggaggggtgggcagtggggaggggcgggtgcc
gggaaggggtggttgtggctggaatgctgccaaggggtcaccagggttggccggctgggccgtct
ctgaggctctctgctggctcaggtgtatggacgggggcatcggtcaccacccccaggcagcttcc
aggtttgcagggcaggggctgggggggccaggaagaagaacttgcagaaataaatggatgatgcc
atctggacccccacccccgcccccaagcacacagaatgtgtggaaggagcccgtccggggatac
aggagggcggccacacaccagtggctgctctgggactcgcctcgcctggagactgccctgaagcc
tgggagcctgacgtggagcaggcaggtggccggagaggctgcacccagcaccctgcccatccctc
ccacagGGTCTTTGAGCCGTGCCACACTGTGATCCCCCCACTGCTGTTCTATGAGGGCTGCGTCT
TTGACCGGTGCCACATGACGGACCTGGATGTGGTGTGCTCCAGCCTGGAGCTGTACGCGGCACTC
TGTGCGTCCCACGACATCTGCATCGATTGGAGAGGCCGGACCGGCCACATGTGCCgtgagtgcca
ccactgtcctcagggtcccaagtcgcttgtgaggggcacaggcacgccggacggaccaacagggt
gggctcgggcagtcaggggggggacctggaggaggagggggcagccccagggcagagtgcacccag
ttccctggagagaagggagggcgtccacgtctgggagcccgtggcggggcactggggtgtgggag

gccgtagccaggcccaggcccacaggtggctgcgggcagctccggagcagatgttggtgcccagc
ggcccgcgttgctctgggtgggtgccctcccaccctctcaggtgtggcttcccctccccacagCA
TTCACCTGCCCAGCCGACAAGGTGTACCAGCCCTGCGGCCCGAGCAACCCCTCCTACTGCTACGG
GAATGACAGCGCCAGCCTCGGgtaggcaccctccctcctggccctgccatgggctgctggagcca
cccagtccccagcctcccgctgcatctcccactcccaggctggggtccttctcaccggagggaaa
aaggagacccaccaaccctatgctctctacagGGCTCTGCCGGAGGCCGGCCCCATCACCGAAGG
CTGCTTCTGTCCGGAGGGCATGACCCTCTTCAGCACCAGTGCCCAAGTCTGCGTGCCCACGGGCT
GCCCCAgtacgtgccccaggccggggctggggggtgtggcaggactgggcctgtgactgttggcc
aggtcctggggtctaccctggccccaatatgggaccctgcctctcgcccctattgtggtggctga
cccccagtaacctcagatctctctccttccagGGTGTCTGGGGCCCCACGGAGAGCCGGTGAAGg
tgagtggaaggcatggcccaagagggcactggggtccaagagcccgaggagggaggctcttgaaa
caccggccccagaaatggtcaggtggggctcaggggtcggggtgtcctctctgtgtcagaggccc
agagaaagggctgcgggaggagccgggctcccagaaagactcgggatctcccttcccaggtaccc
actctgtccgtattggggacggtgccggggcgtctgtcctggtgttgctctgtgggtgggtgctg
gccccgaggctggctgtgctcttcccttccttgggacgccccttctgcccagccaagactgtgga
ggtgtgaggtgggggcgcaggccagtcaccccacagcctgcccaccttgcagagctttgtgccc
atccctgggccctaccctgcacttttgaagccctactcctgctgggtcctcagtgcaggccacac
ggcctccacacctggctgccccggcactgcatgagccggggtggctgctgcacccctgggtggag
ccgctgcggacgctggacctcagtccccttccttgcagGTGGGCCACACCGTCGGCATGGACTGC
CAGGAGTGCACGTGTGAGGCGGCCACGTGGACGCTGACCTGCCGACCCAAGCTCTGCCCGCTGCC
CCCTGCCTGCCCCCTGCCCGGCTTCGTGCCTGTGCCTGCAGCCCCACAGGCCGGCCAGTGCTGCC
CCCAGTACAGCTGCGgtaagccctttgctgggtgaggggcatggtgtggcaggcaggtgacccgg
agcccactcggcccggactttgctgctgccttggggcgtgcacctggggacagtgcctacgaggg
cgtcccctcctccgcttccgcaacagcctcatctggagacccccgagcgggctgtctaggcggtc
cgcaatcctagagaccccaggggggtgggccttcgggtgggggcgggggacagactcctaattgcc
tcactcccgcccccgcagCCTGCAACACCAGCCGCTGCCCCGCGCCCGTGGGCTGTCCTGAGGGC
GCCCGCGCGATCCCGACCTACCAGGAGGGGGCCTGCTGCCCAGTCCAAAACTGCAgtgagtggcc
tggaccaggccctgtcaggggccgtgggctggggtctccacctgggattttggggggccataacc
agatgccagtgcggcttgtccactgcgggtctgtggctctggactgagccttccgcagagatgag
gctggacaaaccccagtggcgagcccgggaatgaggcgcccaggaggctgcaggggcgggaatgc
tgagggtgaggggagagtgggcggcggggggtgcagctgcttgtcttctcgtgggcagGCTGGAC
AGTGTGCAGCATCAACGGGACCCTGTACCAGgtaagagccacggagctcagacccccctcagccat
agggacggagcttcccactgaccctgaggcccaggtagactttggagcaactgccaactccggcc
gaggccagggactcgagtctctgcagacacagcccactatcaagtgtggctgaggcccgaggtcg
gccccaggtcccggaaatatggacatctacaccctggcctgcctggctccggggggctccggggg
actttgcctctcctggcaccacagcacagccaggcctggatcccacggctctgtcctgagccggc
tgagtatgtggccctgcagagtgtgtggccttgttgggcaccccatccaaggggggtgcagcgtgg
ggctctgctctagggatggggaccctgggctgtggcctctgcaccaagaggtgccaccacgagtc
accccaggggtgcaactcggcctggtaggaagcggcctggaggggggatgtctgggaagttggggg
cagcaagccagtggggaggcaggggcgggtctccccagggcccaagctcatgagtgtctgctgcc
ctggctctccccagCCCGGCGCCGTGGTCTCCTCGAGCCTGTGCGAAACctgcaggtgtgagctg
ccgGGTGGCCCCCCATCGGACGCGTTTGTGGTCAGCTGTGAGACCCAGATCTGCAACACACACTG
CCCTGTGgtgagcgctcccaccctgccccgaccctgccctggctcttgggggggcagcggtcgcct
ggattccagccacatgtccatccctcccgcagGGCTTCGAGTACCAGGAGCAGAGCGGGCAGTGC

TGTGGCACCTGTGTGCAGGTCGCCTGTGTCACCAACACCAGCAAGAGCCCCGCCCACCTCTTCTA
Cgtgagtagtggctgccacaaagaggaagggcagggtctgggagcactggggcatgtggggacct
gtcgttgccaggcatgcgtctggcagaggctggggactctctggaagcccacgggctggggtgca
gacagagggacagacggagggagggtcactcaccccggggcctggcctccctccagCCCGGCGAG
ACCTGGTCAGACGCAGGGAACCACTGTGTGACCCACCAGTGTGAGAAGCACCAGGATGGGCTCGT
GGTGGTCACCACGAAGAAGGCGTGCCCCCCGCTCAGCTGTTCTCTGgtgaggtccaggatccccg
ctccagccaagggggcttcaccccctagatgggtttgggggctgtgatcatccctgcagcgcca
gcagacacccccctcctgcttggggctgtccactcctgagcctggccccatgtccccatccctcac
ttctgtgggggccgccgagcgcctcggcactgagggcgcccctctgtcggcacagGACGAGGCCC
GCATGAGCAAGGACGGCTGCTGCCGCTTCTgcccgccgcccccgcccccgtaCCAGAACCgtgag
tacccagcctgctgggcggggcgggctccaccctcagaggtctaggagcagctgggctggtccta
aaccctgtgttcctctccagAGTCGACCTGTGCTGTGTACCATAGGAGCCTGATCATCCAGCAGC
AGGGCTGCAGCTCCTCGGAGCCCGTGCGCCTGGCTTACTGCCGGGGGAACTGTGGGGACAGCTCT
TCCATgtacgtgcctgggcagcaggcagggagacgcgattggctgtggggtgcagtcagggcccc
cagggctctaggtgccagatagacgaggggcaggaccatgaggggccaggcaaagggctctgagg
gtgaggcgggaaaggggtcctgagatggcaagggtggggctggggtaactacatccccagagcct
gtgtcggcatcacgctctcctgtttactgagctccgccaggaacttgccgcagccgccccgagtc
tccctccctcccatcagcacggagccggggtcggccctggtgggactgttggcgcctggggaact
ggcaaaggagagctggttgtcagacactggcagcatgcctccaggagcagggaacacgatgaggc
cgcccagagctcggcacggcgccggcttacggcaggaggctggggtggcgcagcagctggtgctg
agcagcccctgcccacagGTACTCGCTCGAGGGCAACACGGTGGAGCACAGGTGCCAGTGCTGCC
AGGAGCTGCGGACCTCGCTGAGGAATGTGACCCTGCACTGCACCGACGGCTCCAGCCGGGCCTTC
AGCTACACCGAGGTGGAAGAGTGCGGCTGCATGGGCCGGCGGTGCCCTGCGCCGGGCGACACCCA
GCACTCGGAGGAGGCGGAACCCGAGCccagccaggaggcagagagtgGGAGCTGGGAGAGAGGCG
TCCCAGTGTCCCCCATGCAC

# Sequence S8

Refseq genome DNA sequence of human MUC5B: NC_000011.10 Homo sapiens chromosome 11, GRCh38.p14 Primary Assembly (1223124-1261605)

ATGGGTGCCCCGAGCGCGTGCCGGACGCTGGTGTTGGCTCTGGCGGCCATGCTCGTGGTGCCGCA
GGCAGgtaagagcccccactccgcccctctcgatgctgtcttcacggcgggggtctctgcagg
tcgcttgcctgggagcttctcctgcagagtgcacgggcagatcccctacgactccctgagtgtc
ctggatgggaccctacccgtccccaacacagggctctggggccccacgggctcacagtgtcagga
aactcaggggctggcttggatggggtgtccaggagaaggtgggcccctgaccgcagggcaaggcc
cctgggagaccaccgaaagggtcttggtcttgggggtgggacaggagtgggcaatgggggagggg
gtcacagctggggtctctctggagccccatgaggcccaggcatcagagtgagcaggggcaggct
tagcgtggacccctgtccaggaccggctctacccttcacgacctccctggggatcacagctggca
gggcaggtgagggtaccaccctcaagggttgcacagccagccgcaagagccccggcctcaaccca
cgctcgactcccacggcccatctgtgggcatctcatgccgcacgggctgcctggctctcagccga
gcgttttccctcgtctgctgtctcttggccagagccgcagcattaatacttactgtcaatagaga
aagatgcagccccaggggccaccgggagacacccagccgggctggccatgaggctgctgcagccc
ctccctgccccgccctccacccccctcccaagcttggggtctgggctgggcaggtgaggctccctg
gggtctctctccatctgtggaagggaggctgggtggtcagcagggctggaggcaggggcttccc
ccagtggctcccagcctgggcccgggggggagctgcgtctggctgcaaggtttgggggctggtttg
accagaatagccacctccttgcatctgattcttccgggccatgcagccttggctcccctcacctg
agcaggcagggcctagggactctcagcccacccgtcctcctgtcctccacgcacgtccaagttgg
ggagatcaagcccttggcagggactgtgctttagtcaccagatgcacgtcctgtggccggggaag
gcagccctgcacagagcagcttcatgttaggggacacaccccaaagtgatggggtggctggtggt
gggcacttctctggctacaagatggaggcccaggtggtccagcccaaggagggcactgcacggag
cagataaccaagggcagtcagcctgggcaggggagggctgcctgggggggaggggttgcctggg
ttggggaggggctgtctggggcaggggaggagctgcctggggcgggggaggggctgtagggccag
ggaggggctgcctggggctggggaggggctgctggggtggggaggggctgcctgcggcgggagcc
ggggcgtgggagtggctggttgggctggcacacaggggcagggctgtgagctgtgggtcggggtg
gaggactcagggatcggctggctttctgggaaaggcagtcaacctggatctctggaggcggcccc
tgtggtggttcccagatgtcagcaggacctggctggaaaagccaggcagggccaggccagagtgc
gaaccacagggccggcccctcgctgagccctgaccatgctgtgggggctggggcctcacctccca
cctccccacagagagtctcagatcaggatccagggaggagctctggggtcctgtgaagggggcgc
cccaacccaaactgggcagacaatggccgggggtcctcagagtcctgtgggttggagctgcctcc
tcccagcctccatggggttggtgggtgaggccttgcccggaggcggtggtcagcctggggacct
tgggcggccatcccagtatcaacggccacacagcttgcgcggcccagagtcctgcccccagcctg
ccccactcgccctgacttaggatctagttcgaaactggttctgtgtttaggtttctgctaagtca
cgcctggaaggctccaagtgtgtcctcctaacaaagctggtctttgtccttctccaagggatgtg
tgggatggggcgaaatccccccttggggcggccaacgccttttcctgattccattttctcccca
tcccttgagaaggaggcaccatccccgcctgtcagtcggggacagggcaggccgtgctgggggca
gctcagggctccctgctggaagcttccatcccgcaggctttccatagcattgagcaggagcggag
gcatctgcggctgacggttggggtggcctgagcggctggggaggagtcccggccttggccacagt
gtgtcgtgagggtgaacctgcagggcatggagaccgccaccaaggaccccacatgcggctgccgc
accagggatgtggccaggtccgtggttgggttcgtggctggcagccacatctagttcctcactga

ctcccattccctcttcccacagAGACCCAGGGCCCTGTGGAGCCGAGCTGGGAGAATGCAGGGCA
CACCATGGATGGCGgtatgtggccaggttcgggggtgggggttcctgaccaggctggaggggct
ggaatttgggctggggcaggcagacgcctctccaagcagccatgcgtctgacagagaccctccct
gggtcccctgcccaggacaatacccagcacccgaggcggagcttggtgctccaaagaagaggaaa
gtgcagagcagagagacatgcacacagaagcacacgcgtggacaggcacatgcgtgcccacactt
acactggcacacacatgtgtgcacacacaggccaaaacacaagggcagcagtgtttgtggggcag
acagggccaagggtaaaggggctgccttggccccagcccatcagttttgggctccccttcaactc
tggtggctggcgaggagggtgggccccggggagggtgtctctgcttccccttcctggccacgttc
ctggggtgaccagccttcacccacagGTGCCCCGACGTCCTCGCCCACCCGGCGCGTGAGCTTTG
TTCCACCCGTCACTGTCTTCCCCAGCCTGAGCCgtaagcagatgctgcccctgccagccgggaag
gggtgtttgccagtcccaaaggtgggggcccagatctaggggtgcagctgccaccaggtggggc
cgttgggccagacccagagtcctccgtgtgggcggtctcctggtcactggccaccctgggggatg
gggacgggtcaggggtcttggagcaaaacagacgcagtccagggtgagccaggcagggcacagcc
agcagccgaccatgggcttttccattccaaaaaccagggtgcctcggcccaggggaggctacccc
gtgggggctggcatggggatgggcctcatcccgcgctccccacagCCCTGAACCCGGCGCACAA
TGGGCGGGTGTGCAGCACCTGGGGTGACTTCCACTACAAGACCTTCGACGGCGACGTCTTCCGCT
TCCCTGGCCTTTGCAACTACGTGTTCTCTGAGCACTGCCGCGCCGCCTACGAGGACTTCAACGTC
CAGCTACGCCGAGGCCTAGTGGGCTCCAGGCCTGTGGTCACCCGTGTTGTCATCAAGGCCCAGGG
GCTGGTGCTGGAGGCGTCCAACGGCTCCGTCCTCATCAATGGGCAGCGgtgagccggccaccctg
gggaggggcgagggccgggccacacagtgtgacctccccacacggccatgtctgacctgggccag
ggctgggggggggttgggtgggcaggcagccaggagagcggggcccagggagagaccccgctgtc
tgcgcagGGAGGAGCTGCCTTACAGCCGCACTGGCCTCCTGGTGGAGCAGAGCGGGGACTACATC
AAGGTCAGCATCCGGCTGGTGCTGACATTCCTGTGGAACGGAGAGGACAGTGCCCTGgtgaggaa
gccccctcgccccttgccccttcaggcctggccacaaacccccaccgggggtcgagggatgcct
ccctgggcttggggtcacggggcttggggcatgttgccagtgtggggatcagaggtcctgaggct
ggagctgcccctccccactctcagCTGGAGCTGGATCCCAAATACGCCAACCAGACCTGTGGCCT
GTGTGGGGACTTCAACGGCCTCCCGGCCTTCAACGAGTTCTATGCCCACAgtgagtgccacctgg
gtgagggggcggtgaccaattatgtcggccaacgaagagccacagtcccggggaggccgggaggg
ggcggagtggggaccgggcaccaggcagggaggggccacgaggactgtgccctacatggtgggag
gagtgcccctcgggggtgttgggccctaggcaggagtgggagtcctctggcctgggctcaggaag
tgggagcccatatcttgtccccaggagcccctcagagccaccacacccctgctttcttcccggca
gACGCCAGGCTGACCCCGCTCCAGTTTGGGAACCTGCAGAAGTTGGATGGGCCCACGGAGCAGTG
CCCGGACCCGCTGCCCTTGCCGGCCGGCAACTGCACGGACGAGgtgagtcccccgccacccccag
ctcctgggcagggacggcctccaggtccaggggggagctgggccgaggtctgaggaatgttcccag
ctggtggagagatggtgccattggagggaggccgggcagccaccctctgtgtgctcagttccacg
gtacacactgtccgagtgtggtgacgtgcgtgttcatcaggccacgcgtgtgcccatctgtgtga
gcaaacacaggcccatgctgcacaggctgggctgagggtgggcactcgggaagcccggagccagc
ccttcccaccagcaggtggactcagaaggggcctggaggctccaggatccccaaaccagcaggat
ctctgagccttaagttgtgctgtgaatgacagcatgagccccccctgtgagctgggccccgcagcc
ggcagccctgggcctggggacggaggacactcagcactggactgccctgaacctgccgggctgcc
cagagaggcggggcctccacctcccctccttggctccgcctcctggggtgggggtctgcaccttt
cttgggcgcttactccacgggcaggcacatccggagtaggggatccccggtcacgggtcactccc
caagggccaagcagagctctgcatggccacagtgggtggaaggggtggggctgggtacaaggaac
cccgacagggagagggcttcccggcctggcctgccatgggtcctattccagcaccgtggcagccc

ccatggatggcaggggtgcccagcctggcccactgtgctccccagGAGGCATCTGCCACCGCAC
CCTGCTGGGGCCGGCCTTTGCGGAGTGCCACGCACTGGTGGACAGCACTGCGTACCTGGCCGCCT
GCGCCCAGGACCTGTGCCGCTGCCCCACCTGCCCGTGTGCCACCTTTGTGGAATACTCACGCCAG
TGCGCCCACGCGGGGGGCCAGCCGCGGAACTGGAGGTGCCCTGAGCTCTGCCgtgagtgctccca
gggccttcgccagggattgtgccagagagaaggggcaggggggagcgccttgggggccactggggg
tggggaggcctgggggacaggggtggagggcagaggacccaccccaggcatagtgggcagaggcc
accccaggaccccaggaggggggtggggcctccgggggctgcaggagaaggagaggctgtggagag
gctgtgcagcaggtggcgggggctggggctggagggtggagctgcccacgatgaggggcgtcagg
gccaccctggggcctagctctggcttctgtggacttgatggcatgtggaaggccgtggaaggcgg
ctggggctgaccacacgggcagtacagggcccttcccctggcccagccccacctccttttgcgca
gCCCGGACCTGCCCCCTCAACATGCAGCACCAGGAGTGTGGCTCACCCTGCACGGACACCTGCTC
CAACCCCCAGCGCGCGCAGCTCTGCGAGGACCACTGTGTGGACGGCTGCTTCTGCCCCCCAGgca
ggtcttgtgtgccctgaacccctcaggggggctttcaggtccctgctcccaaccccgcccccagcc
tcatcaggcgtggaagcagagcccctcatgccagaaggtcccaccagagggcccagggtgggaag
ggcactggctgggagggtgccggaagacctgccgatgcgtggagggaggtagagcagtgccatga
gccagctgggcatggtggggaaactgaggcccagaggtgcttggtgttcatccaagcgagtgcag
ctcagggcgggggcggtgtcctggagcaggaattcctccccaagggaggcagcttgtccccaagg
ccggtgtcttctgaccttggtgtcccccgtgcatgggccggccctgcctcacgccgcgccccaca
gGCACGGTGCTGGATGACATCACGCACTCTGGCTGCCTGCCCCTCGGGCAGTGCCCCTGCACCCA
CGGCGGCCGCACCTACAGCCCGGGCACCTCCTTCAACACCACCTGCAGCTCCTGgtacttatgag
cccaccagcctccgcctggggtggggtgtggagctcctggtatttatgaacccgccagcctctgc
ctggggtgggggtgtggagctcctggtgtgcacccaccagcctccgcctgcggtgggggtgtgga
gggtggggcccacctcctcccgacatgccggttctgctcacggcctccctccccagCACCTGCTC
CGGGGGGCTATGGCAGTGCCAGGACCTGCCGTGCCCTGGCACCTGCTCTGTGCAGGGCGGGGCCC
ACATCTCCACCTATGATGAGAAACTCTACGACCTGCATGGTGACTGCAGCTACGTTCTGTCCAAG
gtctgggcttggggccgggtcttcagacacccagaccctcctgggaccctcatgccacttccacc
caggggaggcccccacgatggtcatagaggggtggatgtccctgctgagggggggagccctgggtc
cccatgatggtcatagagggatggctctccctgctgagcggcatggggccaaggagcccccaggc
cctgagacaagctgctgggaggtgaccagaggtgccaaggaccacctccccacagagccacatcc
cccacatgggcatccccagcacacttctgggggggcaccccacatcatcgagccaggcccaatgca
cgcgtgggtccttctccccagAAATGTGCCGACAGCAGCTTCACCGTGCTGGCTGAGCTGCGGAA
GTGCGGCCTGACGGACAACGAGAACTGCCTGAAAGCGGTGACGCTCAGCCTGGACGGCGGGGACA
CGgtgaggacctggctggggccctgggctgggacaggaagaggcatgcgaaggtgtgtggggagc
aagcacggtcaggtccccctccagccccgaggccaggtccccccctccagcccccaggccaggtcc
ccctccagcccccaggtcaggtccccccctccagccctgaggtcaggtcctcccgggggggcaatt
gcagagcccaccgcaggtccaggtctgagcttctctgtgggctctgtccccagtgggggcccctg
ggcaggccacccccctcatttgagagtcgggaatgggttcctccccagagctgacctcccgcccgc
ctccttccgcagGCCATCCGGGTCCAAGCGGACGGCGGCGTGTTCCTCAACTCCATCTACACGCA
GCTGCCCCTGTCGGCAGgtatgtggctctcccaggacggccgggctgggtggcgcctgcttgcag
gggcagctcccacagcctgggcagcgtccgctccatccctgctagttctccgtggcctcgggcag
ctccaggagctccctgtgctcggtttctcgtctgcagagtggggatgccaggctcccaccccggc
agcggcagggaccccacatccagctcgctcagccccactctctcagggagcccggtctccacctg
agcccacttggcggccacaggcatgggacagggagcctgagggctcctggccactcctgggtctc
actcccgggtctcagtggggtggcccggcccactggatgccctgcccctccaatctagccagatc

tgtccctgcacccctgaccggcctctcccccacactcccggcagCCAACATCACCCTGTTCACAC
CCTCGAGCTTCTTCATCGTGGTGCAGACAGGCCTGGGGCTGCAGCTGCTGGTGCAGCTGGTGCCA
CTCATGCAGGTGTTTGTCAGGCTGGACCCCGCCCACCAGGGCCAGATGTGCGgtgaggctgggca
ggggccttcggggacagggccattggggacggggcctggactagcgccaggctgcagggaggggc
aggcagaggcgggcaggggaccggggagggggctgcccccagggcatggcggagatcctggtgcc
agcgcaggacaccagcattggaccagcggccccggaagcagccagctgggaggatggagcgggca
gccctgccctggctcaggccgactttgcacaggggctggctttgcacaggggccgactgcacagg
ggcgccccccgccagggcttatctgcagagggttctgggagcagaatcctgggacagggctccca
gccgttccaccctgtgtggtgcctggagggatggcaggggccaggagccaggtgggcccaacagt
ggccgctgacatcccccaaccctggcccccagGCCTGTGTGGGAACTTCAACCAGAACCAGGCTG
ACGACTTCACGGCCCTCAGCGGGGTGGTGGAGGCCACGGGCGCAGCCTTCGCCAACACCTGGAAG
GCCCAGGCTGCCTGTGCCAATGCCAGGAACAGCTTTGAGGACCCCTGCTCCCTCAGTGTGGAGAA
TGgtactcctcgcccccacccccacagtcaccccaggctcaagtcccacccagcaccttcctgtc
ccctgggccacggggacccctgggtgggattggggaccccatggaggcaggtgggaggcatcagg
aggaggtgcttggggccaggcggccagaacccccaaggcgcagcaggtgagccgcaaattccaa
ctcactgttccccgggctgaggggggtcgcaggcctgcgtgtcaggggtgtgggcttcggggcagg
gcgtggagatgaggtcaggtcttccccacagAGAACTACGCCCGGCACTGGTGCTCGCGCCTGAC
CGATCCCAACAGTGCCTTCTCGCGCTGCCACTCCATCATCAACCCCAAGCCCTTCCACTCGgtga
gaggctgaggccagacccccacgcctgggcaggatgggtgggggagccctggcaggctggggtcc
ctgacgccccgatgcctcccacctccgcagAACTGCATGTTTGACACCTGCAACTGTGAGCGGAG
CGAGGACTGCCTGTGCGCCGCGCTGTCCTCCTATGTGCACGCCTGTGCCGCCAAGGGCGTACAGC
TCAGCGACTGGAGGGACGGCGTCTGCAgtgagtgcccacgctgggggtgggatgtgtccacaccg
cgtgggggtgcgggggaccctggccggcagcagccgtcactcacacggttctcagcccagagctt
tgcacttcctcatcccagcctcgcaagaacctcatgcccttgcgatccccacgtcacagacgggg
atgctgagttgaagatgggggctggccaggctgctcggccgctgacctgtccccccctggccccac
cgaccacagCCAAGTACATGCAGAACTGCCCCAAGTCCCAGCGCTACGCCTACGTGGTGGATGCC
TGCCAGCCCACTTGCCGCGGCCTGAGTGAGGCCGACGTCACCTGCAGCGTTTCCTTCGTGCCTGT
GGACGGCTGCACCTGCCCCGCGGGCACCTTCCTCAATGACGCGGGCGCCTGTGTGCCCGCCCAGG
AGTGCCCCTGCTACGCTCACGGCACCGTGCTGGCTCCTGGAGAGGTGGTGCACGACGAGGGCGCC
GTGTGgtaagggtctgggggggaaagcaggcccccccaggtgctcctcagagccacttcccgccctc
cccgaaggcttctgtgcctccccccgagggttctgagacatgaggggccaggctggggagagtgg
ggcagggtggacccagcacattctgaagagaaaattcccagctgggaaagaggccaggagaggag
gtggccctgggaggacacctgctggctgttctcagctgggtccacatggcagcccctgccaggaa
aggtgggtggcccccactcccaccctgggctcaaaggccgctcctaaccccagggtcctggctgc
tttgctgccccccctgtgtgtatttacccatgtgcctccaggggatttgggggctcccagcaaaca
cagcagcaggcaccgtctggccttacaaggaggtggccaggctggggaggcccagcgttcggcgg
gggctcggaagcccggggggtggggtctgcggggtgagggccgcagatccaggctgtgccgtctgt
ctcttgtagTTCATGTACGGGTGGGAAGCTAAGCTGCCTGGGAGCCTCTCTGCAGAAAAGCACAG
gtaagtgccacccctgccctgccctgccctgccccgccccgcatcaccccgcctggcctggcccc
aacacgccccaccctgccccaccccacctgaaccctgccgggccaggtcagtcctcacctgggct
ctgccacaggcacccatgccctgacacgccagggacggaggggccagtgggtctctgccccgcag
tgtggccggggtgtcctggggttgggggctgcaggtgtcatggaagctttggctcgggggctgtt
aacttgatcagcaggacaggctcagggctgcctggggtcagttgagggccgtggctgcccttccc
caggacccctcccaccaagctctgtccccagGGTGTGCAGCCCCCATGGTGTACCTGGACTGCAG

CAACAGCTCGGCGGGCACCCCTGGGGCCGAGTGCCTCCGGAGCTGCCACACGCTGGACGTGGGCT
GTgtgagttccatgcttcagggaggggtgggcagggaaggggtcccagctttcccagctcccgag
cccagggatctggtggtcctggagacacttacccacctggaagctccgccctggcccatgcgttg
ccctgggtgctgctgggtgcgcctgtcccagagggtgagtgacatctgcccaccctggtgtccag
ccctgaccggtacctgcctgggccccacagTTCAGCACACACTGCGTGTCCGGCTGTGTCTGTCC
CCCGGGGCTGGTGTCGGATGGGAGTGGGGGCTGCATTGCCGAGGAGGACTGCCCCTGTGTGCACA
ACGAGGCCACCTACAAGCCTGGAGAGACCATCAGGGTCGACTGCAACACCTGgtgggtcgtgagt
ctctcggaggcagcaggtggggagggcgggggcggggagggcagcgggtggggaggcagcgggca
gggagggcagggggcggggagggcagggggccagctggccagggtgaggtggggccgtggcagga
gagagagttgctaggaaagccatgggccgtcctgtgcgtcctctggaaggtggcccaggggccgt
ggtgctaccaggagcctggtggggctgcgtgccctgcattcacagtgggggacaccacttcttcc
acggaggaggggtcaggctgggcctggggaggctgaggccccgtgctgacctgcacaggcctggg
tgccgggtctcaggaaggccgggagagcaggcccctgtgagcaggcaccattgtggccccttgca
gCACCTGCAGGAACCGGAGGTGGGAGTGCAGCCACCGGCTCTGCCTGGGCACCTGCGTGGCCTAC
GGGGATGGCCACTTCATCACCTTTGATGGCGATCGCTACAGCTTTGAAGGCAGCTGCGAGTACAT
CTTGGCCCAGgtacgccgcccctcgcccactcctgcaggccgggcacactccagcccgcggcca
gcagcttgtctctttctggcccagGACTACTGTGGGGACAACACCACCCACGGGACCTTCCGCAT
CGTCACCGAGAACATCCCCTGTGGGACCACCGGCACCACCTGCTCCAAGGCCATCAAGCTCTTCG
TGGAGgtgagaacggccccagctgtgagcacccccgaccctgcagccaacgagccggcccccagg
gaagcttcgtgaggctttagctgcacccacaggttctcagcagtgtcctggccccgggctgctgt
tccaagcagccacaaaccaggggggcttagacaacagaaatgcattctcagtcctggagccggaag
tcagagatccaggcgggcagggccacactccctgtcgagggtctggggaggtccttcctgcctct
cccagcttcacaggcggcaggcgtccctgggctgtggctgcctgtggcctcccgctgtgtctgcg
tctgtcttctctctgtttttctcttctgtctcttgtaaggacactggtcattggatttagggccc
ccccccgcccccacgtagtccaggatgatctcatttcaagatgcttcacttaatcccgtctgcag
agatgctttctcccagtgagggcccgggctgaggttctgggagttcgcatgtggacaggcatttt
caggagccacgattcaccctgccacacctagagacacccactccagcaaagggggccagagctc
ccaggggataaagcagcgccgctggccgggatgctccctgcagatggcgggaggggctgaggacc
gcagcgggtcaggggaggctggtgtgagggcgtgggggctgcagggctggatggggagcagggtg
gggtggagtgggcctactgcagcctctgctgctcccgtgcagccccaaggttcccaggcagcccc
tgttcccagcacttcctggccagcctcttgccaaaccttcgctgagggtctcacggacccagctc
accccctaacgccagccgcttgtgctaagagcccgtgcgcacctgcagagcactgggtggggcatc
cctgggtctcaggcccctccctgggggccacagggtcggcttccggcagcgtctgcctcccctgc
agAGCTACGAGCTGATCCTCCAAGAGGGGACCTTTAAGGCGGTGGCGAGAGGGCCGGGTGGGGAC
CCACCCTACAAGATACGCTACATGGGGATCTTCCTGGTCATCGAGACCCACGGGATGGCCGTGTC
CTGGGACCGGAAGACCAGCGTGTTCATCCGACTGCACCAGGACTACAAGgtgagctcgggccgtg
cactcctaggccctgcaggaccctctcacagtgacagaaaccctggtgccaggtggggtctgtgg
gactcgctgacccgtgggtgcgtgagcctggctggtgagggccctgcctgtggcctccacagtgg
gcagaggattttgcagggaagcaggtgccacccagcggcccacccagggacccactgcacacctg
tctcctacaagttcaccaggcactgcctggggaaccggctgccctccctccatcccccgagggct
ctggagcccagggtgggctctgtgctgcctcccacgggtgcctgtggccccagctccaggcccc
actctctcgctgcctctgcagGGCAGGGTCTGCGGCCTGTGCGGGAACTTCGACGACAATGCCAT
CAATGACTTTGCCACGCGTAGCCGGTCCGTGGTGGGGGACGCACTGGAGTTTGGGAACAGCTGGA
AGCTCTCCCCCTCCTGCCCGGACGCCCTGGCACCCAAGGACCCCTGCACGGCCAACCCCTTCCGC

AAGTCCTGGGCCCAGAAGCAGTGCAGCATCCTCCACGGCCCCACCTTCGCCGCCTGCCGCTCCCA
Ggtggggctctggtcttggcaggcagggtctggtggggatggcagttgcttccttcccgccgaga
actgggtcttctgggcagacagcagcgctccaaggagggtctgaccatgtcccacggcacacagt
cctggatgtcaggtcccaagtccggatctcccgtcagccccacacctgtgcctcttgcccctggc
acgaagccatcttggctgtttcccggccactcctttgaccacagcctcagtcacacccagaggct
cacagggaggggcagccctctatgtggcccctagccaccctcctctatgatccccagacctgccc
agtcctcagcacaaactggaatgccagcctggctccccgctcagccagggaggaatcagagatct
gccctaagcagagacttccaaaaagcagtttcctgactgggcgcggtggctcatgtctgtaatcc
cagcactttgggacgctgaggcaggtggatcacctgaggtcaggagtttgagaccagcctggtca
acatggcgaaaccccgtctctacaaaaaatacaaaaatagccgggtgtggtggtgtgtgcctgta
atcccagctactcgggaggctgaggcaggagaatcacttgaacctgggaagaggaggttgcagtg
agccaagatcgtgccactgcactccagcctaagcaaaaagagtgagactctgtctcaaaacaaaa
caacaaaaaaccaaaaagcagtttcgtgtcatcttaaggaagacttgagtgcccacttaggcaca
cagcatggtggctcaggagctgagatgaggggctggcgtaggggcagcagtgggcatactcgctc
gtgggaggccctgaagcactctcatgtcggccgccgcttgccctcttgagaaggcagctggtgac
cccttggaaggtcctgtggcctgacaaagctgagcccaggttcagatggggcctgggagggtgt
gggctgcctggaggaagcaggcagcttcccatggtcaggacgcattcacagctcagctccccgcg
tggctggtctggaaaggaagtgaccactccttccttagtgcacattcactgggtgcctggaatag
cctggcatgttctgggctcaccccagtgatcaggggacgaggctgaccctcacagagcttccaga
ggaggcagaaaggcggtgggtgctgggtggtcggatgctaggatgtggagggccctggccggggg
ttggttccgctggagggaaggcccccaggtggaaaggaggccagtacgactgcagcggagggagg
tgggggcgagggcagagggtaagcaggggtgctatgctccacatgggtttgaaacctgtgggcca
catgaccagatccacgtgatagaaagatccaaagagcacatgtgaaggcaggcagatgggcaggt
gcataggtgggcaggtgcataggtgggcagatggacaggtgggcagatgggcaggtgggcaggga
tataggtggacgagggcacaggtgggctggagaagtgctggggcagctcccatttggggcacgct
ctgaggtattccaggccccaggagctcagagagctgccatggggggtgttgaaatacagatggtt
ccagcaactggccctgggccagccacccccctggccgggggggccattgtcccggctgagctgcac
cttggcctcacccgcagGTTGACTCCACCAAGTACTACGAGGCCTGCGTGAACGACGCGTGTGCC
TGCGACTCGGGTGGCGACTGCGAGTGTTTCTGCACGGCTGTGGCTGCCTACGCCCAGGCCTGCCA
CGACGCGGGCCTGTGTGTGTCCTGGCGGACTCCGGACACCTGCCgtgagtcgggctctgtccgtg
gtgctgaagggtggagctgctggggcaggggaggaggtgtggcagcctccgaaggtgcattgacc
tgggcctgagccgcacacagacatccaacacgcatgtgcctccatgtgagtgcacaagtttctat
gcacagaggaagacctgtgcaaaaccaccagacaggttgccccagcatgagacagctcctagggg
acaagagttccaagggcagggctggggagtggaggggaaggtgaggcaccacccggccgaggccc
tgcatgtctgggacaagcccgggtctggctctggggacaccggcccccacgcccggggtaggggc
tgccctgcacaacaggggtgagggctggtgggcgcctccttagcctctgccctctgtgccccagC
CTTGTTCTGTGACTTCTACAACCCACATGGGGGCTGTGAGTGGCACTACCAGCCCTGCGGGGCAC
CCTGCCTAAAAACCTGCCGGAACCCCAGTGGGCACTGCCTGGTGGACCTGCCTGGCCTGGAAGgt
gaggggcagcctttcttggatggagcctcctctccttgggttcccgagtgtacgtgggggggcgg
ggatccccagggacgcggtgtaggctcccgtaaactgcacaatgcaagccttgagggcaggcccc
tgctggctggtgggggggcggctactccctgcagcatggagcccctggctggagagactaaaggc
cctggtgagtcttctgctcaccctgccggccctagGCTGCTACCCGAAGTGCCCACCCAGCCAGC
CCTTCTTCAATGAGGACCAGATGAAGTGCGTGGCCCAGTGTGGCTGCTACGACAAGGACGGAAAC
TACTATGACGTCGGTGCAAGGGTCCCCACAGCGGAGAACTGCCAGAGCTGgtgagggggtgggaa

gcgggtggcgctgggggagcagggctggggagcaggccctgcaggctgcccccaggccctcagc
tcgcctctcccccacccctagTAACTGCACACCCAGTGGCATCCAGTGCGCTCACAGCCTTGAGG
gtaaggaagggccgggggttagtgggccggtgaaggctggggccaggggctcggaggccctggg
tgactctgccggctccatccccagCCTGCACCTGCACCTATGAGGACAGGACCTACAGCTACCAG
GACGTCATCTACAACACCACCGATGGGCTTGGCGCCTGCTTGATCGCCATCTGCGGAAGCAACGG
CACCATCATCAGGAAGGCTGTGGCATGTCCTGGAACTCCAGCCACAACGCCATTCACCTTCACCA
CCGCCTGGGTCCCCCACTCCACGACAAgtaagccctgcctggctctcctgaggcccagtaccgtc
tgggtgacaaggaggacccccctgggctcttagtgcaggtgccctgtatggtagcgacagtcccaa
tccactgaccttccgggctctgtctaggggtgcacggcccctcaacaccctgcgtgtctccaggg
gctccccacgaagcctcagcacaatgattgatgggataccccaaggagacaataaagctttcctg
gactccgtcccatccctcagcacggcctatcccagccagccagctccctcaaggccaggctgcca
ggccccagtccctcatgcagaaacggctctaaccaaggctgaggcaggcactggggtccccagta
tcccacaggggcagggccagccctggggaaagggtcctctggggcccctccaccttgtgaggcca
ggactggaggatgctgagccaggacccctttcccatgccccttgcagGCCCGGCCCTCCCGGT
CTCCACCGTGTGTGTCCGCGAGGTCTGCCGCTGGTCCAGCTGGTACAATGGGCACCG
CCCAGAGCCCGGCCTGGGAGGCGGAGACTTTGAGACGTTTGAAAACCTGAGGCAGAG
AGGGTACCAGGTATGCCCTGTGCTGGCTGACATCGAGTGCCGGGCGGCGCAGCTTCC
CGACATGCCGCTGGAGGAGCTGGGCCAGCAGGTGGACTGTGACCGCATGCGGGGGCT
GATGTGCGCCAACAGCCAACAGAGTCCCCCGCTCTGTCACGACTACGAGCTGCGGGT
TCTCTGCTGCGAATACGTGCCCTGTGGCCCCTCCCCGGCCCCAGGCACCAGCCCTCA
GCCCTCCCTCAGTGCCAGCACGGAGCCTGCTGTGCCTACCCCAACCCAGACCACAGC
AACCGAAAAGACCACCCTATGGGTGACCCCGAGCATCCGGTCGACGGCGGCCCTCAC
CTCGCAGACTGGGTCCAGCTCAGGCCCCGTGACGGTCACCCCCTCGGCCCCAGGTAC
CACCACCTGCCAGCCCCGGTGTCAGTGGACAGAGTGGTTTGATGAGGACTACCCCAA
GTCTGAACAACTTGGAGGGGACGTTGAGTCCTACGATAAGATCAGGGCCGCTGGAGG
GCACTTATGCCAGCAGCCTAAGGACATAGAGTGCCAGGCCGAGAGCTTCCCCAACTG
GACCCTGGCACAGGTGGGGCAGAAGGTGCACTGTGACGTCCACTTCGGCCTGGTGTG
CAGGAACTGGGAGCAGGAGGGCGTCTTCAAGATGTGCTACAACTACAGGATCCGGGT
CCTCTGCTGCAGTGACGACCACTGCAGGGGACGTGCCACAACCCCGCCACCGACCAC
AGAGCTGGAGACGGCCACCACCACCACCACCCAGGCCCTGTTCTCAACGCCGCAGCC
TACGAGTAGCCCGGGGCTGACCAGGGCTCCCCCGGCCAGCACCACAGCAGTCCCCAC
CCTCTCAGAAGGACTGACATCCCCCAGATACACAAGCACCCTTGGTACAGCCACCAC
GGGAGGCCCCACGACGCCTGCAGGCTCCACAGAACCCACTGTCCCAGGGGTGGCCAC
ATCCACCCTTCCAACACGCTCAGCCCTTCCAGGGACGACGGGGAGCTTGGGCACATG
GCGCCCCTCACAGCCACCCACGCTGGCCCCAACAACAATGGCAACCTCCAGAGCTCG
CCCGACAGGCACAGCCAGCACCGCTTCCAAAGAGCCGCTGACCACGAGCCTGGCGCC
AACACTCACGAGCGAGCTGTCCACCTCTCAGGCCGAGACCAGCACGCCCAGGACAGA
GACGACAATGAGCCCCTTGACTAACACCACCACCAGCCAGGGCACGACCCGCTGTCA
ACCGAAGTGTGAGTGGACAGAGTGGTTTGACGTGGACTTCCCAACCTCAGGGGTTGC
AGGCGGGGACATGGAAACTTTTGAAAACATCAGGGCTGCTGGGGGCAAGATGTGCTG
GGCACCAAAGAGCATAGAGTGCCGGGCGGAGAACTACCCCGAGGTAAGCATCGACCA
GGTCGGGCAGGTGCTGACCTGCAGCCTGGAGACGGGGCTGACCTGCAAGAACGAAGA
CCAGACAGGCAGGTTCAACATGTGCTTCAACTACAACGTGCGTGTGCTTTGCTGTGA
CGACTACAGCCACTGCCCCAGTACCCCAGCCACCAGCTCCACGGCCACGCCCTCCTC

AACTCCGGGGACGACCTGGATCCTCACAAAGCCGACCACAACAGCCACTACGACTGC
GTCCACTGGATCCACGGCCACCCCGACCTCCACCCTGAGAACAGCTCCCCCTCCCAA
AGTGCTGACCACCACGGCCACCACACCCACAGTCACCAGCTCCAAAGCCACTCCCTC
CTCCAGTCCAGGGACTGCAACCGCCCTTCCAGCACTGAGAAGCACAGCCACCACACC
CACAGCTACCAGCGTTACACCCATCCCCTCTTCCTCCCTGGGCACCACCTGGACCCG
CCTATCACAGACCACCACACCCACGGCCACCATGTCCACAGCCACACCCTCCTCCAC
TCCAGAGACTGCCCACACCTCCACAGTGCTTACCGCCACGGCCACCACAACTGGGGC
CACCGGCTCTGTGGCCACCCCCTCCTCCACCCCAGGAACAGCTCACACTACCAAAGT
GCCAACTACCACAACCACGGGCTTCACAGCCACCCCCTCCTCCAGCCCAGGGACGGC
ACTCACGCCTCCAGTGTGGATCAGCACAACCACCACACCCACAACCAGAGGCTCCAC
GGTGACCCCCTCCTCCATCCCGGGGACCACCCACACCGCCACAGTGCTGACCACCAC
CACCACAACTGTGGCCACTGGTTCTATGGCAACACCCTCCTCTAGCACACAGACCAG
TGGTACTCCCCCATCACTGACCACCACGGCCACTACGATCACGGCCACCGGCTCCAC
CACCAACCCCTCCTCAACTCCTGGGACAACTCCCATCCCCCCAGTGCTGACCACCAC
CGCCACCACACCTGCAGCCACCAGCAACACAGTGACTCCCTCCTCTGCCCTAGGGAC
CACCCACACACCCCCAGTGCCGAACACCATGGCCACCACACACGGGCGATCCCTGCC
CCCCAGCAGTCCCCACACGGTGCGCACAGCCTGGACTTCGGCCACCTCGGGCATCTT
GGGCACCACCCACATCACAGAGCCTTCCACGGTGACTTCCCACACCCTAGCAGCAAC
CACCGGTACCACCCAGCACTCGACTCCAGCCCTTTCCAGCCCTCACCCTAGCAGCAG
AACCACCGAGTCACCCCCTTCTCCAGGGACGACCACCCCGGGCCACACCACGGCCAC
CTCCAGGACCACAGCCACGGCCACACCCAGCAAGACCCGCACCTCGACCCTGCTGCC
CAGCAGCCCCACATCGGCCCCCATAACCACGGTGGTGACCATGGGCTGTGAGCCCCA
GTGTGCCTGGTCAGAGTGGCTGGACTACAGCTACCCCATGCCGGGGCCCTCTGGCGG
GGACTTTGACACCTACTCCAACATCCGTGCGGCCGGAGGGGCCGTCTGTGAGCAGCC
CCTGGGCCTCGAGTGCCGTGCCCAGGCCCAGCCTGGTGTCCCCCTGCGGGAGTTGGG
CCAGGTCGTGGAATGCAGCCTGGACTTTGGCCTGGTCTGCAGGAACCGTGAGCAGGT
GGGGAAGTTCAAGATGTGCTTCAACTATGAAATCCGTGTGTTCTGCTGCAACTACGG
CCACTGCCCCAGCACCCCGGCCACCAGCTCTACGGCCATGCCCTCCTCCACTCCGGG
GACGACCTGGATCCTCACAGAGCTGACCACAACAGCCACTACGACTGAGTCCACTGG
ATCCACGGCCACCCCGTCCTCCACCCCAGGGACCACCTGGATCCTCACAGAGCCGAG
CACTACAGCCACCGTGACGGTGCCCACCGGATCCACGGCCACCGCCTCCTCCACCCA
GGCAACTGCTGGCACCCCACATGTGAGCACCACGGCCACGACACCCACAGTCACCAG
CTCCAAAGCCACTCCCTTCTCCAGTCCAGGGACTGCAACCGCCCTTCCAGCACTGAG
AAGCACAGCCACCACACCCACAGCTACCAGCTTTACAGCCATCCCCTCCTCCTCCCT
GGGCACCACCTGGACCCGCCTATCACAGACCACCACACCCACGGCCACCATGTCCAC
AGCCACACCCTCCTCCACTCCAGAGACTGTCCACACCTCCACAGTGCTTACCACCAC
GGCCACCACAACCGGGGCCACCGGCTCTGTGGCCACCCCCTCCTCCACCCCAGGAAC
AGCTCACACTACCAAAGTGCTGACTACCACAACCACGGGCTTCACAGCCACCCCCTC
CTCCAGCCCAGGGACGGCACGCACGCTTCCAGTGTGGATCAGCACAACCACCACACC
CACAACCAGAGGTTCCACGGTGACCCCCTCCTCCATCCCGGGGACCACCCACACCCC
CACAGTGCTGACCACCACCACCACAACTGTGGCCACTGGTTCTATGGCAACACCCTC
CTCTAGCACACAGACCAGTGGTACTCCCCCATCACTGACCACCACGGCCACTACGAT
CACGGCCACCGGCTCCACCACCAACCCCTCCTCAACTCCAGGGACAACACCTATCCC
CCCAGTGCTGACCACCACCGCCACCACACCTGCAGCCACCAGCAGCACAGTGACTCC

CTCCTCTGCCCTAGGGACCACCCACACACCCCCAGTGCCGAACACCACGGCCACCAC
ACACGGGCGATCCCTGTCCCCCAGCAGTCCCCACACGGTGCGCACAGCCTGGACTTC
GGCCACCTCAGGCACCTTGGGCACCACCCACATCACAGAGCCTTCCACGGGGACTTC
CCACACCCCAGCAGCAACCACCGGTACCACCCAGCACTCGACTCCAGCCCTGTCCAG
CCCTCACCCTAGCAGCAGGACCACCGAGTCACCCCCTTCTCCAGGGACGACCACCCC
GGGCCACACCAGGGCCACCTCCAGGACCACGGCCACGGCCACACCCAGCAAGACCCG
CACCTCGACCCTGCTGCCCAGCAGCCCCACATCGGCCCCAATAACCACGGTGGTGAC
CATGGGCTGTGAGCCCCAGTGTGCCTGGTCAGAGTGGCTGGACTACAGCTACCCCAT
GCCGGGGCCCTCTGGCGGGGACTTTGACACCTACTCCAACATCCGTGCGGCCGGAGG
GGCCGTCTGTGAGCAGCCCCTGGGCCTCGAGTGCCGTGCCCAGGCCCAGCCTGGTGT
CCCCCTGCGGGAGTTGGGCCAGGTCGTGGAATGCAGCCTGGACTTTGGCCTGGTCTG
CAGGAACCGTGAGCAGGTGGGGAAGTTCAAGATGTGCTTCAACTATGAAATCCGTGT
GTTCTGCTGCAACTACGGCCACTGCCCCAGCACCCCGGCCACCAGCTCTACGGCCAC
GCCCTCCTCCACTCCAGGGACGACCTGGATCCTCACAGAGCAGACCACAGCAGCCAC
TACGACCGCAACCACTGGATCCACGGCCATCCCGTCCTCCACCCCGGGAACAGCTCC
CCCTCCCAAAGTGCTGACCAGCACGGCCACCACACCCACAGCCACCAGTTCCAAAGC
CACTTCCTCCTCCAGTCCAAGGACTGCAACCACCCTTCCAGTGCTGACAAGCACAGC
CACCAAATCCACAGCTACCAGCTTTACACCCATCCCCTCCTTCACCCTTGGGACCAC
CGGGACCCTCCCAGAACAGACCACCACACCCATGGCCACCATGTCCACAATCCACCC
CTCCTCCACTCCGGAGACCACCCACACCTCCACAGTGCTGACCACGAAGGCCACCAC
GACAAGGGCCACCAGTTCCATGTCCACCCCCTCCTCCACTCCGGGGACGACCTGGAT
CCTCACAGAGCTGACCACAGCAGCCACTACAACTGCAGCCACTGGCCCCACGGCCAC
CCCGTCCTCCACCCCAGGGACCACCTGGATCCTCACAGAGCCCAGCACTACAGCCAC
CGTGACGGTGCCCACCGGATCCACGGCCACCGCCTCCTCCACCCGGGCAACTGCTGG
CACCCTCAAAGTGCTGACCAGCACGGCCACCACACCCACAGTCATCAGCTCCAGAGC
CACTCCCTCCTCCAGTCCAGGGACTGCAACCGCCCTTCCAGCACTGAGAAGCACAGC
CACCACACCCACAGCTACCAGCGTTACAGCCATCCCCTCTTCCTCCCTGGGCACCGC
CTGGACCCGCCTATCACAGACCACCACACCCACGGCCACCATGTCCACAGCCACACC
CTCCTCTACTCCAGAGACTGTCCACACCTCCACAGTGCTTACCACCACGACCACCAC
AACCAGGGCCACCGGCTCTGTGGCCACCCCCTCCTCCACCCCAGGAACAGCTCACAC
TACCAAAGTGCCGACTACCACAACCACGGGCTTCACAGCCACCCCCTCCTCCAGCCC
AGGGACGGCACTCACGCCTCCAGTGTGGATCAGCACAACCACCACACCCACAACCAG
AGGCTCCACGGTGACCCCCTCCTCCATCCCGGGGACCACCCACACCGCCACAGTGCT
GACCACCACCACCACAACTGTGGCCACTGGTTCTATGGCAACACCCTCCTCTAGCAC
ACAGACCAGTGGTACTCCCCCATCACTGACCACCACGGCCACTACGATCACAGCCAC
CGGCTCCACCACCAACCCCTCCTCAACTCCAGGGACAACTCCCATCCCCCCAGTGCT
GACCACCACCGCCACCACACCTGCAGCCACCAGCAGCACAGTGACTCCCTCCTCTGC
CCTAGGGACCACCCACACACCCCCAGTGCCGAACACCACGGCCACCACACACGGGCG
GTCCCTGCCCCCCAGCAGTCCCCACACGGTGCGCACAGCCTGGACTTCGGCCACCTC
GGGCATCTTGGGCACCACCCACATCACAGAGCCTTCCACGGTGACTTCCCACACCCC
AGCAGCAACCACCAGTACCACCCAGCACTCGACTCCAGCCCTGTCCAGCCCTCACCC
TAGCAGCAGGACCACCGAGTCACCCCCTTCTCCAGGGACGACCACCCCGGGCCACAC
CAGGGGCACCTCCAGGACCACAGCCACAGCCACACCCAGCAAGACCCGCACCTCGAC
CCTGCTGCCCAGCAGCCCCACATCGGCCCCCATAACCACGGTGGTGACCACGGGCTG

TGAGCCCCAGTGTGCCTGGTCAGAGTGGCTGGACTACAGCTACCCCATGCCGGGGCC
CTCTGGCGGGGACTTTGACACCTACTCCAACATCCGTGCGGCCGGAGGGGCAGTCTG
TGAGCAGCCCCTGGGCCTCGAGTGCCGTGCCCAGGCCCAGCCTGGTGTCCCCCTGCG
GGAGTTGGGCCAGGTCGTGGAATGCAGCCTGGACTTTGGCCTGGTCTGCAGGAACCG
TGAGCAGGTGGGGAAGTTCAAGATGTGCTTCAACTATGAAATCCGTGTGTTCTGCTG
CAACTACGGCCACTGCCCCAGCACCCCGGCCACCAGCTCTACGGCCACGCCCTCCTC
AACTCCGGGGACGACCTGGATCCTCACAAAGCTGACCACAACAGCCACTACGACTGA
GTCCACTGGATCCACGGCCACCCCGTCCTCCACCCCAGGGACCACCTGGATCCTCAC
AGAGCCGAGCACTACAGCCACCGTGACGGTGCCCACCGGATCCACGGCCACCGCCTC
CTCCACCCAGGCAACTGCTGGCACCCCACATGTGAGCACCACGGCCACGACACCCAC
AGTCACCAGCTCCAAAGCCACTCCCTTCTCCAGTCCAGGGACTGCAACCGCCCTTCC
AGCACTGAGAAGCACAGCCACCACACCCACAGCTACCAGCTTTACAGCCATCCCCTC
CTCCTCCCTGGGCACCACCTGGACCCGCCTATCACAGACCACCACACCCACGGCCAC
CATGTCCACAGCCACACCCTCCTCCACTCCAGAGACTGCCCACACCTCCACAGTGCT
TACCACCACGGCCACCACAACCAGGGCCACCGGCTCTGTGGCCACCCCCTCTTCCAC
CCCAGGAACAGCTCACACTACCAAAGTGCCGACTACCACAACCACGGGCTTCACAGT
CACCCCCTCCTCCAGCCCAGGGACGGCACGCACGCCTCCAGTGTGGATCAGCACAAC
CACCACACCCACAACCAGTGGCTCCACGGTGACCCCCTCCTCCGTCCCGGGGACCAC
CCACACCCCCACAGTGCTGACCACCACCACCACAACTGTGGCCACTGGTTCTATGGC
AACACCCTCCTCTAGCACACAGACCAGTGGTACTCCCCCATCACTGATCACCACGGC
CACTACGATCACGGCCACCGGCTCCACCACCAACCCCTCCTCAACTCCAGGGACAAC
ACCTATCCCCCCAGTGCTGACCACCACCGCCACCACACCTGCAGCCACCAGCAGCAC
AGTGACTCCCTCCTCTGCCCTAGGGACCACCCACACACCCCCAGTGCCGAACACCAC
GGCCACCACACACGGGCGATCCCTGTCCCCCAGCAGTCCCCACACGGTGCGCACAGC
CTGGACTTCGGCCACCTCAGGCACCTTGGGCACCACCCACATCACAGAGCCTTCCAC
GGGGACTTCCCACACCCCAGCAGCAACCACCGGTACCACCCAGCACTCGACTCCAGC
CCTGTCCAGCCCTCACCCTAGCAGCAGGACCACCGAGTCACCCCCTTCCCCAGGGAC
GACCACCCCGGGCCACACCACGGCCACCTCCAGGACCACGGCCACGGCCACACCCAG
CAAGACCCGCACCTCGACCCTGCTGCCCAGCAGCCCCACATCGGCCCCCATAACCAC
GGTGGTGACCACGGGCTGTGAGCCCCAGTGTGCCTGGTCAGAGTGGCTGGACTACAG
CTACCCCATGCCGGGGCCCTCTGGCGGGGACTTTGACACCTACTCCAACATCCGTGC
GGCCGGAGGGGCCGTCTGTGAGCAGCCCCTGGGCCTCGAGTGCCGTGCCCAGGCCCA
GCCTGGTGTCCCCCTGGGGGAGTTGGGCCAGGTCGTGGAATGCAGCCTGGACTTTGG
CCTGGTCTGCAGGAACCGTGAGCAGGTGGGGAAGTTCAAGATGTGCTTCAACTATGA
AATCCGTGTGTTCTGCTGCAACTACGGCCACTGCCCCAGCACCCCGGCCACCAGCTC
TACGGCCATGCCCTCCTCCACTCCGGGGACGACCTGGATCCTCACAGAGCTGACCAC
AACAGCCACTACGACTGCATCCACTGGATCCACGGCCACCCCGTCCTCCACCCCGGG
AACAGCTCCCCCTCCCAAAGTGCTGACCAGCCCGGCCACCACACCCACAGCCACCAG
TTCCAAAGCCACTTCCTCCTCCAGTCCAAGGACTGCAACCACCCTTCCAGTGCTGAC
AAGCACAGCCACCAAATCCACAGCTACCAGCGTTACACCCATCCCCTCCTCCACCCT
TGGGACCACCGGGACCCTCCCAGAACAGACCACCACACCCGTGGCCACCATGTCCAC
AATCCACCCCTCCTCCACTCCGGAGACCACCCACACCTCCACAGTGCTGACCACGAA
GGCCACCACGACAAGGGCCACCAGTTCCACGTCCACCCCCTCCTCCACTCCGGGGAC
GACCTGGATCCTCACAGAGCTGACCACAGCAGCCACTACAACTGCAGCCACTGGCCC

CACGGCCACCCCGTCCTCCACCCCAGGGACCACCTGGATCCTCACAGAGCTGACCAC
AACAGCCACTACGACTGCGTCCACTGGATCCACGGCCACCCCGTCCTCCACCCCAGG
GACCACCTGGATCCTCACAGAGCCGAGCACTACAGCCACCGTGACGGTGCCCACCGG
ATCCACGGCCACCGCCTCCTCCACCCAGGCAACTGCTGGCACCCCACATGTGAGCAC
CACGGCCACGACACCCACAGTCACCAGCTCCAAAGCCACTCCCTCCTCCAGTCCAGG
GACTGCAACTGCCCTTCCAGCACTGAGAAGCACAGCCACCACACCCACAGCTACCAG
CTTTACAGCCATCCCCTCCTCCTCCCTGGGCACCACCTGGACCCGCCTATCACAGAC
CACCACACCCACGGCCACCATGTCCACAGCCACACCCTCCTCCACTCCAGAGACTGT
CCACACCTCCACAGTGCTTACCGCCACGGCCACCACAACCGGGGCCACCGGCTCTGT
GGCCACCCCCTCCTCCACCCCAGGAACAGCTCACACTACCAAAGTGCCGACTACCAC
AACCACGGGCTTCACAGCCACCCCCTCCTCCAGCCCAGGGACGGCACTCACGCCTCC
AGTGTGGATCAGCACAACCACCACACCCACAACCACCACACCCACAACCAGTGGCTC
CACGGTGACCCCCTCCTCCATCCCGGGGACCACCCACACCGCCAGAGTGCTGACCAC
CACCACCACAACTGTGGCCACTGGTTCTATGGCAACACCCTCCTCTAGCACACAGAC
CAGTGGTACTCCCCCATCACTGACCACCACGGCCACTACGATCACGGCCACCGGCTC
CACCACCAACCCCTCCTCAACTCCAGGGACAACACCCATCACCCCAGTGCTGACCAG
CACGGCCACCACACCCGCAGCCACCAGCTCCAAAGCCACTTCCTCCTCCAGTCCAAG
GACTGCAACCACCCTTCCAGTGCTGACAAGCACAGCCACAAAATCCACAGCTACCAG
CTTTACACCCATCCCCTCCTCCACCCTGTGGACCACGTGGACCGTCCCAGCACAGAC
CACCACACCCATGTCCACCATGTCCACAATCCACACCTCCTCTACTCCAGAGACCAC
CCACACCTCCACAGTGCTGACCACCACAGCCACCATGACAAGGGCCACCAATTCCAC
GGCCACACCCTCCTCCACTCTGGGGACGACCCGGATCCTCACTGAGCTGACCACAAC
AGCCACTACAACTGCAGCCACTGGATCCACGGCCACCCTGTCCTCCACCCCAGGGAC
CACCTGGATCCTCACAGAGCCGAGCACTATAGCCACCGTGATGGTGCCCACCGGTTC
CACGGCCACCGCCTCCTCCACTCTGGGAACAGCTCACACCCCCAAAGTGGTGACCAC
CATGGCCACTATGCCCACAGCCACTGCCTCCACGGTTCCCAGCTCGTCCACCGTGGG
GACCACCCGCACCCCTGCAGTGCTCCCCAGCAGCCTGCCAACCTTCAGCGTGTCCAC
TGTGTCCTCCTCAGTCCTCACCACCCTGAGACCCACTGGCTTCCCCAGCTCCCACTT
CTCTACTCCCTGCTTCTGCAGGGCATTTGGACAGTTTTTCTCGCCCGgtgagtgcatgt
ggataacactgctgtaccctttccccacatgctatgccaacctgggtctgcctgtcctgggagcc
agtggctttctccctgctggtcatgtttgttccccactggcctcacttggcccctcccggccaca
ctgggtccccactggccacactgggtctcctctggccacaatccgtccgcactggccacaatcag
tccccactggccacacttggtccccactggccacaatgcgtccccctggccacattcggtctcc
cctggacacaatccatccccactggccacactgggtctcctctggccacaatccgtccgcactgg
ccacaatcagtccccactggccacactcggtccccactggccacaatccatccccactggccaca
ctcggtccccactggtcacaatccgtccccagtggccacacttggtccccactggccacactcag
cctctgccctctccactcccttccctggaaccgctgctccctcctgcgctgacctctgcctttgc
tctcccagaactctggcttacccatctctgggagtggcttatctttctatggtgttgttcttcac
agGGGAAGTCATCTACAATAAGACCGACCGAGCCGGCTGCCATTTCTACGCAGTGTGCAATCAGC
ACTGTGACATTGACCGCTTCCAGGGCGCCTGTCCCACCTCCCCACCGCCAGTGTCCTCCGCCCCG
CTGTCCTCGCCCTCCCCTGCCCCTGGCTGTGACAATGCCATCCCTCTCCGGCAGgtgggccccgc
ctgccctccacctcccgtgctgcgtgcacacggtctgggttggctggaggcacagccacagtcca
gctcccgaggcccgtctcagtgaccccgccgccagtatctccagttggaggaggcacacagggcc
taggggccaggctagcatgggggccgccctcctcaggcaggctcttgtggccacccggggctttg

ggccatgaggggtgggatgagccgtggatgggtcccgtgagctggtccaggtgaggacgtgcatg
ttccctgccccagGTGAATGAGACCTGGACCCTGGAGAACTGCACGGTGGCCAGGTGCGTGGGTG
ACAACCGTGTCGTCCTGCTGGACCCAAAGCCTGTGGCCAACGTCACCTGCGTGAACAAGCACCTG
CCCATCAAAGTGTCGGACCCGAGCCAGCCCTGTGACTTCCACTATGAGTGCGAGTgtgagtgcgt
cggtggccgcgggattaccccgggggcaggtggagcagagtgcaccgtcggctaggctggcagaa
tggggcatggtggggcacagtggggtgcagtggggaatggtggggcatggtggggcatggtgggg
tgcagtggggcatggtggggcatggtggggtgtggtggtggtgtttgggagatcgctggcatccc
ttcaggaaaccatcatgcaccatgctgtcttgggcctcagtggtgcacgtcgtgagagctgttag
tatgcaggctccgtgtccacagggctgaaaatgctgacacagcccaagggagaggcagcagaggc
tggtttgctgagcttctctgcacatcagcctgtgtggtttgaaagggaccctgcccaggggcttc
tggggccaggagaagctcaggatggaagcgggagcccagaggagcttttgtctcctgggtcctaa
cagcggcttccatcaccgtgcgggacccaccgctaagaggtcacggcgttctcactcctccccat
gtccttggcccagggctgctgttccaaaccgccacaagctggggagcttatacaacagaaaccca
ctctccgtcctggagctggaagtctgagatccaggcgggcaggggatagactccctgctgagggt
ctggggaggtccttcctgcctctcccagctttggggactccaggtgtcctttggctgtggctgca
tccctccgatctctgcctctgcctctctgtggcctcccctctctgtgtctgtgtctcttctgtct
cccgtaaggacactggtcattggattgagggcccacccagctagtccacgatgatgtcatttcaa
gatgcttcccttaatcccatctgcaaagacactttctcccggcaaagccacatgcggaggttctg
ggatagccatagattttggggaccccattctacacactggacccatttttatagacgaggcagc
tgaggccccaggggcttcagtggctccccaaggcggcagtcacagtggtgacgctggctgccatg
acgcctggggagcgagggcaccaccacggagcctgccagcccgtccatctctgtcccgcagGCAT
CTGCAGCATGTGGGGCGGCTCCCACTATTCCACCTTTGACGGCACCTCTTACACCTTCCGGGGCA
ACTGCACCTATGTCCTCATGAGAGAGATCCATGCACGCTTTGGGAATCTCAGCCTCTACCTGGAC
AACCACTACTGCACGGCCTCTGCCACTGCCGCTGCCGCCCGCTGCCCCCGCGCCCTCAGCATCCA
CTACAAGTCCATGGATATCGTCCTCACTGTCACCATGGTGCATGGGAAGGAGGAGGGCCTGgtga
gtccaggctgcgggtggcacagtgttggcccagtcccaaccgcacctgggcaggccgactgcagg
ccggggtgacccaggtgccgcagcgatggcccagtgcccaagacagctcccaggggggcagggaag
gcctgtggagccgcctaaggccgagcgcaccctgtggcctaaatgcagccagctggagactccag
gcccccagggaagatctgggctttggggtgggggatacacaggggggtctctgcacatggaggca
gagccccaaagagaagccctgctccccgagcccacctggcactgcctcccagctcagggttcccc
tggattcccccagATCCTGTTTGACCAAATTCCGGTGAGCAGCGGTTTCAGCAAGAACGGCGTGC
TTGTGTCTGTGCTGGGGACCACCACCATGCGTGTGGACATTCCTGCCCTGGGCGTGAGCGTCACC
TTCAATGGCCAAGTCTTCCAGGCCCGGCTGCCCTACAGCCTCTTCCACAACAACACCGAGGGCCA
GTGCGgtgagtgggcggcgggtcctgccccggccagggctgctgctgggcctgacaacccagtga
gcataggggaagcctggggaggggaatgagtggggagggggtgggggctgtgaaaggctcccc
agattccagccccgcggtgacgcccccactcccagGCACCTGCACCAACAACCAGAGGGACGACT
GTCTCCAGCGGGACGGAACCACTGCCGCCAGTTGCAAGGACATGGCCAAGACGTGGCTGGTCCCC
GACAGCAGAAAGGATGGCTGCTGGGCCCCGACTGGCACACCCCCCACTGCCAGCCCCGCAGCCCC
GGTGTCTAgcacacccacccccaccccatgcccACCACAGCCGCTCTGTGATCTGATGCTGAGCC
Agtgagtcctcccctcgggggttgcaggccctggggcgcccccgcccgcccgcatgcacgcacgc
acgcagctccctggggctgggggccctccgtcctgatcgctttgccccacagGGTCTTTGCTGAG
TGCCACAACCTTGTGCCCCCGGGCCCATTCTTCAACGCCTGCATCAGCGACCACTGCAGGGGCCG
CCTTGAGGTGCCCTGCCAGAGCCTGGAGGCTTACGCAGAGCTCTGCCGCGCCCGGGGAGTGTGCA
GTGACTGGCGAGGTGCAACCGGTGGCCTGTGCGgtgagtgggggcggccccgggcccccccagacc

cctcggcctctctgagtgtcctgtgcggtgagtgggggcggccccgggcccccagacccctcgg
cctctctgagtgtcctgtgcggtgagtgggggcggccccgggcccccagacccctcggcctctc
tgagtgtcctgtgcggtgagtgggggcggccccgggcccccagacccctcggcctctctgagtg
tcctgtgcggtgagtgggggcggccccgggcccccagacccctcggcctctctgagtgtcctgt
gcggtgagtgggggcggccccgggcccccagacccctcggcctctctgagtgtcctgtgcggtg
agtgggggcggccccgggcccccagacccctcggcctctctgagtgtcctgtgcggtgagtggg
ggcggccccgggcccccagacccctcggcctctctgagtgtcctgtgcggtgagtgggggcggc
cccgggcccccagactcctcggcctctctgagtgtcctgtgcggtgattgggggcggccctggg
ccccccaacccc ttggcttgtctgacacctctctgtgcccacagACCTCACCTGCCCACCCACC
AAAGTGTACAAGCCATGCGGCCCCATACAGCCTGCCACCTGCAACTCTAGgtaagtacagggatg
gctggtgccttccctgccacccaggcctgcctgaccggtctgggggagcaggaggaggccagagg
gtgtcccactgtgggccacagatctctggggtgcccgagcccaggactccttggaaaacatcccc
tgctgctcccagatgccttagagacagagtcaggcagggggcgcatccctggccacgcctggccc
cgcatccccacctggccccgccaccgagccccacccatccccgcccatacttagccccgcccacc
cccaccccagccccacccgtccccgcccccagccccgcccaccctgagccccgcactccacccat
tcatctccttccctgctccccactgcccatcctggggactcacgtggatgacagtggaggcctcc
tggatctctaggtctcagggcctctcttgtcatcctgcagGAACCAGAGCCCACAGCTGGAGGGG
ATGGCGGAGGGCTGCTTCTGCCCTGAGGACCAGATCCTCTTCAACGCACACATGGGCATCTGCGT
GCAGGCCTGCCgtaagctccgccacctgtggcgggatacgaccctgggcccgacccaagcacaca
cagggtgggaggagccgcccaggaccatgatgtgctctcccctctccccagctgacccccgtgac
ctgttctgccccaccagagcatgggatggccccagagtggccctgaaccctgcagagcccccacg
gtcccctgaagccccacagggaggctctgtccttgagtgatcctgtgatggtccctcccctgagc
cctgcctcccaccaccatggacgaggcttccatgcactgacagctgggctcacggtgccctggcc
tgagctccagccacatctgacaccccaaaagttctccagggccttccatcccgggggaagcagg
ctccaggcctgagagcacctcccatggccagaggcccctccgcctgcaaactcagcaccctccgt
gatgccatgctgttttctttccagCCTGCGTGGACCCGATGGGTTTCCTAAATTTgtgagtggc
tccacccccacctgccctaccccaccctctcgcgagctgagggagggagggaaggagcatcccca
tcccacagggcagctgtggggcgcccgagtgtgacgtggacgtgccagtggctggtgtgcgcttc
ctgccccatcactctgggccactcgggtaccagcccgagggaggggggtggctggacagatgccca
gggttgacctgtgtctgtccaggagccctcagggaccccttgatccattccagCCCGGGGAGCG
GTGGGTCAGCAACTGCCAGTCCTGCGTGTGTGACGAGGGTTCAGTGTCGGTGCAGTGCAAGCCCC
TGCCCTGTGACGCCCAGGGTCAGCCCCCGCCGTGCAACCGTCCCGGCTTCGTAACCGTGACCAGG
CCCCGGGCCGAGAACCCCTGCTGCCCCGAGACGGTGTGCGgtaagacgctgcagagcagaggtgc
ccggcatagggtgagggggacagagccggtgcccaccaggggcctgtgggttgggcacaggaga
gcagaggagagccactgtgtcctggcgtgaccgcggcaggaccactcggcagagatggcctccag
gtgcttcattctcctcctaacgatgaggctggtgacctctggcctgcccaggagtggcccaggga
cgtgggaagcagcggggaggtggccaagcaaggggcctggagggagcccccaggggctgtgaagc
ggtcaggtcctcggggaaaagcacgcctgcgacttactctgggaacaagtggtcgggaggaggag
tgagcagcgcccagacagtggcctccatcctcccgcagTGTGCAACACAACCACCTGCCCCCAGA
GCCTGCCTGTGTGCCCGCCAGGGCAGGAGTCCATCTGCACCCAGGAGGAGGGCGACTGCTGTCCC
ACCTTCCGCTGCAgtgagcggggctggggccgggctcctgggtggcctcttgctgggggtggggg
agtgcaggatggtgggggcgctggagcacatgctccccaccacttgtcgagggcttagctccctt
tccttccagGACCTCAGCTGTGTTCGTACAATGGCACCTTCTACGGGgtaagggcacagcagtgg
gtgggtgtggccctggggcctgaacatgtgtgtgggatgccccggggctctctgagccccactcc

ttgtcttgacattcctgccctgagggccgatccgcacaggggccctggacacgtcagagctggga
catgcttgggactcaggggcaccttacgtcgacagccatgagctccacaactgctgcctctgaga
ggtcccttcaggggctcccagcaacagcctgggggcagcacacactggcctggggtccccgcctg
cccgcccagattcctacccgcccggattcctgcctgccagattcctgcccccatggggtctctgc
ccacccagattcctgcccacatggggtctctgcctgcccagattccctgcccacctgtggtccct
gtgtgcatcagctccctgcctgcctgggtccatgctcagccaggggtgcatctatgctccatctg
aggaaggaacaactccctgcaggccccattgggtcatggggaggggtcctggccctgttgcccca
ccagtgccctcagtgccaccctcccaccccttgcagGTTGGTGCAACCTTCCCAGGCGCCCTTCC
CTGCCACATGTGTACCTGCCTCTCTGGGGACACCCAGGACCCAACGGTGCAATGTCAGGAGGATG
CCTGCAACAATACTACCTGTCCCCAGgtgagacccgaggcacctgcccccaggtgagcccccgag
gcacctgcccccaagtgagacccgaggcacctgcccccaggtgagacctgagtcacccgcccccca
ggtgagcccccgaggcacctgcccccaggtgagtccctgaggcacttgcccccaagtgagacccg
aggcacctgcccccaggtgagacccgaggcacctgcccccaggtgagacctgagtcacctgcccc
caggtgagccaccaaggcacctgcccccaagtgagacccgaggcacctgcccccaggtgagccca
cagctgctgggcagacccagccctgagtcacttatcctggctccctggggttctctagctcagcc
attgctggggtcttgtttgtttccaaagggagggtatgaagtaggaggataactgaggggggtctg
ggggtgggacaggacctgcaggctgctggggacaggggctgaggtcaggttccaggaaggcaggg
gtaggcagagagaaggtgctggaaactggggtgaggctgagccgggataactgagtgggggcaac
ttcttcgggtataggcccaggcagggacagggctaaggggtcctggaccactggggtgtagacag
gagggcaggctgggccggggcatgtgctggaggagagggttagggcctgacgcccctcatgtccc
cacagGGCTTTGAGTACAAGAGAGTGGCCGGGCAGTGCTGTGGGGAGTGCGTCCAGACCGCCTGC
CTCACGCCCGATGGCCAGCCAGTCCAGgtaacagcagaggcatgtgggggcaggtctcagctccc
tccctggagaccctcacccccaatggggctctgcacaagaggtaatccctactcagcttccacac
tcacccttgcatttcagCTGAATGAAACCTGGGTCAACAGCCATGTGGACAACTGCACCGTGTAC
CTCTGTGAGGCTGAGGGTGGAGTCCATTTGCTGACCCCACAGCCTGCATCCTGCCCAGATGTGTC
CAGCTGCAGGgtgtgtgctggaggccctgcccctgcctgggagtccttgtccatcagggaggccc
aacccctgtctgggatgccctgcacagcagggaggccccacccctgctggggaggccccacccct
gcctgggaagccccacccctgcctgggaggccccgcccctgcccgggaggccatgcccctgcccg
ggtgggcccctccccaggaggccgcacccaccagggaggccccgcccacagccctgccccctgtc
tttggcccccaccagGGGAGCCTCAGGAAAACCGGCTGCTGCTACTCCTGTGAGGAGGgtaagtg
gaagccaccttcccacaccagccctccagctccagcccgtcgccacccatccattcctgcccaga
catctgcactaggcagcagtgggatgcccgcgtccagactccaccctcggtcctggagggccatg
ggaggggtggtcccatggggagggtcggcccagcaagtccaggccctcagagtgagggtccagtg
gggctccacatctgcctttcctcctcccagACTCCTGTCAAGTCCGCATCAACACGACCATCCTG
TGGCACCAGGGCTGCGAGACCGAGGTCAACATCACCTTCTGCGAGGGCTCCTGCCCCGGAGCGTC
CAAgtgagtgggctcctggccctgtgccaagagcacctgcgtgtggtgggtccgccctggcccgt
cagctggctggcagcctgctctgggtcaggagggcctacgccagctccaggaaggagggaagggg
ctccccagcagggctccccctctccacgtccattggcagaaagcctggggccgctctcctagcca
ggccagggcccggctgagccgagccacatggcacagagctccccgctgcgaacccaggtccaggt
gtccccctggggagggtgggtggaaccagagccttctgtgaagccccccatggcttgccactgtg
gttggctgtcagcaggagggccagaagggttgacccttagctagaagaggcaggaggaagggtc
aagggctgcttagggtcagcaggattggaggagctggttcagacaggggaagtgcaggccacaga
agacagagccgggagccaagggaccctgggggcccagagctgccccatcctgagaaggtgaagcc
tcacgtgcccacagagcaggccactgtggacagaagggggcggccgtctggcccaggaccccaga

ggcccatgtgtcaccatggctgggcagagaaacggcggggccaaggtggctgatgtgagggccac
cctgcgtccacagGTACTCAGCAGAGGCCCAGGCCATGCAGCACCAGTGCACCTGCTGCCAGGAG
AGGCGGGTCCACGAGGAGACGGTGCCCTTGCACTGTCCTAACGGCTCAGCCATCCTGCACACCTA
CACCCACGTGGATGAGTGTGGCTGCACGCCCTTCTGTGTCCCTGCGCCCATGGCTCCCCCACACA
CCCGTGGCTTCCCGGCCCAGGAGGCCACTGCTGTC

## Sequence S9

Refseq genome DNA sequence of human MUC6: NC_000011.10 Homo sapiens chromosome 11, GRCh38.p14 Primary Assembly (1013459-1036655), reverse strand

ATGGTCCAGCGGTGGCTGCTGCTGTCCTGCTGCGGAGCCCTGCTCAGCGCTGgtgagtgaggtcg
aggggcgccagacactgcggtgccctcagaggggccctgtgtgcgggggtctctgggccttctcg
cctcgacaactcggctggcccacatggacgctgatggacgggaggccccagctcggctccgtgac
cagcccccgctgccaggcccgggtccccccagcagccgcggtgctgggctctgccaattggggtc
agggaccccatggggcctgggcagggacgccctcctgtgagctggcacagcagccagcgggggag
gacgctcagctggagtggggtccggcactcccgcgggaacagtgggcaggtgtgctggtgcccag
ccctggcgctctccctccccgagtggggtcctgagtcctgagaggtctctggagtgtgggggaca
gaggcacagtctctgctgggccctgggcctcgtttcctagtctacagaagtgggggacgctgcct
cccctggtgtggccgccgtgggggaagagggggaaggtgtacggccatcctgggctcagaacc
gccgcaggcagcctgccctctcccccgcgctcccctacccagctcgggcaggatccaggagggac
tgacatcctggatcctctgccaaccgcctgccctgcggctgctgcaggtgaccatcagtccctcc
ccaggcccccagaaggagccagccccaaggggtccctctgtggccgcagcctgagacctggggag
acccctcttgccgaccaaggcaccagcccaagggcctctgtgttccgtgctcctccttcctctcc
aagctggcccgaccctcctccacagccgggagccccctggggtggacgctggcgtgacagagctc
gccctcggagctgctgccaacctgggcgcctcgggctcagagcacagtgggacccccccaggggt
gtcccactgtagggcgacccaagcccaccccaccccttctctcctttgaagccccttccccacca
cgccgggcaccaccccttcaaccgtgggagatgtgggggtctctgagctcgctccccaccacgccg
ggcaccaccccttcaactgtaggagatatgggggtctctgagcccgctccctaccacgccgggcac
caccccttcaaccgtgggagatgtgggggtctctgagccctcccagcggtgccctctgcttgggac
cccagcccctacctgcacacctggcacagcttggggacaagtgccaggaagtggggctaaacttg
cagcagagtgcgacccctagtccctcgggccaccaccctggagccagggagggaggtgtggtgag
gcccagagaggctggaggtctgggctgtgggcagtgcagagggtggagggccaagtgggggtgtc
cacccgtcaccccagcaaataatctctgagcgccatctctgccaacatgggagggggcctcagcca
gcagagtgtggggtcggacagacgggctgcaggcctgggccgcggtgggccgggaggcaggggtg
ggcggccgccaagcgtgcagaaggaacagtgcgcacctgccgcctcgtgctcgtccaggggctga
gaggccgggggtggcggaccaacccgcccagatgcccatgcgcctggccaagggatcagccgagg
ggaagcacagccagtctgggcaggaacgcaggagtctcccgctggacttcagagctggaggcagc
cgacttggagccagtggactcggggaacctcctgcgggtggggcagggcgagggcggcgggggc
gcagggggtgcagcaatgaggcacccacccggggcagtgaagccagcctggcagggctgagggca
ccgaaacacaggcccgaggtgtctgtgctacagcggggacggcccccaagtgccacccccccatcg
gaaaggtggagaaactgagttgcagggggcattgcccaggggagaccggtcacagctctgcagcc
tccagaggtgctggcccaggggcaggcagccaactgctcctcccgctcagagctctcccacccct
cccccaccagaggccagcgagggccccatcccaggaaccactgagttccatggctgagggttcgg
tgtgctgcgcactcctgcagcaggtgctctgagccctgtggatcatccctggaaggaatggcggg
gggctgaggaccatgacacctactgggacccccaggagggggatgagtgagggggtaggtgctcg
ccaggctcagggcccagcatgagccacccccacaccagtctggagtaaactcagcttacactggg
gataagccaggtgcaacccatcagaaaacgatctcaaggccaaaggggaccacagccaaatatgt
gtcccacatagccaaggttggcattcttcttaaatccagtcactcaaatacccaacctcctgcct

gtgccagacaggactcacccaggcggccaaggggcaggcagcctggctcctccctggaccgcggt
taacaccatctcccagctgacaggcccctgcccctgcgtcaccggagacacaggtgtccagcggt
aatgggggcagcatcaagtaagtcccagaaagtgggcagagcctggggtgagagcacaggtgagg
agtggcaggaatgggatctctgagctacatctggaaggaggtgctgtgagtgccctgctaaccaa
tgggcaggggcaaggagggtcccggccccaagaagggcctggagaggtcacgggtcctgctcagg
tgccgcaccaccactcagacatcgggggctcgtgggagccacaggaagggtcatagtctcggagg
cagggtccggggagttacccagaggtgggaaaagtctccctctgggtacaggggggtgggacgtgg
ggggtgttggccccaccccagcctctgggagtcagggtgggacgtcccccgctgccctgccctgg
agtcaccgtgactgggctatctggatgtttctctgacaacagtggcctcagtgccaggcctgaca
tcaaggctgtctcaaggctgacgtgcaggaaagcggccatgtttagagccccggagccaccgaga
gcagggagaaccggctgtggggccttggcgcagacccctcagggaaaccaggcggccaagctggg
gaggcgggcacagcccactttgttggctccgccgccaagcccctgccacccgcaccagcccctcc
tagccccacccagaagccccagggatgagtaccctgcacacagggtgcccgccatctgccgaacc
ccgagggggctgacggctgaactgggactgagtggacaaacgagggagtgaacgagagcagggaag
gaagctggggctcgtgaaggacagcatgggaggccaagcgcttggcttgctgttgaggcctcgag
ccccacgggccatggacacttctcgcacgccgagtgtgtgcagggaaacgggcggagcagccctg
agcagaggtgagcggcgccctcagggacgacggggtagccccacaccgactgcgggtcccgtcca
cacagGTCTGGCTAACACCTCCTACACCAGCCCAGGCCTCCAGAGGCTGAAGGACTCTCCACAGA
CAGgtaagaacacagcggccactgatcctgccgcccagaccacccaggcccggtgtgcaggagag
aggggcccggagcccaggtcacccccaagacacacacatccaacacacatatatgcaccaacatg
aacacacatgcacatacacccaacacacacacatacccaatatacacatgcacacacacctgaca
cacgcatacacatgtacatgacacatgcatgagcacacgtcacacacacacccaacactctacac
agacacacaacccaacacacatgcacacacacccaacacacacaacctacacacaaacacacctg
acaaacacatgcacacacacccaacacccatgcacacacatcccagacatgcaaacatgcacact
tacaacacgcacgacttacacatgtgcacgcacccaacacacacacaccctacacacacatgcac
cctatgtacacaccctacacacctgagacacgcacccaacacacacatacacaccccgaatgcac
acacatgcactcgacacacatgcccagctcacacaaacacacatgtgcatgcaacacaatcacac
acacccaacacacatgcaggtgtatatctcacacgcctacacagccctccaacacacacacaccc
acaatgcacacatggacacacctgacacgtatgcacacacccgagacacgcacatacgtgcacac
acgtgcacacacacaacacacatacgtgtacccacacacacgtgtgtgcacatgcccacagcggt
gctgactcctggccaggcatataccatagcactcaggttcatcggatgtctggggtttccacagc
ctggccccaactcaaaaccctggcccagggagacgagtgacctctctgcctgccccagccctgc
ctggccaggatggtggggacacagggctgtgtgagcactgctctctctgtagCCCCGGACAAAGG
CCAGTGCTCCACGTGGGGGGCTGGTCACTTCTCCACCTTCGACCACCACGTGTACGACTTCTCGG
GGACGTGCAACTACATCTTCGCGGCCACCTGCAAGGACGCCTTCCCCACCTTCAGTGTCCAGCTG
CGGCGAGGCCCAGACGGGAGCATCTCGCGGATCATCGTGGAGCTGGGGGCCTCCGTCGTCACTGT
GAGCGAAGCCATCATCTCAGTCAAGGACATCGGgtaggtcaggtgggccggggcccgggggctcg
gggggccggaggactgaggatcgaccaccgacttcccttgccccacagGGTCATCAGCCTGCCCTA
TACCAGCAATGGACTCCAGATCACACCCTTCGGCCAGAGCGTGCGGCTGGTGGCCaagcagctgg
agctggagctggaagTCGTGTGGGGTCCTGACAGCCACCTCATGgtgaggagagaagggccaggg
tgggcctggagacccgcagcacgggggggcaggtgggacttgggaggggctgccctccttgggggg
tcccagccagccctcaggctgccatgcagccaggcccttcgtgccaagcagtgctgacccctgac
cagcaacatgagccaggactgggttttgtgggtgactcggcttctccactggaaaattagcctcc
cataagaagaggcagatgagcaggtgggggatggtgggggctctgggtccaggagcaggaactg

agagccctccacagcagatgagggggcccctggcccccgggcccccagcagagccgtctcccgca
gGTTCTGGTGGAGCGGAAGTACATGGGTCAGATGTGCGGGCTCTGCGGGAACTTTGACGGGAAGG
TGACCAACGAGTTTGTCAGTGAGGAGGgtaggtgggggcagggctggggggcctctgggggggcc
tctaggtgggggcagggcagggggcctctgaggcccagcccccacggtgctgacccccattct
gcaccccagGCAAGTTCCTGGAACCCCACAAGTTTGCTGCCCTCCAGAAGCTGGACGACCCCGGC
GAGATCTGCACCTTCCAGGACATCCCCAGCACCCACGTCCGGCAGGCCCAGCACgtaagcaaggg
ggctccaggtggggctgaccccaggtctgacagggtcgccgagaccaccaagggcccccacaagc
agccgcatcacagacgccctgcccgacctccccagtcgtggtggcatggggtgtggcctttgccc
ccatgaccctgagtggcccccccagGCCCGGATCTGCACCCAGCTGCTGACCCTGGTGGCCCCTG
AGTGCAGCGTGTCCAAGGAGCCCTTCGTGCTAAGCTGCCAGGCGGACGTGGCCGCAGCCCCCCAG
CCAGGCCCACAGAACAGCAGTTGTGCCACCCTGTCGGAGTACTCCCGCCAGTGCAGCATGGTGGG
CCAGCCGGTCCGCCGCTGGCGGAGCCCCGGCCTGTGCTgtgagtccagggaagggagagggaggg
gcaggaaggccgagggctccagacccagctctcccagcccctggactgcctgacgtgacgcctgc
atccctcctggtctgagagacaggagatcctcgctgctgcttctagatggaggaagggctgggct
aagtcctccatcaagctggggtggggcagggaggggtgggggggtgggaccctctcaccggagc
cgcgtgtgcccacagCCGTGGGTCAGTGCCCGGCCAACCAGGTGTACCAGGAGTGCGGCTCGGCC
TGCGTGAAGACCTGCTCCAACCCGCAGcacagctgctccagctcctgcaCCTTCGGGTGCTTCTG
CCCGGAAGgtgagggcagtcgcaggcactctctctccccgggacccctagagagaacccagagac
aaggggacgtcagaccccacccaccccgacccaggtcaccccaacattctgctgcctgagtccca
gctctggcgcctcttgccctgggacaggcttttcccgcctggacgcttacagcctgcaggtcccc
cagccacggccacccctgcaaacccacgggcggtgagggctgctcactgcggtctaggagctgcc
gagggaaggcagggtttgagccgggcccaagggacagttacacgtccctgcctgctggaggccac
cactcaccaggaacctgggccgggcacattggcacgaggggctgtgcacgcccagggcctgacat
agagttgcgaatgcaagtgcagctggagagaggtcatggcccaggctagagggggcgtctccctg
gagccaggctggagggggcgtctccctggagccaggctggagggggcgtctccctggagccaggct
ggagggggcgtctccctggagccagggaggggagagtgggaggcagtcagtcccacccaagcccc
aagaagaccctctcctgcagGTACGGTCCTGAATGACCTCTCCAATAACCACACCTGCGTGCCCG
TCACCCAGTGCCCCTGTGTGCTCCACGGCGCCATGTATGCCCCCGGGGAGGTCACAATAGCTGCC
TGCCAAACCTGgtgagtgaggcgccggagggggctctggccggccggcaggggttccactggcct
agggcaggcaggggtgtgctgtctggctccagcggccctgcccaccatgccgctacccccacgcc
cacccacagCCGGTGCACCCTGGGCCGCTGGGTGTGCACGGAGCGGCCGTGCCCCGGACACTGCT
CCCTGGAAGGTGGCTCCTTTGTTACCACATTTGACGCCAGGCCCTACCGCTTCCACGGCACCTGC
ACCTACATCCTCCTCCAGgtaggacgagccctgtggcccgtggggagggggcgctcccggtgaccc
cacccgtgtctgagccccgcccctcgcagAGCCCCCAGCTTCCCGAGGACGGTGCCCTCATGGC
TGTGTACGACAAGTCCGGCGTCTCACACTCCGAGACCTCCCTGGTGGCTGTGGTCTACCTCTCCA
GGCAGgtaaggcctttcctgcgcccatccctgccagcagggctccgctccctggtgcctgcaccc
tgactcaggccaccctctctccagGACAAAATTGTGATCTCTCAGGACGAGGTGGTCACCAACAA
CGGAGAAGCCAAGTGGCTGCCATACAAGACTCgtacgtcctggcccagtctgtggcccccagag
ttgtgccttcggggcgggctgcagtgggtggggagggagcccagacggcccactgagcactgcgt
cctgcagGCAACATCACGGTCTTCAGGCAGACGTCCACCCACCTCCAGATGGCCACCAGCTTCGG
GCTGGAGCTCGTGGTCCAGCTGCGCCCCATCTTCCAGGCCTATGTCACTGTTGGGCCCCAGTTCA
GAGGTCAGACCAGAGgtgagtccctgcctctccaggtggccctgttgcctcatccctacagggtc
tggccatgaccaggggaggagaaaggtccaagagaggttcacacaaccctctgtgtccggggagc
ccccaggggtaacaggcacgtgtggcccacgcagcctctcacccagggtgctgttagaggaagga

cgggagctccagggcgtgtgggcaggaatgggtgcaggagggatgggttcaagtcgagctcacgc
cccgccggctcagGGCTCTGCGGCAACTTCAACGGGGACACAACGGATGACTTCACCACTAGCAT
GGGTATCGCCGAGGGCACCGCCTCGCTGTTTGTGGACTCCTGGCGGGCGGGGAACTGTCCGGCCG
CTCTGGAGCGTGAGACTGACCCCTGCTCCATGAGCCAGCTCAACAgtgagtgtccggcccccac
tcccctggctgcccccccagcgcccacagttctgacagacccctggactggaccacaggcccagc
tgccagggtgggggtccctgggaggagccgtagctggaatgggaggggccgggactcacgcccg
ggcctgtcatccccagAGGTGTGTGCAGAGACCCACTGCTCCATGCTGCTGAGGACAGGCACGGT
GTTCGAGAGGTGCCACGCCACAGTGAACCCTGCACCCTTCTACAAGgtgagggcccgagggcgtc
ttgggagggctgcaggggaggctgtggtggctgacaaggtctcaggcacccaagggcataggttt
gggacccccatgtccccagggcagggtgcctgccaccagcccatgctggctctctgcccgcagAG
GTGCGTGTACCAGGCCTGCAACTACGAGGAGACCTTTCCCCACATCTGTGCCGCCCTGGGCGACT
ACGTACACGCCTGCTCCTTGCGGGGCGTCCTGCTCTGGGGCTGGAGAAGCAGTGTGGACAACTGC
Agtgagtgcccgacggggcttaagcggggtcacggcaggctgggctcacgagggggtgtcctggg
accccaagctctgaggctctgcagtgcccggcaggcaggggaacccgagattgccctcccggccg
ccccctcccggagagtctgatgcccgggtcccccacagCCATCCCCTGCACGGGTAACACCACCT
TCAGCTACAACAGCCAAGCCTGTGAGCGCACCTGCCTGTCGCTGTCGGACCGTGCCACCGAGTGC
CACCACAGCGCCGTGCCCGTGGACGGTTGCAACTGCCCCGATGGCACCTACCTGAACCAAAAGGG
CGAGTGTGTGCGCAAGGCCCAGTGCCCGTGCATACTGGAGGGTTACAAGTTCATCCTGGCCGAGC
AGTCCACTGTCATCAACGGCATCACCTGgtgagggaccgggcaggggccaggcgggggtccctg
ccaggtcccggggtctcacgcagcctctctcttgcagCCACTGCATCAACGGGCGGCTGAGTTGC
CCGCAGCGGCCACAGATGTTCCTGGgtacgtacagcagcgctggccgcaggctgggactgtgagg
ggccccggcttcctctgagtgtcctgaccgcgcacccctctttcctgcagCCTCCTGCCAGGCCC
CTAAGACCTTCAAGTCCTGCAGCCAGTCCTCCGAGAACAAGTTTGGGGCAGCCTGTGCCCCCACA
TGCCAGATGCTGGCCACCGGTGTTGCCTGCgtaagggggcgcgcgaggagcagggacaatgcccg
ggccccagctgaaccatgagggggtttccacagaactccgggagacctggccgcattctgccctg
cagcgggtgcctggggctggccacgaggtggaagcggccctgtggccaggcagctggaggccccg
ggggctcgtctgcagggctccctaaggacagtgggcaccagctcggggagggtgggctgcccatg
tgtgggagtgggtacagcagcccagccgtgtgcactgggtcccaggaccttccacgaggtggggg
aagctttgctaaggcagtaagcccagctgcccacaaaggccacagagcagggctgtattcaggga
gaggacctgggagacacgccgggcagtctcagaggggcccagggctgggcagcagctggctgagg
ccaccctcccaggagctgctgacctgcctccctccagGTGCCCACCAAGTGTGAGCCTGGCTGTG
TCTGCGCCGAGGGCCTCTACGAGAATGCCGACGGGCAGTGTGTGCCCCCCGAGGAGTGCCCATGT
GAGTTCTCGGGGGTCTCCTACCCTGGAGGAGCTGAGCTCCACACTGACTGCAGGACCTGgtgaga
caaagcctcgcttcagacgccctgggctcctggggccatctggggcaggcggaggctgcccgggg
tggggctgtcccagaggcgggggcgtccagaggtgcagtctggcctctccagggaccacacccag
tatggcaggagggccgcaggcccaccacccacacctgctgtctccacagCTCCTGCTCAAGGGGG
AGGTGGGCCTGTCAGCAGGGCACCCACTGCCCATCCACCTGCACCCTCTACGGGGAGGGCCACGT
CATCACCTTCGACGGCCAGCGCTTCGTATTCGACGGCAACTGCGAGTACATCCTGGCCACGgtaa
ccatcgggtgccaggccgcaggggccggggacccagaggcacggcctccagggctcctcctcagc
gccctctccctgcagGACGTCTGTGGTGTCAACGACTCACAGCCCACCTTCAAGATCCTGACAGA
GAACGTCATCTGTGGGAACTCCGGGGTCACATGCTCACGGGCCATCAAGATCTTCCTGGGGgtga
gcagccgggcagactctggcagggcaggacgggcggtagggggccctgccacacagctggatccc
gcgcactggagcctccaggtcctgccccgagatgcagccctccccgcctgcctctccttgagggg
tgcctgctgcccctccctggccagacgctcaggaccacgggtgcttgtgccccacaaggtttcag

ggactgcagaatgtctcccgggcagaggggagggactctccaggaagcccgagcttcagccctca
gccctgttccaggcatgccaggcccttctgccccaagctggctctcagcaggcgctcctgtgcat
ggccttaacagacctgggcagctctgtctctgagagctcggtccagcacggctgtgccaggggcc
gggggagacagacaggcagtcctatgccaaccccaccccacagGGCCTGTCCGTGGTGCTGGCGG
ACAGAAACTACACGGTCACCGGGGAGGAGCCCCACGTGCAGCTCGGGGTGACGCCGGGTGCGCTG
AGCCTTGTCGTGGACATCAGCATCCCCGGGAGGTACAACCTGACGCTCATCTGGAACAGGCACAT
GACCATCCTCATCAGGATCGCCCGTGCCTCCCAGgtaccgcacgccctctgcctcctcccaggcc
ctccctgatgcaggggtggagacagatggcacggcccctgggcccggcctgatgccaccgtctgc
agGATCCCCTCTGCGGCTTGTGTGGCAACTTCAACGGGAACATGAAGGACGACTTCGAGACGCGC
AGCAGGTACGTGGCATCCAGCGAGCTGGAGTTGGTGAACTCGTGGAAGGAGAGCCCGCTGTGCGG
GGACGTGAGCTTCGTGACAGACCCCTGCAGTCTCAATGCCTTCCGGCGCTCCTGGGCCGAGCGCA
AGTGCAGCGTCATCAACAGCCAGACCTTTGCCACCTGCCACAGCAAGgtgggcaccgggcacgag
ggctgtgcgcctgcccctccacaaatgtccggtgggggcgggggaaggggcggggaagccaggca
cggttctccctccgtgggatcccagaccagccaggtcagggtgcagagagtttcagaccctctgc
ctgagtgcagggccctgatgtggggccgactcgtgcactgtggctgttaagcagcccccggcct
ccacccaccagatgccaatagcacccccagacagtgccacgagtcccctggggcagcatcgcctg
gtgggccccctgctcaggagctggtggtcagggagacctccaggatgcagggcgtgcacttccca
gcccagggcgtgatccggccatcgcggcgcagtaagaactgagaggatgggggcaccccgcgggg
gaacagcatggcactggcccgctgaatgtgagcgtgacgtgggggggagcagagaccctgggtggg
actgcagtggccgtggttagcggcagcggcatcttggtcccgatggcgggtctcaaggcgtgagc
tcgggtcaggcctccgtggcagcatcggctccgggagcgtggctagcggtcagggctgaggtcca
cgtggtgccgctcacccagtcccagcgcccttaccgccacactccggcctgaccacactgagggc
cctgcaggtggcaaagcctgatgccccgccatcccccggcagccagacactgactggcacacacc
cctgcagGTATACCACCTGCCCTACTACGAGGCCTGCGTGCGCGACGCATGTGGGTGTGACAGTG
GCGGGGACTGTGAGTGTCTGTGCGATGCCGTGGCTGCCTACGCCCAAGCCTGTCTGGACAAGGGT
GTGTGCGTGGACTGGAGGACCCCGGCCTTCTGCCgtgagtgaccacccctcccacaggttgctcc
agcccctgccacccagcgccactaagggccggcgcagggctgcccttgcccacaggcccctctcc
ctggacaggcgccgggctgtgccctgcggggccaccctggccctcccgcagcccccagctgccca
ggggaggcttcaccaagagggcccagaccttccccgcccacactcaggttccatgcatgcccagg
ggaaccaccgggtcagggccagggccagggccaggaaccccaccagctgacagctcccttcctcc
cagCCATCTACTGCGGCTTCTACAACACGCACACGCAGGACGGCCATGGCGAGTACCAGTACACA
CAGGAGGCCAACTGCACGTGGCACTAccagccctgcctctgccccagccaGCCACAGAGCGTCCC
AGGCAGCAACATCGAAGgtgccaggtgaccgggaggcaacgcagatacacaggggacccattca
ctcattcatgcacattcattcattcacacgcattcattcattcacacattcattcatgctcatta
attcacacattcattaatttgcacacattcattcacacacactcacgcacattcacattcattca
tgcagattcatgcattcattcacgcatattcattcattcacgcacatttgttcatccactcacac
acactcatgcacattcattcacacattcattcacgcagtcactacacacacattcattcatgctc
attcacgcagtcacattcccgcagattcatgcacattcattcacgcacattcactcacgcacatt
cacgcacactcatgcacattcactccattcatccattcattcacccacacacactcattcaacat
attcacgctcattcacattcattcacgcacattcactcaacacattcacattcattcatgctcat
tcacacatttactcacccacattcatgcacattcacgcactcatgcattcatttacgcacatttg
ttcactcacgcacattcactcatgcacattcacgcacattcattcaacattcactcacgcacatt
cacccatgcacattcattcacacatattcattcattcattcatgtttgcttattcattcatgcac
attcgctcatacacacacgcacattcatgcattcatttgcacattcattctttcacacacacagt

cactcagcagatgggtcctgagcacctctgtcctccaggccggctccaggtcaggggacctggcc
ttacacaaaacaaacagtcctgcaacctggagcttccctcccagccagggagaggtgcttaagga
ggcagacccagtgtcggccgaggcgcccaccatggagaggagaaaggcaggctggggtgggagag
ccacaggcttctgtgccacacagggtggttggggcccttggtgggaggcagatgtgagggcgagg
atgggaaaggtgaggaggggagccaggaaagtgtccagaggagcagccaggagcagccttgtgcc
cagaacccttaagaaagccagggcagcgggcagagccagcgagtgaggggcgcgggctgcagagg
gcagagggagcggtggaggggtgcggggctgtagagggcagatggagtgagtgaggggcgcgggg
ctgtagagggcagaaggagcgagtggggcgcggggctgcagagggcagagcaagcgagtgagggg
cacagggctgcagagggcagagggagtgagtgaggggcgcggggctgtagagggcagagggagcg
agtgaggggcgtgggctgcagagggcagagcgagtgaggggcgcggggctgcagagggcagaggg
agcgagtgagaggcgcggggctgcagagggcagagggagcgagtgaggggcaccgggctgcagag
gggtgtggggtacacagacttgtggggtggggtgtggccgggagggaggggtctttgtgaggcct
gtggctgccccaaggcagaggtctgaatgcagagggtcctgggattaacaggtcctggcccaagt
ggtggaggagggtggtgtctggagccatccggacacacagaggacagatgtgttggggctcagga
gggcaatgcagggcacctgcatcggggagcacagaagaggggccagtgtctgaggtccaggggga
ggtgaggactggaagatggcaccgaagccccagccccgggtgataagcagggtatgagagaccaa
ggcctgaggaaggcatggcaggaaaggaggggcttagagcaaggtgcgcgggtgaaactgtaatc
ccagcactctgggaggccgaggcaggaagatcacctcaggtcaagagtttgagaccagcctggcc
aacgtggcgaagccccatcactactaaaaatacaaaaattagccgggcgtcatggcacatgcctg
caatcgcagctacctgggaggctggggcaggagaatcatgtgaatccaggaggcagaggttgcag
tgagcgaagattgtgccactgcactccagcctgggcgacagagcgagactctgtctcaaaaaaaa
aaaaaaaaaaaagcagagaggaaggaggccgccagggcaggaaacacgcaggtgggcctggctgg
ccaccagtcacacagaccctggccgttctccaccctagGCTGCTACAACTGCTCCCAGGATGAGT
ACTTCGACCACGAGGAGGGGGTGTGCGTGCCCTGCAgtaagtccagtcggctgccctgagaaccc
tgcccctgcctgcatgcaggcagaacgcacgtccacatcccacaaacaagggagagagccctcgg
gggctgtgagcccttacgtgagctctgggactccactctccaggaccctggccccttgggtctcc
agagactcaggcagcctccagcccatggcagcagagagaaaaaaacacagcactgaaccccactg
ggccctcccatcccctcttccaccagctcccagctcccccctaccctcaccaggcctgccagggtg
cctggggtatagagtgggaatcatcctgggaaggagaaagggagagaggggagggtgggctccca
gcccctccagtcccctccaccacagccttggtgagcatctctgactcaggccctcggagctccca
gaggccccacggggtggggaggcttagagaaccacagggagctgaccccatcttcttgcagTGCC
GCCCACCACGCCGCAGCCACCCACCACGCCGCAGCTGCCCACCACAGgtaattgcacgcacacta
ggtgccaaggtgacgcaggccctttcctgggctcccctcggttccctggacaaatctgtcgggtg
ggaagcagggagggggcaggcagtacctgtgcccttctcaggtctaacctggcaatcagagttgg
ggaggagctcagcccacagccaggaagcagcctggtccccatggccctggctggacccacggatg
agcacagagtggggcctcaacaggtgacccccgccatgcaggaggcccagccaggagccaggctg
cactcagagtgccacaggccagccctcaactgcacatcattggcccctgcagggctgcccggctt
cagggccaagcaggcagaggggagggtgctccccaaggatatgccggtaccctgcagccctgatg
gcatgtccgcccacccagGCTCACGGCCCACGCAAGTCTGGCCCATGACGGGAACCTCCACCACC
ATCGGGCTTCTCAGCTCCACCGGACCCTCACCCAGCTCTAATCACACCCCTGCCAGCCCCACCCA
GACACCCCTCCTTCCAGCCACGCTCACATCCTCCAAGCCCACAGCCTCCTCGGGAGgtaaggagc
ctccagctgagcccatggagagggcagctgcaggaggtcctaggtacacctctggggtgggctta
gggatggccctgcttcctggctcacatctgccactaagcagattcccagcgtagaacttcctatg
cttgggagccaaactggggatttttcggaaaaacttttaaagacaagctggtcatggcatggggc

attccttgctgctggctgctgtcactagaaaccgtgtgggacctgcagggcctctgccaggcagc
ctgcccctgccccacaccgagcagggccccatctgctgcccagggaccctggaggggacaggacg
acagtccgttcagctaagcaagggtgggcgcaggagcagccgcagccccataagcatgtggtcac
tgccccagctctggccaccacagccaggagtacggtgggaggacctaacaaaggcaagaggaaga
gcccctccaaggaggctgagtcccggacagcaggccagggatcccagaagcagggcagggggctg
ggacacaagccttcgaaatgcaggcccacagcaaggggatgttccgggcggctgtcctctgcagA
ACCACCTAGACCAACCACGGCCGTCACCCCACAAGCCACATCAGGGCTGCCTCCCACAGCCACAC
TGAGATCGACAGCCACAAAACCCACAGTGACCCAGGCCACAACCAGGGCCACGGCGTCGACCGCC
AGCCCAGCCACGACGTCCACAGCTCAGTCCACAACACGGACCACAATGACACTACCAACCCCAGC
CACATCAGGGACAAGCCCCACGCTGCgtaagtcatggcgccatgggatgccagcactgccgaagg
cacccggtcccaccaccagctcacattcagtgattcagccataaagaagaccccgtatttcccag
agggcaagcgagaaggcagcccaaaagtgtaggctggagctggaggcaaggaaggccgcctggca
ctcacaaaggcaggcctggggagcagaggtgcaggagggtgggggccaccaccacggccacaggg
aaccagaaggggataaagtagggcctgggttccagtcacagagcggcagctgcactgaaggagtc
agcacgcagctcagggcaggatgtggagcaagtccaggtgagatggagacaatggggcaggctgg
agtgcccagcaggggccatgtcacaggaacagaggcacagacaggcaagaaaaaggtcacataga
caaaaggacgggccagcggaggtcagggtagagaaacaaaaacaataacgatgacaacttcacca
attcccacagCAAAATCGACCAATCAGGAACTGCCAGGAACAACGGCCACCCAGACGACAGGCCC
ACGTCCAACCCCAGCAAGCACCACAGGCCCAACCACCCCACAGCCAGGACAACCCACGAGGCCCA
CAGCCACAGAGACCACTCAAACAAGAACGACTACTGAATACACAACGCCCCAaaccccacacacc
acacactccccGCCTACGGCGGGGAGTCCCGTCCCTTCCACAGGTCCTGTCACTGCAACATCTTT
CCATGCCACCACTACCTATCCAACCCCATCACACCCTGAGACCACACTTCCCACTCACGTTCCAC
ctttctccacctccttggtGACTCCAAGTACTCACACAGTCATCACCCCTACCCACGCACAGATG
GCCACATCTGCCTCCAACCACTCAGCGCCAACAGGTACCATTCCTCCACCAACAACGCTCAAGGC
CACAGGGTCCACCCACACAGCCCCACCAATAACGCCGACCACCAGTGGGACCAGCCAAGCCCACA
GCTCATTCAGCACAAACAAAACACCTACCTCGCTACATTCACACACTTCCTCCACACACCATCCT
GAAGTCACCCCAACTTCTACTACCACGATTACTCCCAACCCCACTAGTACACGCACCAGAACCCC
TGTGGCCCACACCAACTCAGCCACCAGCAGCAGGCCACCACCACCCTTCACCACACACTCCCCAC
CTACAGGGAGCAGTCCCTTCTCTTCCACAGGTCCCATGACGGCAACATCCTTCAAGACCACCACT
ACCTATCCAACCCCATCACACCCTCAGACCACACTTCCCACTCACGTTCCACCTTTCTCCACCTC
TTTGGTGACTCCAAGTACTCACACAGTCATCACCCCTACCCATGCACAGATGGCCACTTCTGCCT
CCATCCACTCAATGCCAACAGGCACGATTCCTCCACCGACAACGCTCAAGGCCACAGGGTCCACC
CACACAGCGCCAACAATGACGCTGACCACCAGCGGGACCAGCCAAGCCCTGAGCTCATTAAACAC
AGCCAAAACCTCTACATCCCTACATTCACACACTTCCTCCACACACCATGCTGAAGCCACCTCAA
CTTCTACCACCAAcatcacccccaaccccaccagtACAGGAACCCCACCAATGACAGTGACCACC
AGTGGGACCAGCCAATCCCGAAGCTCATTTAGCACGGCCAAAACCTCTACATCCCTACATTCACA
CACTTCCTCCACACACCATCCTGAAGTCACCTCAACTTCTACCACCAGCATCACCCCCAACCACA
CCAGTACAGGCACCAGAACCCCTGTGGCCCACACCACGTCGGCCACCAGCAGCAGGCTACCCACA
CCCTTCACCACACACTCCCCACCTACAGGGACCACTCCCATCTCTTCCACAGGTCCTGTCACTGC
AACATCCTTCCAGACCACCACTACCTATCCAACCCCATCACACCCTCACACCACACTTCCCACTC
ACGTTCCATctttctccacctccttggtGACTCCAAGTACTCACACGGTCATCATCCCTACCCAC
ACACAGATGGCCACTTCTGCCTCCATCCACTCAATGCCAACAGGCACCATTCCTCCACCGACCAC
GATCAAGGCCACAGGGTCCACCCACACAGCCCCACCAATGACACCGACCACCAGTGGGACCAGCC
AATCCCCAAGCTCATTTAGCACGGCCAAAACTTCTACATCCCTACCTTACCACACTTCCTCAACA

CACCATCCTGAAGTCACCCCAACTTCTACCACCAACATCACCCCCAAACACACCAGTACAGGCAC
CAGAACCCCTGTGGCCCACACCACCTCGGCCAGCAGCAGCAGGCTACCCACACCCTTCACCACAC
ACTCCCCACCTACAGGGAGCAGTCCCTTCTCTTCCACAGGTCCTATGACTGCAACATCCTTCCAG
ACCACCACTACCTATCCAACCCCATCACACCCTCAGACCACACTTCCCACTCACGTTCCACcttt
ctccacctccttggtGACTCCAAGTACTCACACAGTCATCATCACTACCCACACACAGATGGCCA
CTTCTGCCTCCATCCACTCAACGCCAACAGGCACCGTTCCTCCACCAACAACGCTCAAGGCCACA
GGGTCCACCCACACAGCCCCACCAATGACAGTGACCACCAGTGGGACCAGCCAAACCCACAGCTC
ATTCAGCACAGCTACAGCCTCTTCTTCCTTCATATCCTCCTCGTCTTGGCTGCCTCAGAACTCTA
GCTCAAGGCCACCGTCATCACCTATCACCACACAACTCCCCCACTTGAGTTCTGCAACCACTCCT
GTTTCCACAACTAATCAGCTGtcctcctcatttctcccaGTCCTTCTGCCCCCTCTACTGTTTC
TTCTTATGtgccctcctcccactcctctcCCCAGACTTCATCGCCTTCTGTTGGCACATCTTCCT
CTTTCGTGTCCGCCCCCGTGCACTCCACAACCCTGAGCTCGGGGTCACACTCCTCATTGTCCACT
CATCCCACGACTGCATCAGTGTCTGcatctcctctttttccttcttctccagcTGCCTCTACTAC
CATTAGGGCCACTCTCCCCCACACTATCTCCTCTCCTTTCACCCTCTCTGCTCTACTCCCCATAT
CCACTGTTACCGTGTCTCCCACCCCATCCAGCCACCTAGCCTCCAGCACCATTGCATTTCCGTCC
ACGCCCAGGACCACGGCCAGCACCCACACCGCCCCTGCCTTCTCCTCTCAGTCCACCACCTCGCG
GTCCACTTCTCTCACCACCCGAGTTCCCACATCAGGCTTTGTGTCACTCACCTCGGGGGTGACGG
GTATCCCCACCTCTCCAGTCACCAACCTTACCACCAGGCACCCTGGTCCCACCTTGTCGCCTACC
ACACGGTTCCTGACCAGCTCCCTCACTGCCCATGGAAGCACCCCTGCTTCTGCCCCGGTATCTTC
TCTCGGGACACCTACGCCCACCTCACCCGgtaagtggcatctctgtggcccttctcctggcctca
cctctgtgctcatgactcccaggcagcccctgcctcagcttacccctcatggtcctgtgatccca
ggaccacacgcctgttccctgccttccctacacacctgccaggcgtttccactctcctctcctgg
gcaccctctatgccaacgctgtcctggccaagcaagtgcctggcctccccgttgggccttgttcc
ccagctaaccttgacctttccctgcactggctcactcatgcccggtcactgctgcactcctggcc
tcccctcagagtgttacctgccaaaagccttccctggctgcccctttggttgctcagaggatgtc
agggctggcccaaccccagggttcaagcagcacagcccttcctccaagtgcagagagagacacaa
aagtctggcacatcccctgcctcgccccaaaggacctgttgaaggtggagctggacctggcaacc
agggcacctgctccttcgtgctcatgccaattctctgggccacggtctcttcccggcccatgcct
gtgagcgagtccactgctccccaggccatgggatgcctggggacagagcacacctcaggccttat
ccatgtccacctcacctcacctcgcgttccagggatgtccctcccttccctcctgggtgttcctc
tcacagcaaccacccagggactgggtccctcgcatgcatcgacctccaccaggcgacaacgccac
agttgccttcgtggtctctcacgtgggtggcagctcgttgctgcaagctgagggaatcttggttc
gggtccctccctgagaccgggacttgggtgcaaggtgtaaccagggaggtgaccccaagaagcag
aggcgagggagcaggaaccagctgggaggggagggcagctggggacggcagggcctatggaagca
cccagagtcctgaccctcccggagaaagccctctgcagcgggcaggtaggctccaggtgacctgt
cttctctgcagccaggaaacgaagctgggagcagccaggggagggcacacgggcagcagagtcca
gcctttaccccaggactccttttccacttttaaacccacgctctccagtggcggtttctaatgcc
aacgctgccactgcctgcatcggtccaccctgctctgtgctgcctgccacgtacccaggcctctc
gctgaggctggctgtctctgggccttccccgcgggcccccaccccgccccttccaggagcccaga
ccacggggcctgctccacttccctcctccccagctggggcacacggagctcctgcaacaccatat
gtcccgctctggggtcactggcacggtgtcctcggccccccctgcaggtcctgggtctggccaacc
agagaggcccatgcccacaggtcagtgttgaggcctggggttggtcatcagggaagctggggccc
aagggtggctcacacaggcccagggctcagtatctgctgagtccgctgagaaaattctctacctt
atggactccagcagagcgactttgaggggaaaaaacaaactaggatgcggctggcctttgccttt

gtgtatggctgaggtctgagctctgtggggctccacaaggggagaccacaccttgggcaggagag
tccagagacaggtggggaccccggtctctagccagggtttccctccctcctcgctccatgcccac
cctgggcgctgctgaccgtccactcggctaccagGGGTCTGCAGTGTGCGGGAGCAGCAGGAGGA
GATCACGTTCAAGGGGTGCATGGCGAACGTGACGGTAACCCGCTGTGAGGGCGCCTGCATTTCCG
CTGCCAGgtgagtccacaggtgggaggcctgccccacgtccaccaaggtgctcacaaccccaccc
cagagaggttcgggcaccccacccaggcgcctgctgccccatcaggtgagccactctgcgctcca
tctgcccaacaccgccctggcccctgtgagccgggagctcagcggggaggtccaggcctctgcgg
agcagctgccctggggagggaggggccttatgcccctgcagcagtcatgaccctgctctgtcttg
acttctcggcagCTTCAACATCATCACCCAGCAGGTGGATGCCCGCTGCAGCTGCTGCCGCCCCC
TCCACTCCTATGAGCAGCAGCTGGAGCTGCCCTGCCCCGATCCCAGCACGCCTGGCCGGCGGCTC
GTACTCACCCTGCAGGTGTTCAGCCACTGCGTGTGCAGCTCTGTGGCCTGTGGAGAC
